# Honey bee colony‐level exposure and effects in realistic landscapes: An application of BEEHAVE simulating clothianidin residues in corn pollen

**DOI:** 10.1002/etc.4314

**Published:** 2019-01-07

**Authors:** Amelie Schmolke, Farah Abi‐Akar, Silvia Hinarejos

**Affiliations:** ^1^ Waterborne Environmental Leesburg Virginia USA; ^2^ Valent U.S.A. Dublin California USA

**Keywords:** Pesticide risk assessment, Honey bee colony model, Mechanistic effects modeling, Landscape composition

## Abstract

Discerning potential effects of insecticides on honey bee colonies in field studies conducted under realistic conditions can be challenging because of concurrent interactions with other environmental conditions. Honey bee colony models can control exposures and other environmental factors, as well as assess links among pollen and nectar residues in the landscape, their influx into the colony, and the resulting exposures and effects on bees at different developmental stages. We extended the colony model BEEHAVE to represent exposure to the insecticide clothianidin via residues in pollen from treated cornfields set in real agricultural landscapes in the US Midwest. We assessed their potential risks to honey bee colonies over a 1‐yr cycle. Clothianidin effects on colony strength were only observed if unrealistically high residue levels in the pollen were simulated. The landscape composition significantly impacted the collection of pollen (residue exposure) from the cornfields, resulting in higher colony‐level effects in landscapes with lower proportions of semi‐natural land. The application of the extended BEEHAVE model with a pollen exposure‐effects module provides a case study for the application of a mechanistic honey bee colony model in pesticide risk assessment integrating the impact of a range of landscape compositions. *Environ Toxicol Chem* 2019;38:423–435. © 2018 The Authors. Environmental Toxicology and Chemistry published by Wiley Periodicals, Inc. on behalf of SETAC.

## Graphical abstract

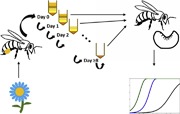

## INTRODUCTION

Insecticides including neonicotinoids are applied to control insect pests in agricultural fields and other contexts. As a result of their toxicity to insects, concerns have been raised about potential impacts on honey bees (Blacquière et al. 2012). The toxicity of clothianidin to honey bees has been evaluated in standard laboratory tests on adult bees and honey bee brood where toxicological endpoints such as median lethal dose and no‐observed‐adverse‐effect level were derived after a single (acute) exposure or repeated (chronic) exposures, respectively (US Environmental Protection Agency 2016b). However, effects on individual adult bees or individual larvae tested under laboratory conditions do not directly translate into effects on honey bee colonies under more realistic field exposure conditions. In higher tier studies designed to assess colony‐level impacts from exposure to treated, bee‐attractive, flowering crops (e.g., oilseed rape or corn), effects on colony condition (measured in number of adult bees, broods, and honey storage in the hive) and overwintering survival were not shown (Pilling et al. 2013; Cutler et al. 2014) or were inconclusive (Balfour et al. 2017; Woodcock et al. 2017), with both negative and positive effects found of neonicotinoid‐treated oilseed rape on honey bee colonies, dependent on the country where the study site was located.

Nectar and pollen collected by honey bees do not occur homogeneously across the landscape around a hive but vary spatially among different crops and land covers, and temporally with floral resource availability. Accordingly, pesticide residues reaching the colony depend on the spatial and temporal patterns of the forager bees of a colony and on the pattern of consumption of exposed nectar and pollen by the colony. Foragers only consume a fraction of the nectar they collect while foraging and do not eat the collected pollen themselves. In the hive, collected nectar and pollen are stored in cells (Winston 1987). In each cell, nectar or pollen from diverse sources may be mixed with nectar and pollen collected by other foragers that might be of different origin. Thus fresh nectar and pollen stores are a reflection of the resources currently exploited by the foragers of the colony across the landscape around the hive. Nectar or pollen containing pesticide residues may be mixed with nectar and pollen without residues, effectively diluting pesticide concentrations.

Consumption of the stored nectar and pollen by bees and larvae in the hive occurs between time of collection and up to weeks or months later, dependent on the needs of the colony. Rates of ingestion also differ among castes in the colony. For instance, foragers have high demands for nectar (carbohydrates) to fuel their foraging flights. Pollen is the primary source of protein, lipids, sterols, minerals, and most vitamins for bees (Seeley 1995; Brodschneider and Crailsheim 2010). Young adult workers consume more than 80% of all stored pollen to complete their adult development and redistribute pollen to larvae and other adult bees in the form of royal jelly and bee bread (Crailsheim 1992; Naiem et al. 1999). Larvae and adult bees may also differ in their sensitivity to pesticides. Accordingly, the level and duration of exposure to pesticide residues in nectar and pollen in the colony are dependent not only on pesticide concentration in nectar and pollen from a given field but also on the needs of the colony, other resources available at time of flowering of the treated crop, and the current and future consumption patterns of the collected nectar and pollen in the hive.

Addressing exposure and effects relationships on honey bee colonies in field experiments across multiple crops and landscapes in varying geographical regions can be very costly and time consuming. Also, potential impacts from residues of pesticides in nectar and pollen or other routes of exposure to honey bee colonies may be confounded by additional environmental factors. Honey bee colony models can provide tools for colony‐level risk assessments in addition to field studies (Becher et al. 2013; Sponsler and Johnson 2017; Kuan et al. 2018). The mechanistic honey bee colony model BEEHAVE (Becher et al. 2014) combines processes in the colony, including brood development, nursing, and mite infestations, with foraging for nectar and pollen in the landscape. Accordingly, the model can be used to explore explicit stressors affecting a colony in the context of complex colony dynamics and their interaction with the resource availability in the surrounding landscape and other environmental conditions (Rumkee et al. 2015; Becher et al. 2016; Horn et al. 2016; Thorbek et al. 2017a, 2017b).

In the present case study, we applied BEEHAVE to assess potential effects of different clothianidin residue levels in corn (*Zea mays*) pollen to honey bee colonies. The simulated residue levels were 0, 12.2, 39.9, 200, and 2800 ng clothianidin/g pollen. The intermedium level of 39.9 ng/g corresponds to the highest residue concentration of clothianidin measured in corn pollen, and resulted from test fields treated via soil application with clothianidin (Ampex® insecticide; Valent U.S.A.) in large‐scale fields located in agricultural landscapes of the US Midwest (Wisconsin, Minnesota, and South Dakota). We added an exposure‐effects module to BEEHAVE that makes it possible to represent fields providing exposed pollen in realistic spatial and temporal landscape contexts. Effects to broods and adult bees in the hive are modeled temporally explicitly and are applied using dose–response relationships for larvae and adult bees derived from clothianidin laboratory toxicity studies. The goal of the application of BEEHAVE with the pollen exposure‐effects module was to assess potential effects of different residue levels in pollen from a central cornfield to colonies over the course of the foraging season into the spring of the following year. In addition, we evaluated the role of landscape composition on pollen collected (residue exposure) from the central cornfield and the resulting effects on colonies in realistic agricultural landscapes of Wisconsin, Minnesota, and South Dakota.

## MATERIALS AND METHODS

### Pollen exposure‐effects module in BEEHAVE

Developed by Becher et al. (2014) and implemented in NetLogo (Wilensky 1999), BEEHAVE is a model of a honey bee colony. The model links the simulation of processes in the colony with the resource availability in the landscape. The landscapes around bee hives can be represented in BEEHAVE as distinct patches that are defined by their distance from the hive, their daily nectar and pollen availability, nectar sugar concentration, and the gathering times of nectar and pollen. Both spatial and temporal resource availability in landscapes can be displayed explicitly in BEEHAVE (Becher et al. (2014), 2016).

We adapted BEEHAVE (BEEHAVE_BeeMapp2015.nlogo; Becher 2015) to explicitly model the link between exposure to pesticide residues in pollen to effects on the colony. Because the present study is focused on cornfields, we limited the exposure‐effects module in BEEHAVE to residues in pollen, given that corn does not produce nectar, and effects on larvae and adult bees are caused by its consumption.

With the implementation of the pollen exposure‐effects module in BEEHAVE, we followed the conceptual honey bee colony model for pesticide risk assessment introduced by the European Food Safety Authority (2016). We simplified the model concept where data were missing to provide information on details proposed and to allow for the implementation within BEEHAVE. A detailed description of the implementation of the pollen exposure‐effects module and the adapted BEEHAVE code can be found in Supplemental Data, Section 2.

Exposures were included in the model as residue levels (ng a.i./g) in the pollen in a given patch and on a given day. When simulated foragers collect pollen, the pollen load brought back to the colony carries the residue level as defined for the patch foraged on. Given that the application method of clothianidin in corn is via soil application and/or seed treatment only, contact exposure of foragers collecting pollen was considered negligible and effects were assumed to exclusively occur because of consumption of pollen containing pesticide residues.

Honey bees store pollen in designated cells in the hive. If more pollen is collected than consumed, it is processed into bee bread, and full bee bread cells are capped for later consumption. BEEHAVE does not simulate a temporal (or spatial) pattern of pollen storage in the colony but assumes homogeneous pollen storage by total weight of pollen present in the colony. Pollen containing pesticide residues would be mixed (diluted) with the complete simulated pollen stores present in the colony. In the conceptual honey bee colony model for pesticide risk assessment proposed by the European Food Safety Authority (2016), it is emphasized that the temporal and spatial storage patterns of pollen in the colony result in variability in residue levels in stored pollen (or bee bread). Pollen stored in some cells could contain high residue levels, whereas other cells may not contain any residues—potentially resulting in different effects on consumers (larvae and adult bees) from average residue levels across the whole pollen stores present in the colony (Rumkee et al. 2017).

**Figure 1:**
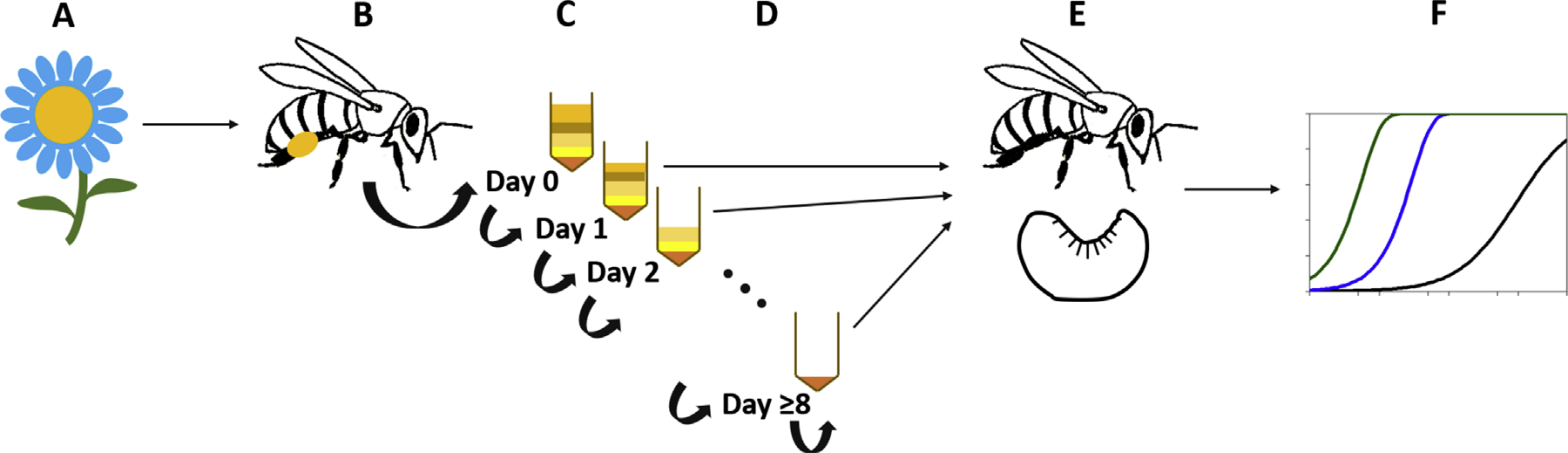
Overview of pollen exposure‐effects module added to BEEHAVE. (**A**) Foragers collect pollen from a resource patch that may contain residues of a pesticide. (**B**) Foragers add pollen to the pollen store collected on the same day (0‐d pollen age). (**C**) All pollen gathered in a single day is pooled into a daily pollen cohort; residue levels are adjusted with each pollen load added, resulting in an average residue concentration across all pollen collected in a single day. (**D**) Pollen 1 d and older is consumed by larvae and adult bees. Pollen stores are represented as daily cohorts up to 7 d old; all older pollen is pooled into one cohort; no fresh pollen is added to pollen store cohorts 1 d and older. (**E**) Larvae and adult bees prefer fresher pollen over older pollen; rate of pollen consumption depends on life stage. (**F**) Effects caused by cumulative exposure to residues via pollen ingestion and dose responses derived from laboratory toxicity tests are applied to larvae and adult workers.

In the adapted BEEHAVE version used in the present study, collected pollen is stored in daily cohorts (Figure 1). Pollen brought into the colony on a given day is assumed to be completely mixed. In honey bee hives, pollen pellets are added to uncapped cells with fresh pollen, resulting in pollen from various workers and sources stored in a single cell (Carroll et al. 2017).

No new pollen is added to pollen cohorts older than 1 d. All pollen older than 7 d is pooled into a single cohort according to the concept of the European Food Safety Authority (2016). Pollen is consumed if it is 1 d old or older (i.e., pollen is not ingested on the same day that it is collected). Bees in the colony are simulated to consume 1‐d‐old pollen first. Bees shift to ingesting 2‐d‐old pollen if no 1‐d‐old pollen is available, and work their way through the pollen store age cohorts according to availability. The oldest pollen is consumed last. The preference of bees for the consumption of fresh pollen over older pollen originated from the findings of Anderson et al. (2014) and Carroll et al. (2017).

Bees in BEEHAVE are denoted as age cohorts from egg stage through the age of first foraging. In the adapted model, each larval age cohort keeps track of the amount of pesticide residue (in nanograms of clothianidin) consumed each day by a larva throughout its development (6 d). On the day of pupation, the cumulative amount of pesticide consumed by a larva in the cohort is applied to a dose–response function for survival. The number of larva entering pupal stage in the age cohort is reduced according to the dose–response relationship.

Adult bee cohorts also store the amount of pesticide ingested on each day of their lives starting on the day of emergence from the pupal stage. At the end of every simulated day, the cumulative amount of pesticide consumed in the previous 2 d is applied to a dose–response function for acute effects on adults; and the cumulative amount of pesticide consumed in the previous 10 d is applied to a dose–response function for chronic effects on adults. The number of adults in each age cohort is reduced accordingly. Dose responses in survival are applied to both worker and drone larva and adult cohorts. Sublethal effects are not simulated in the present study.

### Pollen consumption rates

In BEEHAVE, daily pollen consumption rates assumed for larvae and adult bees show a considerable discrepancy with the daily pollen consumption rates assumed by the US Environmental Protection Agency (2014) for lower tier pollinator pesticide risk assessments. In the simulations presented, we apply pollen consumption rates derived from BeeRex (US Environmental Protection Agency 2014; Table 1). The values assumed in BeeRex had to be transformed to rates as used in BEEHAVE where daily consumption rates are applied as uniform across each life stage (larva and adult). In BeeRex, jelly or pollen ingestion rates are provided for each day of larval development, and as daily rates across the period a worker fulfills a given task. We assumed that jelly contains 18% protein (Sabatini et al. 2009) and pollen contains 20% protein (Hrassnigg and Crailsheim 2005). Because in BEEHAVE larvae are simulated to consume their protein directly from pollen (i.e., pollen consumption is used as a proxy for protein ingestion), we calculate the pollen consumption corresponding to the assumed daily jelly ingestion from BeeRex. Adult worker bees are depicted in BEEHAVE as 2 distinct age‐related task stages: in‐hive bees and foragers. We use the average daily pollen consumption rate of workers across all in‐hive task stages from BeeRex as the daily pollen ingestion rate of in‐hive bees in BEEHAVE. The forager daily consumption rate is directly used from BeeRex.

**Table 1:** Daily pollen consumption rates applied in the simulations compared with BEEHAVE default assumptions^a^

	BEEHAVE default values (mg/day)	Applied values from BeeRex (mg/day)
Worker larva	23.67	6.53
Adult worker (in‐hive bee)	1.5	6.5
Adult worker (forager)	1.5	0.041
Drone larva	50	5.7
Adult drone	2	0.0002

^a^ Derived from BeeRex model; US Environmental Protection Agency 2014.

### Dose–response relationships

Dose–response functions were derived from data available from 3 laboratory toxicity studies conducted to assess toxic effects of clothianidin to honey bees following standardized guidelines for chronic larval, and acute and chronic adult studies (US Environmental Protection Agency 2016b, 2017). We used the Benchmark Dose Software (US Environmental Protection Agency 2016a) to fit dose–response functions to the data available for the 3 studies. In Benchmark Dose Software, a range of functions are fit to the data and the goodness of fit is compared. The log‐logistic function provided the best fit to the larval chronic study data and the Weibull function delivered the best fit to the data from the adult acute and chronic studies. We implemented the dose responses appropriately in BEEHAVE. Details of the dose–response fitting are given in Supplemental Data, Section 3.1.

### Landscape representation

**Figure 2:**
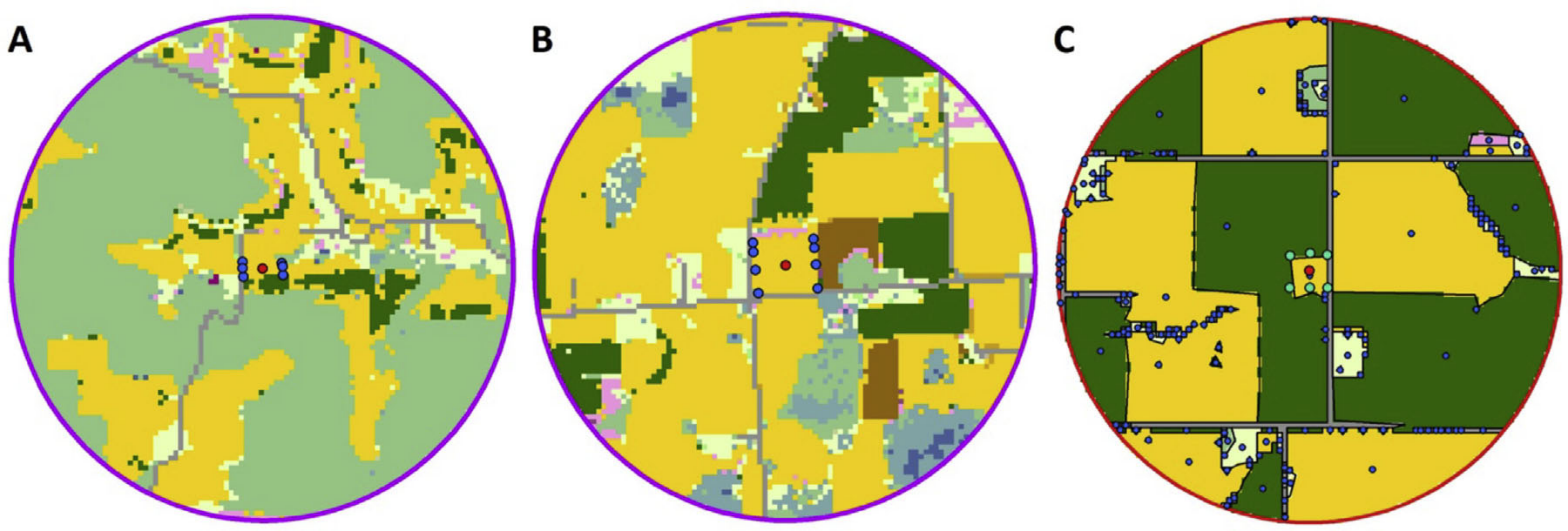
Examples of land cover compositions surrounding 3 (1.5‐km radii) of the 13 study sites provided as landscape input to BEEHAVE. (**A**) WI‐02. (**B**) WI‐08. (**C**) SD‐03. Red dots mark simulated locations of honey bee colonies; blue (A, B) or green (C) dots indicate extent of the central test cornfields; in C, blue dots indicate centroids of patches. Land covers are identified with different colors: yellow for corn, dark green for soybean, and light green for pasture. Land cover maps with legends for all 13 sites are shown in Supplemental Data, Figure S2. WI = Wisconsin; SD = South Dakota.

To set exposure to residues of clothianidin in corn pollen in the context of a realistic landscape, we compiled the landscape resource input files for BEEHAVE using land cover data from 13 corn‐growing area locations across Wisconsin, Minnesota, and South Dakota. The locations were part of an Experimental Use Permit program (US Environmental Protection Agency No. 59639‐EUP‐18) to determine the potential exposure to pollinators of the soil‐applied clothianidin product Ampex insecticide. The objective of the Experimental Use Permit was to quantify residues of clothianidin and its metabolites in pollen collected from corn plants after an in‐furrow application to untreated seeds and an in‐furrow application to clothianidin‐treated seeds in 2016 and 2017 (Valent U.S.A., unpublished data). We retrieved data on land cover for 2016 from the Cropland Data Layer (CDL; US Department of Agriculture 2016) for a radius of 1.5 km around each location. All locations represented the centroids of experimental cornfields in landscapes with varying proportions of agricultural land cover. As an example, Figure 2 depicts land cover compositions around 3 of the 13 tested fields in Wisconsin (WI) and South Dakota (SD): a semi‐natural–dominated land cover (A, WI‐02), a mixture of semi‐natural and agriculture land cover (B, WI‐08), and an agriculture‐dominant land cover (C, SD‐03). Maps of all the land covers surrounding the 13 locations are shown in Supplemental Data, Figure S2.

Cropland Data Layer information gives the land cover in every 30 × 30 m^2^ pixel of the landscape based on satellite data. The CDL specifics were translated to patches as used for BEEHAVE input by assigning all adjacent CDL pixels with identical land covers to the same patch. Central test cornfields were displayed as a separate patch (with a distance of 0 m to the colony), even if they were adjacent to more pixels with corn land cover. The area of each patch was calculated as the sum of the pixels, and the distance to the colony (located in the center of the central test cornfield) was measured from the centroid of the patch. Land covers with very low area across all sites (<1 ha at <3 of the 13 sites) and pixels classified as barren, developed, or open water in CDL were not represented in BEEHAVE landscape resource inputs. Crops not providing bee resources according to the classification by the US Department of Agriculture (2015) were excluded from representation of the landscape as well. Because land covers were excluded from the landscape resource input files, the total area of patches represented in the files was smaller than the total area within the 1.5 km radius (7.07 km^2^). For more information on land covers excluded from BEEHAVE input, refer to Supplemental Data, Table S2.

For the remaining land covers, nectar and pollen availabilities per meter squared were estimated across the year. For crops, nectar and pollen production per flower were derived from the literature along with nectar sugar concentration, flowering period of the crop, and the flower density, utilizing an approach similar to that of Becher et al. (2016). The nectar and pollen availability from each patch was calculated by scaling the values to the patch size. Crops that provided honey bee resources in the landscapes were alfalfa, beans and peas, buckwheat, corn (pollen only), sorghum and millet (pollen only), and soybeans. Beans and peas were assumed to supply the same amount of bee resources per area because detailed data for the individual crops were not available. The same applied to pollen availability from sorghum and millet. Estimates derived from the literature for these crops are described more fully in Supplemental Data, Section 3.3.

In semi‐natural land covers, various plant species may be flowering at different times of the year in varying densities. Consequently, bee resource availability cannot be directly derived from nectar and pollen production of single flowers, as it is conducted for crops. Instead, we assigned categories of resource availability to the semi‐natural land covers according to qualitative data from ground surveys of plant species present and their flowering times, and assumed uniformity across each month and land cover. Semi‐natural land covers surrounding the simulated sites included herbaceous wetland, woody wetland, deciduous forest (combined with mixed forest), evergreen forest, shrubland, hay (non‐alfalfa), and pasture (combined with fallow/idle cropland). The assignment of resource category and the resource availability assumed for each category are discussed in Supplemental Data, Section 3.3.

### Weather

Weather data were retrieved from the closest weather station for each of the 13 locations in the study for 1 January through 31 December 2016 (US National Oceanic and Atmospheric Administration 2017). Sunshine hours are not routinely available from weather stations in the United States. To estimate foraging hours available to the bees in the model, we applied the simplifying assumption that the simulated honey bees do not forage on days with any precipitation. For all days without precipitation, the full day length was available for foraging if the maximum temperature reached ≥ 15 °C, in accordance with the default temperature assumption in BEEHAVE. Note that from the 366 d of weather data available for 2016, the first 365 d were applied to simulate the weather across the year. For the second simulated year (1 January–24 April), the first 115 d of weather data from 2016 were applied.

### Simulations

We employed 2 scenarios in the simulations: a baseline scenario with BEEHAVE settings resulting in good conditions for the bee colonies, and a stress scenario with a settings outcome of lower adult bee numbers and honey stores. In the baseline scenario, colonies were not infested with mites, no honey harvesting occurred, and pollen and nectar gathering times for all land covers and resource categories were set to 600 and 1200 s, respectively. In the stress scenario, colonies were initially infected with 2000 mites all carrying the deformed wing virus, honey harvest and colony feeding by a beekeeper were assumed, and pollen and nectar gathering times were set to 1200 and 2400 s, respectively, for all semi‐natural land covers and resource categories, simulating resource patches of lower quality. Parameter settings applied in the baseline and stress scenarios are listed in Supplemental Data, Section 7.1.

Exposure to clothianidin was simulated to originate exclusively from the test cornfield in which the colonies were assumed to be located. We tested 5 residue levels in the corn pollen along with controls (no residues simulated). Residue levels were assumed constant across the central test cornfield and flowering period. In Table 2, residue levels applied in the simulations are listed. Residue levels were chosen to represent suggested acceptable residue level thresholds in a recent published clothianidin preliminary risk assessment (US Environmental Protection Agency 2017), the maximum exposure level measured in pollen among all available corn residue studies (soil‐applied and/or treated corn seeds), and unrealistically high exposures with observed high effects on individual adult bees or larvae in laboratory toxicity tests. Residue levels determined in pollen from treated cornfields ranged between <1 to 39.9 ng clothianidin/g pollen, with most measured residues <6 ng clothianidin/g pollen (US Environmental Protection Agency 2017; Valent U.S.A., unpublished data).

**Table 2:** Residue levels of clothianidin applied to pollen from simulated central test cornfields

Residue concentration in pollen from central test cornfield (ng clothianidin/g pollen)	Description
0	Control
12.2	Colony LOAEC for pollen/bee bread exposure proposed in USEPA preliminary risk assessment^a^
19	Colony NOAEC for nectar exposure proposed in USEPA preliminary risk assessment^a^
39.9	Highest residue concentration of clothianidin measured in pollen from test cornfields treated with clothianidin (Ampex insecticide; Valent U.S.A.) via soil application^b^
200	48‐h LC100 (lethal concentration 100%) for individual adult honey bees in acute adult laboratory toxicity study
2800	21‐d LC50 (lethal concentration 50%) for individual larva in chronic larval laboratory toxicity study

^a^ US Environmental Protection Agency 2017.

^b^ USEPA No. 59639‐EUP‐18 program results; Valent U.S.A., unpublished data.

LOAEC = lowest‐observed‐adverse‐effect concentration; USEPA = US Environmental Protection Agency; NOAEC = no‐observed‐adverse‐effect concentration; EUP = Experimental Use Permit.

For each combination of scenario, location, and residue level, we conducted 20 repetitions with different random number seeds (2 scenarios × 13 sites × 6 residue levels × 20 repetitions = 3120 total simulations conducted). Number of adult bees, eggs, larvae and pupae present in the hive, and honey (kilograms) and pollen (grams) stores were collected throughout the simulated period corresponding to 1 January of year 1 through 24 April of year 2. Note that landscape resource and weather inputs from 2016 were used for both simulated years. Overwintering deaths of colonies were not simulated (i.e., all colonies with any number of adult bees and honey stores present in the hive were considered alive), in contrast to default settings in BEEHAVE where less than 4000 bees present in the colony on 31 December results in colony death. Without the colony size threshold for overwintering, quantitative comparisons of colony size measures in April were possible. With the threshold, the stress scenario would have resulted in high overwinter losses of simulated colonies in control simulations.

### Data analysis

#### Colony dynamics

Colony dynamics were compared between the 2 scenarios applied (baseline and stress) among the simulated residue levels in pollen from the test cornfield and across the 13 sites. Averages and ranges from the sets of 20 repetitions were graphed over time for adult bee and brood numbers, and honey and pollen stores.

#### Colony‐level effects

Of the measures available from BEEHAVE simulations, we chose the number of adult worker bees on 2 dates, 21 October (year 1) and 1 April (year 2), for quantitative analysis of colony‐level effects. Colonies cease foraging and brood‐raising in the fall as temperatures and resource availability decline. Adult bee numbers present in the hive at that time are a measure of colony strength before overwintering. In early spring, adult bee numbers are an indicator of colony growth after onset of foraging and brood‐raising in the new season. In addition, adult worker bees exhibit a higher sensitivity to clothianidin than larvae in toxicity studies (Supplemental Data, Figure S1). The adult bee counts on the 2 dates for the noncontrol simulations were each divided by the adult bee counts from the control simulations with corresponding scenario, site, and random seed. This percentage of adult bees in exposed versus control simulations was used as the response metric reflective of colony‐level effect.

$$Colony\;\;level\;\;effect\;=\frac{Number\;of\;adult\;bees\;\;in\;\;\exp osed\;\;\;colony}{Number\;of\;adult\;bees\;\;in\;\text{ control }\;\;colony}\times 100\;\;$$

#### Pollen foraging and colony‐level effects

As an indicator of in‐hive exposure to clothianidin, the amount of pollen collected from the central test cornfield was recorded. Pollen from the central test cornfield was only available for collection during corn tasseling (flowering), simulated between 22 July through 4 August and corresponding to days 203 through 216 in the landscape resource input file to BEEHAVE. For each simulation run, the percentage of pollen collected from the central test cornfield relative to all pollen collected during the same time period was calculated.

$$\begin{array}{l} Pollen\;collection\;from\;test\;corn\;field\\ \;\;\;\;\;\;\;\;\;\;\;\;\;\;\;=\frac{\sum_{i=203}^{216}Pollen\;collected\;from\;test\;corn\;field\;on\;day\;i}{\sum_{i=203}^{216}Pollen\;collected\;on\;day\;i}\times 100 \end{array}$$

Pollen collection from test cornfield was graphed against colony‐level effects.

#### Interaction with the landscape

**Figure 3:**
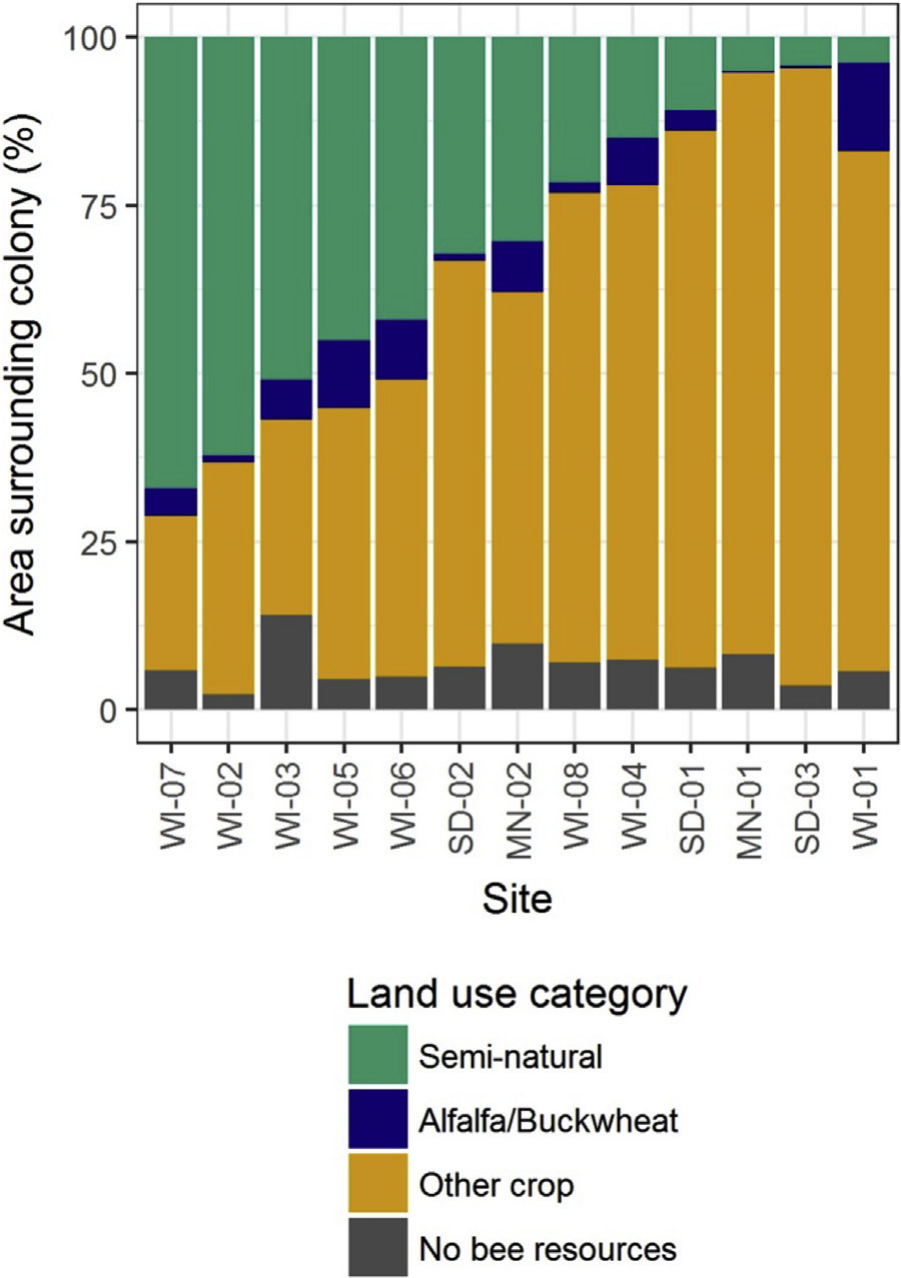
Percentages of land cover categories surrounding the 13 sites in the study. Sites are sorted from high to low semi‐natural land cover area. WI = Wisconsin; SD = South Dakota; MN = Minnesota.

Landscape compositions surrounding the 13 simulated colonies were quantified to explain variability in results from one site to the next. Land covers represented in the landscape resource input file as providing bee resources were grouped into 3 categories: alfalfa/buckwheat, other bee‐attractive crops, and semi‐natural land covers. Alfalfa and buckwheat are classified as highly attractive to honey bees with respect to both nectar and pollen, and were separated from other bee crops (dry beans and peas, corn, sorghum and millet, and soybeans) characterized as less attractive to honey bees (US Department of Agriculture 2015) for the analysis of landscape composition. (See Supplemental Data, Table S2 for classification of CDL land cover types.) For each land cover category, its percentage of the full area defined by a radius of 1.5 km around the hive was determined. In Figure 3, the percentages of the 3 land cover categories are shown for each site.

We compared the landscape composition with the percentage of corn pollen collected from the central test cornfield to examine whether bees collected more pollen from the test cornfield in some landscapes than in others. The collection of pollen from the central test cornfield was used as a proxy for exposure of the colony, and was analyzed in control simulations (without simulated residues) to avoid interactions of residue effects on foraging. Percentages of land cover categories (alfalfa/buckwheat, other crops, and semi‐natural land covers) present surrounding simulated colonies were graphed against percentages of pollen collected from test cornfields in control simulations.

Linear mixed‐effects regression models statistically tested these relationships while accounting for replicates within sites by setting site as the random effect variable (run with R lmerTest package; Kuznetsova et al. 2016). Two models were generated comparing percentage of pollen collected from test cornfields with: 1) percentage of other crop land cover (bee crops excluding alfalfa/buckwheat), and 2) percentage of semi‐natural land cover as the landscape composition fixed‐effects variable. Scenario (baseline or stress) was also tested, including as an interaction to test for variability in slope. In addition, test field size was evaluated as a check for potential confounding effects. All data and statistical analyses were completed in R software Ver 3.4.3 (R Development Core Team 2017) and plots were produced with the R ggplot2 package (Wickham 2009).

## RESULTS

### Colony dynamics

**Figure 4:**
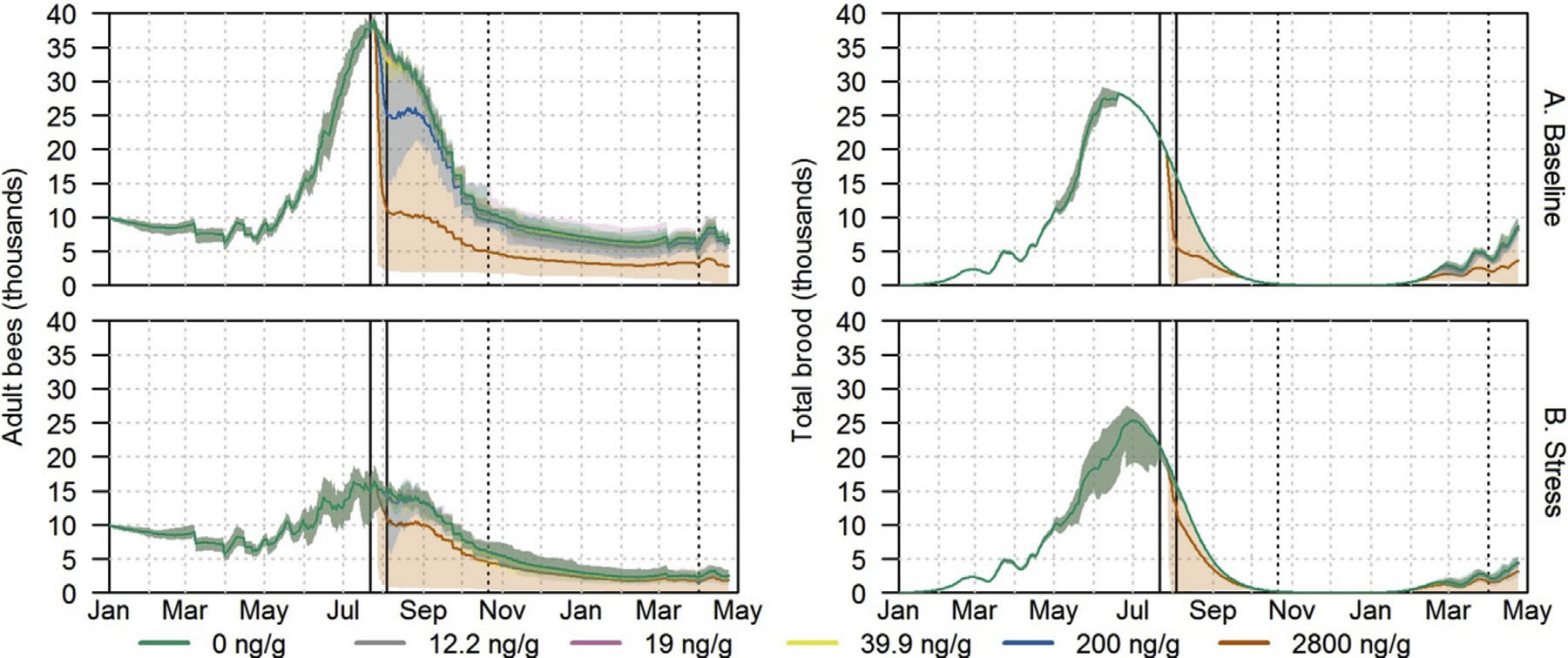
Colony dynamics covering the simulated time period from site WI‐08. (**A**) Baseline scenario. (**B**) Stress scenario. Number of adult worker bees and total number of brood are shown. Each line represents the average of 20 simulations colored by corn residue level; shaded areas indicate the range. Dates marked by solid vertical lines denote the beginning and end of corn tasseling. Dotted vertical lines correspond to the dates used for detailed analyses of adult bee numbers. WI = Wisconsin.

Daily numbers of adult worker bees and broods as well as total honey and pollen stores present in the hive were collected from the simulations with BEEHAVE. In Figure 4, an example of colony dynamics from simulations of site WI‐08 is shown covering the simulated 480 d. This site was chosen as an example because it was close to median among the 13 simulated sites in both landscape composition and colony‐level effects from simulated exposures. Colony dynamics differed between the baseline and stress scenarios (Figure 4A,B). In the stress scenario model runs, the number of adult bees and the size of honey and pollen stores had considerably lower maximum values than those in the baseline scenario. Brood numbers were also reduced.

In both scenarios, colony dynamics of controls and the 5 simulated residue levels were similar for all measures until onset of corn tasseling (22 July) when exposure via collection of pollen from the test cornfield began. At that time, the 2 highest residue levels became distinct from the controls. For the most extreme residue level simulated (2800 ng clothianidin/g pollen), the sharpest drop in adult bee numbers was observed. Adult bee numbers remained lower than in controls for the duration of the modeled period. The total brood numbers were also suppressed, and did not grow at the same rate as in controls during the spring after exposure (simulated year 2). Honey stores reached a lower maximum than in control, and less honey and pollen were consumed over time (Supplemental Data, Figure S4). For the simulated residue level of 200 ng clothianidin/g pollen, a decrease in adult bee numbers was also observed, but colonies recovered before the end of the foraging season (November). When a realistic maximum residue level of 39.9 ng clothianidin/g pollen (maximum corn pollen residue level measured in a recent Valent U.S.A. unpublished study with the soil‐applied clothianidin product Ampex insecticide) was simulated, adult bee numbers showed a small decrease compared with controls during August in the baseline scenario. For the residue levels of 19 and 12.2 ng clothianidin/g pollen (proposed residue thresholds at colony level for nectar and pollen/bee bread, respectively, in US Environmental Protection Agency [2017] preliminary pollinator risk assessment for clothianidin), no differences in colony dynamics compared with controls were found. Similar dynamics with varying magnitudes were observed in simulations of the other 12 sites (Supplemental Data, Figure S4).

### Colony‐level effects

**Figure 5:**
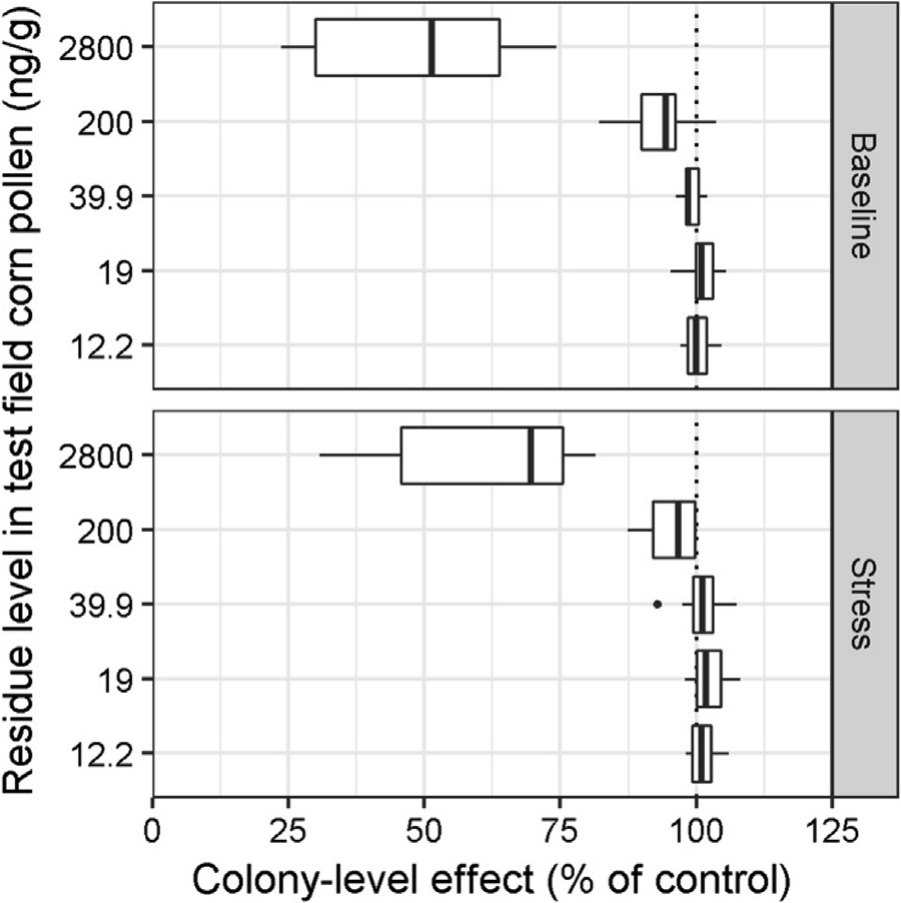
Colony‐level effects of simulated clothianidin residue levels in pollen from test cornfield. Percentage of adult bees in exposed compared to control hives on 21 October are shown. (**Top**) Baseline scenario. (**Bottom**) Stress scenario. Medians as percentage of control are indicated and boxes delineate the interquartile range between the 25th and 75th percentiles. Whiskers extend up to 1.5 times the interquartile range, and values beyond the whiskers are depicted as points.

Percentages of adult bees on 21 October (year 1) relative to control simulations were used as a measure of colony‐level effects. Across all sites, simulated control colonies had on average 10 732 adult bees in the baseline scenario (minimum and maximum adult bee numbers 7611 and 14 311, respectively), and 5598 in the stress scenario (minimum and maximum adult bee numbers 3222 and 7720, respectively). Simulations with a residue level of 2800 ng clothianidin/g pollen from test cornfields resulted in the greatest decrease in adult bee numbers compared with the control runs (Figure 5), with a wide range of responses among sites (Supplemental Data, Figure S6). Median colony‐level effects in the baseline scenario were more pronounced than in the stress scenario, in which control colonies had inherently lower values. Median adult bee numbers across sites were also reduced compared with controls in both scenarios if the pollen from the test cornfield was simulated with a residue level of 200 ng clothianidin/g pollen, although for some sites no effect was visible. Median adult bee numbers in simulated colonies with the 3 lowest and most realistic residue levels (12.2, 19, and 39.9 ng clothianidin/g pollen from the test cornfield) did not systematically differ from controls across sites. Colony‐level effects (as a percentage of adult bee numbers compared with controls) were also plotted for 1 April (year 2), representing colony strength in early spring when colonies start growing with similar outcomes after the overwintering period (Supplemental Data, Figure S5).

### Pollen foraging and colony‐level effects

**Figure 6:**
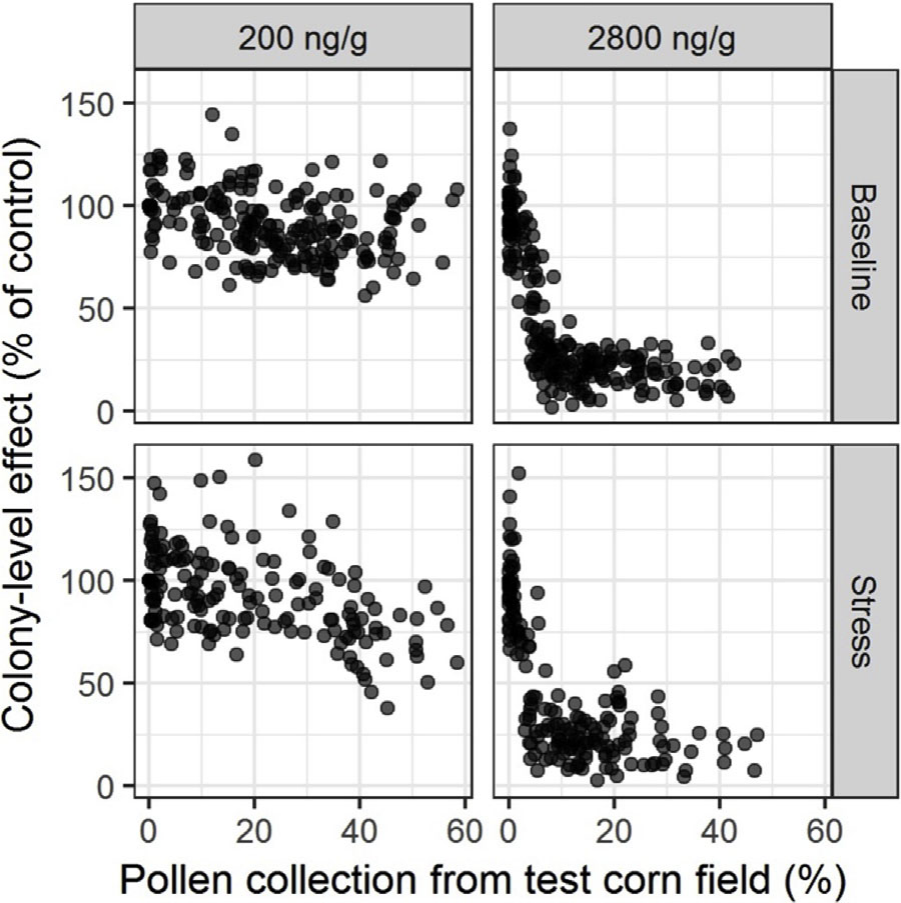
Relationship between percentage of pollen collected from test cornfield by each colony during corn tasseling (22 July–4 August) and colony‐level effects (percentage of adult bees compared to controls on 21 October). Each point represents the output from a single simulation run. (**Left**) Relationship shown for simulations with 200 ng clothianidin/g pollen from test cornfield. (**Right**) 2800 ng clothianidin/g pollen from test cornfield. (**Top**) Baseline scenario. (**Bottom**) Stress scenario.

Adult bee numbers in the simulated colonies varied among sites and repetitions even within the same residue level in pollen from the test cornfield, with the greatest variability occurring in the 2 highest simulated residue levels (200 and 2800 ng clothianidin/g pollen). To examine this variability among simulations, the colony‐level effects on 21 October were graphed against the percentage of pollen collected during corn tasseling. In Figure 6, outcomes from single simulation runs (20 per site), rather than averages across sites, are depicted. Colony‐level effects were related to the pollen collected from the test cornfield—the more pollen the simulated foragers gathered from the test cornfield compared with pollen collected from all patches during corn tasseling, the lower the number of adult bees in the colonies on 21 October. This relationship was more pronounced for the highest residue level simulated. Similar correlations were found with colony‐level effects from 1 April (year 2; Supplemental Data, Figure S7). The link suggested that the percentage of pollen the bees collected from the test cornfield was a measure representative of clothianidin exposure to the colonies.

### Interaction with the landscape

**Figure 7:**
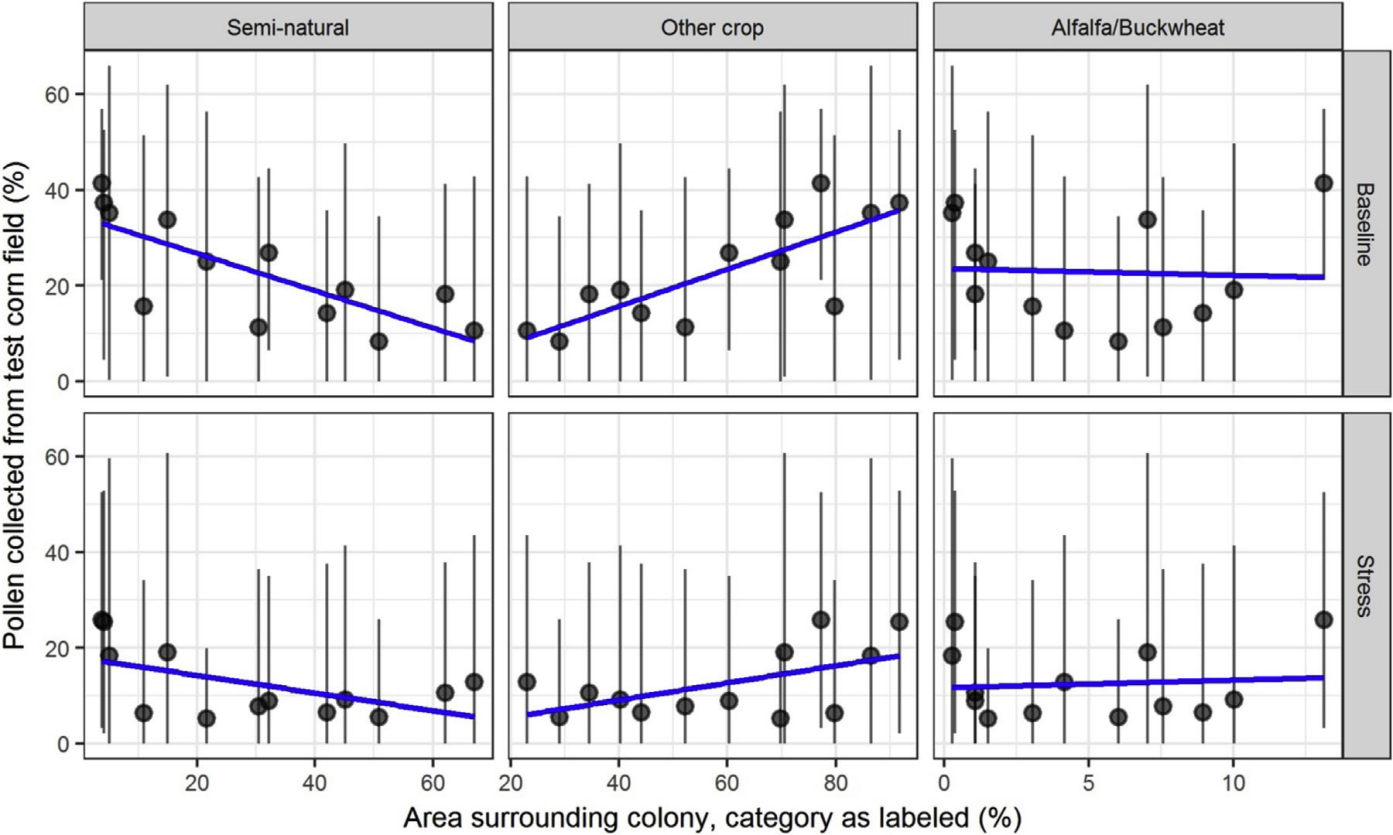
Impact of landscape composition on pollen collected from test cornfield in control simulations. Land cover types providing bee resources are categorized as percentage area (**left**) semi‐natural, (**middle**) other crop, and (**right**) alfalfa/buckwheat. Results are shown from (**top**) baseline scenario and (**bottom**) stress scenario. Points are averages per simulated site; whiskers are 90th percentile intervals by site; and blue lines denote linear trends.

Colony‐level effects from the 2 unrealistically high corn pollen residue levels simulated varied considerably among sites (Figure 5). Simulated sites differed mainly in landscape composition around the colony locations, implying that landscape structure may influence colony‐level effects via pollen collection. We analyzed the relationship between pollen collection from the test cornfield and the landscape composition at each site in control simulations only (Figure 7).

**Figure 8:**
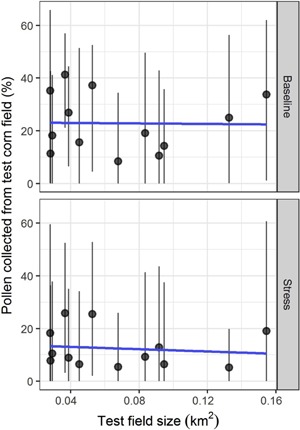
Scatter plots comparing the percentage of pollen collected from each site's test cornfield with the size of the test cornfields in both (**top**) baseline scenario and (**bottom**) stress scenario. Points are averages per simulated site; whiskers are 90th percentile intervals by site; and blue lines show linear trends. There was no correlation in either scenario (slope insignificantly different from 0; Pearson's correlation *p* values > 0.10).

Simulated colonies gathered more pollen from the test cornfields in sites with more bee‐attractive crops other than alfalfa or buckwheat (corn, soybeans, beans and peas, sorghum and millet, etc.). For the percentage area of semi‐natural land cover, the opposite was found: colonies collected less pollen from the test cornfields in sites with more semi‐natural land. Observed tendencies were stronger in the baseline scenario than in the stress scenario. The trends were quantified by applying linear mixed‐effects models (Table 3). An increase of 10% other crop land in a site and a complementary decrease of 10% semi‐natural land meant approximately 3.9% more pollen collected from the test cornfield on average in the baseline scenario (*p* = 0.00045 other crop; *p* = 0.00074 semi‐natural), and approximately 1.8% more pollen collected from the test cornfield on average in the stress scenario (*p* = 0.048 other crop; *p* = 0.050 semi‐natural). The percentage of alfalfa/buckwheat in a landscape was insignificant (*p* = 0.83), as visible in its flat slopes (Figure 7), and may be the result of its small range of values across sites (13% compared to 69% of other crop and 63% of semi‐natural land cover). Pollen gathered from test cornfields was not dependent on the size of the central test cornfield (Figure 8).

**Table 3:** Results for the 2 linear mixed effects models explaining pollen collection from the test cornfield, each with a different land cover category and the stress scenario as predictors

Model 1: % other crop as predictor				
Random effects	SD			
Site (13 total)	5.759			
Residual	16.743			
Fixed effects	Coefficient	SE	*df*	*p* value
Intercept	0.242	5.415	15.2	0.9649
% other crop	0.387	0.087	15.2	0.0005**
Stress scenario	1.717	4.174	505	0.6809
Other crop* stress	–0.208	0.067	505	0.0020*
Model 2: % semi‐natural as predictor				
Random effects	SD			
Site (13 total)	5.936			
Residual	16.759			
Fixed effects	Coefficient	SE	*df*	*p* value
Intercept	34.488	3.374	14.9	3.87 E‐8**
% semi‐natural	–0.388	0.092	14.9	0.0007**
Stress scenario	–16.533	2.547	505	2.05 E‐10**
Semi‐natural* stress	0.204	0.069	505	0.0034*

* Significant at <0.01.

** Significant at <0.001.

SD = standard deviation; SE = standard error; *df* = degrees of freedom.

## DISCUSSION

Mechanistic effects models are increasingly recognized as valuable tools to assess risks of pesticides to nontarget organisms and communities because they can combine information on the biology of the modeled system with exposure and effects, and judge risks on the level of organization of interest (Forbes et al. 2009; Raimondo et al. 2018). For the gauging of risks of pesticides to honey bees, potential effects on colonies are of interest; however, the mechanistic link among exposures, toxicological findings in laboratory tests on larvae and adult bees, and long‐term effects on colonies are not well understood (Henry et al. 2017; Sponsler and Johnson 2017). With the approach employed in the present study, we present exposure in realistic landscapes and link it to effects on broods and adult bees in colonies by applying the honey bee colony model BEEHAVE (Becher et al. 2014). We extended BEEHAVE with a pollen exposure‐effects module to allow for patch‐specific representation of residue levels of a pesticide in pollen (in the present case study, clothianidin in corn pollen), along with the other patch characteristics defining the landscapes surrounding the simulated colonies. The resulting colony‐level effects can be assessed over extended time periods. The extension and example application show how a honey bee colony model can be applied in pesticide risk assessment utilizing temporally and spatially explicit assumptions about sources of exposure in realistic landscapes.

Our results suggest that honey bee colonies exposed to a treated cornfield surrounding their colonies could be affected if residues in corn pollen from the field reach unrealistically high levels of 200 ng clothianidin/g pollen or higher. Nevertheless, no effects on bee numbers before overwintering (late October) or in the following spring (early April) were observed if simulated residue levels in pollen from the central cornfield were 39.9 ng clothianidin/g pollen or lower. The tested residue level of 39.9 ng clothianidin/g pollen was found to be the maximum residue level measured in an unpublished residue study conducted by Valent U.S.A. with corn treated with the soil‐applied clothianidin product Ampex insecticide (US Environmental Protection Agency # 59639‐EUP‐18). In a recent preliminary risk assessment, the following maximum concentrations of clothianidin in corn pollen were measured: 27.9 ng clothianidin/g pollen from soil‐treated corn (also with Ampex insecticide), and 23.8 ng clothianidin/g pollen from seed‐treated corn (US Environmental Protection Agency 2017). In the same risk assessment, a no‐observed‐adverse‐effect concentration of 19 ng clothianidin/g nectar and a lowest‐observed‐adverse‐effect concentration of 12.2 ng clothianidin/g pollen were proposed as protective levels for honey bee colonies. These recommended concentrations were derived from neonicotinoid residues measured in nectar and bee bread samples of colonies fed for 6 wk with neonicotinoid‐spiked sugar syrup containing 40 ng clothianidin/g feeding solution. Pollen brought into the hive by the bees did not have detectable neonicotinoid residues in the studies. Both indicated concentrations were also tested in the simulations applied in the present study, and did not result in effects on simulated colonies. Our results suggest that residue levels in pollen that might cause effects on honey bee colonies may not be easily derived from effect levels observed in studies where the exposure occurs via nectar, and could potentially lead to overly conservative exposure thresholds.

Colony‐level effects found in simulations with unrealistically high residue levels in corn pollen varied considerably across sites. The 13 simulated sites represented locations in the US Midwest with varying agricultural intensity (Figure 3). Sizes of the central treated cornfields varied among sites but were not correlated with colony‐level effects. However, the landscape composition of the simulated sites played a significant role in the size of colony‐level effects observed. Simulated colonies collected more pollen from sources other than the central cornfields if sites had greater coverage of semi‐natural land. Accordingly, in‐hive exposures of bees to clothianidin were not only dependent on residue levels found in pollen from a specific source (in this case corn) but also on the availability of pollen from other sources across the landscape. Landscape composition has been reported to be important for honey bee colony health and potential exposures to pesticides caused by foraging on treated crops. In a study conducted in North Dakota, USA, annual survival of honey bee colonies was strongly positively related to the area of uncultivated forage land around the colonies (Smart et al. 2016). Analyzing origins of honey‐bee collected pollen, Odoux et al. (2012) found diversity of pollen and landscape was correlated. Danner et al. (2014) assessed foraging by honey bee colonies next to cornfields by analyzing bee dances in the hive. These authors observed that pollen foragers collected pollen from other sources at significantly farther distances than the cornfields, leading them to suggest that alternative floral resources present in the landscape may lead to reduced pollen collection from corn. This is consistent with the US Department of Agriculture (2015) considering corn as a low‐attractiveness (wind‐pollinated, attractive under certain conditions) crop to bees.

The resolution of land cover data used for input to BEEHAVE (30 m × 30 m grid) does not capture all variability in resource availability surrounding a hive. For instance, floral resources in roadsides and utility rights‐of‐way have been identified as potentially beneficial for pollinators (Russell et al. 2005; Morandin and Kremen 2013) but may not be captured in the landscape input files. Nectar and pollen resource availabilities from the represented land cover patches were estimated based on reported flower‐level measurements and categorizations of their attractiveness to honey bees. Nevertheless, BEEHAVE provides a tool to evaluate the dependence of nectar and pollen collection from different sources in varying landscape contexts. In‐hive exposures and ultimately colony‐level effects do not only depend on residue levels in bee resources but also on other bee resources across the landscape, a dependency also observed in empirical studies with honey bee colonies.

The BEEHAVE model has previously been applied to assess impacts on long‐term colony health from temporally restricted adult bee and larval mortality to simulate lethal effects of pesticides, as well as reduced forager efficiency to mimic sublethal effects (Rumkee et al. 2015; Thorbek et al. 2017a, 2017b). The results of these studies suggest that colony‐level impacts of increased worker or brood mortalities as well as reduced foraging efficiency are dependent on timing of the applied stressor and the colony condition in the absence of the stressor. Henry et al. (2012) utilized another model of honey bee colony dynamics based on simple, linked differential equations to judge colony‐level impacts of a pesticide. Exposures were simulated as the percentage of foragers experiencing effects (increased mortality) over a given time period. Kuan et al. (2018) presented a model (VarroaPop + pesticide) extending the VarroaPop model of in‐hive colony dynamics including varroa mite reproduction (DeGrandi‐Hoffman and Curry 2004) with pesticide exposure and effects. They included a detailed submodel simulating residues in nectar and pollen from different types of application of pesticides, and applying the residue levels to nectar and pollen in the hive when foraging occurs during the exposure period. This corresponds to the overly conservative assumption that pesticide applications are uniformly distributed to the entire landscape around the hives.

Mechanistic models can inform pesticide risk assessment data because they can take into account different exposure pathways (e.g., contact exposure or exposure via consumption of nectar or pollen) and results within the colony (e.g., direct mortality of adult bees and larvae mediated by stage‐specific ingestion rates or sublethal effects) causing reduced foraging efficiency or queen productivity. In the present approach, we simulate exposure via residues in pollen from a single, treated field in a realistic landscape context. Effects occur as the result of consumption of pollen, whereby bee stage‐specific consumption rates and dose responses are measured.

Given that clothianidin is only applied in corn via soil application or seed treatment—and that corn only produces pollen—in the present case study the pollen exposure‐effects module used in BEEHAVE does not address exposures through nectar or other routes such as direct spray. The simulation results presented assume a single, treated field as the closest resource patch to the colony. However, because of the patch‐ and day‐specific representation of bee resources in the landscape, multiple exposures from various sources (e.g., diverse crops) at different times of the year could be simulated with the extended version of BEEHAVE. For the present case study of corn treated with clothianidin via soil application and/or treated seeds, the extended model gave an estimate of colony‐level risk, taking into account realistic landscape‐level and in‐hive exposures and the impacts on colony dynamics over time. Sizes of simulated colonies before and after the overwintering period (October of year 1; April of year 2) were compared with control colonies, capturing long‐term, colony‐level effects of clothianidin bee exposure during corn flowering between late July and early August.

Baseline and stress scenarios of conditions encountered by the colonies were simulated. Effects of pesticides could potentially interact with other stressors affecting colonies, leading to the suggestion that stressed colonies would provide a more conservative setting for pesticide risk assessments (Thorbek et al. 2017a). The results from the present study indicate that proportional effects on colony size from pesticides may be more pronounced in strong colonies (baseline scenario).

In the present study, we did not consider potential sublethal effects from exposure to the neonicotinoid. In laboratory studies, effects of neonicotinoids on development time, proboscis extension reflex, locomotor activity, and memory in bees were observed, and orientation and activity of foragers may be affected. Concentrations causing sublethal effects fall into a range comparable with lethal effects (Blacquière et al. 2012). The assessment of sublethal effects in standard laboratory toxicity studies with honey bees was included in the most recent test guidelines (US Environmental Protection Agency 2016b). Nonetheless, it is unclear how the sublethal endpoints of abnormal behavior or movement relate to behaviors important for colony success such as brood care and foraging. Thorbek et al. (2017b) assessed the sensitivity of BEEHAVE to increased foraging times and decreased brood care as behaviors that could be implicated in sublethal effects. Dose–response relationships for behavioral processes linked to colony performance would need to be available for the inclusion in realistic exposure‐effects evaluations with a honey bee colony model. For the consistent and efficient application of a colony model such as BEEHAVE in pesticide risk assessment, model parameterizations and simulation scenarios that could be applied across diverse measurements would have to be developed. Standard scenarios could be established representing different geographical and climatic regions, landscape compositions, and hive‐management activities. In addition, a colony‐level endpoint of acceptable effect size would need to be defined.

## CONCLUSIONS

Honey bee colony models have been identified as tools that could potentially be useful in pesticide risk assessments (European Food Safety Authority 2016; Sponsler and Johnson 2017; Kuan et al. 2018). BEEHAVE was developed including both in‐hive colony dynamics and interactions with the landscape through resources. By extending BEEHAVE with a pollen exposure‐effects module, we made use of BEEHAVE's full capacity to depict bee resources and potential exposures related to them. This was done in a temporally and spatially explicit landscape structure of real, treated cornfields in representative corn‐growing areas in the US Midwest. Colony‐level effects from a treated cornfield as the only source of clothianidin exposure could only be observed in the simulations with unrealistically high residue levels in the pollen. However, no colony effects were observed when simulating the 3 most realistic residue levels (≤39.9 ng clothianidin/g pollen) irrespective of simulated background colony condition. The landscape composition affected the simulated colonies via collection of pollen from the treated field. The more semi‐natural land cover surrounding a simulated location—the less pollen collected from the central field.

The presented pollen exposure‐effects module shows that the extension of BEEHAVE to include detailed exposure and effects assumptions makes the model a potentially useful mechanistic tool applicable in pesticide risk assessment. Additional exposure routes including nectar, water, and direct exposure of foragers, as well as sublethal effects, would need to be considered. Calibration and validation of the model with data from honey bee colony field research would increase the reliability in applications.

## Supplemental Data

The Supplemental Data are available on the Wiley Online Library at DOI: 10.1002/etc.4314.

## Supporting information

This article includes online‐only Supplemental Data.

Supporting Data S1.

Supporting Data S2.

## Data Availability

The extended BEEHAVE model code is provided in the Supplemental Data.
