# Supplementary material for: Honey bee colony‐level exposure and effects in realistic landscapes: An application of BEEHAVE simulating clothianidin residues in corn pollen
Source: Environ Toxicol Chem. 2019 Jan 7;38(2):423–35. doi: 10.1002/etc.4314 (PMC6850421; doi:10.1002/etc.4314)
Supplement: Supplementary file 1 — Supporting Data S1. [file ETC-38-423-s001.pdf]

Honey bee colony-level exposure and effects in realistic landscapes: an application of BEEHAVE simulating clothianidin residues in corn pollen

TRACE Documentation

Transparent and comprehensive ecological model documentation

(Schmolke et al. 2010; Grimm et al. 2014)

Table of Contents

1. PROBLEM FORMULATION 3
2. MODEL DESCRIPTION 3
2.1. Summary of pollen exposure-effects module 3
2.2. Implementation of the pollen exposure-effects module in BEEHAVE 3
2.2.1. New model variables 4
2.2.2. New model parameters (on interface) 4
2.2.3. New and changed model procedures 5
2.2.4. Implementation of pollen collection 6
2.2.5. Implementation of pollen storage (in daily cohorts) 7
2.2.6. Implementation of pollen consumption 7
2.2.7. Implementation of pesticide effects 7
3. DATA EVALUATION FOR MODEL CONCEPTUALIZATION AND PARAMETERIZATION 8
3.1. Dose-response relationships from laboratory toxicity studies 8
3.2. Landscapes represented in the simulations 11
3.3. Methods for estimating bee resources from the landscape 18
3.3.1. Estimation of bee resources from crops 26
3.3.2. Bee resource specifications for crops 27
3.3.3. Estimation of bee resources from semi-natural land covers 33
4. CONCEPTUAL MODEL EVALUATION 36
5. IMPLEMENTATION VERIFICATION 36
5.1. Correspondence of model outputs with BEEHAVE_BeeMapp2015 36
5.2. Transfer of residues in landscape patches to stores and cohort exposures 36

5.3. Dose-response implementations ... 36
6. MODEL OUTPUT VERIFICATION ... 36
7. MODEL ANALYSIS ... 37
7.1. Simulation scenarios ... 37
7.1.1. Parameter settings ... 37
7.1.2. Production of scenario-, site- and pollen residue-level specific input files ... 39
7.2. Results ... 40
7.2.1. Colony dynamics ... 40
7.2.2. Colony-level effects ... 45
7.2.3. Pollen foraging and colony-level effects ... 47
8. MODEL OUTPUT CORROBORATION ... 47
References ... 48

1. PROBLEM FORMULATION

The BEEHAVE model is a mechanistic model of a honey bee colony, representing in-hive colony dynamics, mite infestations and foraging. BEEHAVE has been identified as potentially valuable tool for colony-level pesticide risk assessment. However, exposures to pesticides and their effects on bees and brood are not implemented in the model.

With the pollen exposure-effects module developed for the current paper, we demonstrate how exposure and effects can be implemented explicitly in BEEHAVE. We focus on a single exposure pathway (pollen collection and consumption) to assess potential colony-level effects of exposure to pesticide-treated corn fields in a landscape context.

2. MODEL DESCRIPTION

A model description following the ODD (Overview, Design, and Details) protocol, a standardized protocol for the description of individual-based models (Grimm et al. 2006, 2010) is available for BEEHAVE (http://beehave-model.net).

For the current project, we extended the BEEHAVE model to represent exposure via pollen, and effects from consumption of exposed pollen in the colony. For the pollen exposure-effects module (PEEM), variables and procedures were added to the model. A comprehensive list of additions and changes to the BEEHAVE model are provided in this section. The changes were applied to the BEEHAVE version "BEEHAVE_BeeMapp2015".

2.1. Summary of pollen exposure-effects module

Pesticide concentrations in pollen are included in the landscape input file to BEEHAVE (INPUT_FILE), i.e., concentrations in ng a.i./g pollen are defined as new column in the file for each bee resource patch and day. Pollen loads collected by pollen foragers are assigned with the patch-specific pesticide concentration. In the hive, pollen loads are added to the pollen stores. Pollen stores are represented in age-specific cohorts, i.e., pollen collected on the same day is added to the same cohort. From the residue concentrations in the pollen loads added to a single cohort, the average concentration is calculated. No pollen is added to pollen cohorts 1 day and older, but pollen is consumed by adult bees and larvae. For consumption, younger pollen is consumed first.

Each larva and adult bee cohort stores the amount of pesticide consumed with the pollen for each day of its developmental stage. In adult bee cohorts, a dose-response function is applied according to the sum of pesticide amount consumed in the past 2 days and past 10 days, mirroring the durations of standard toxicity tests for acute and chronic toxicity (see section 3.1. for more detail on the use of data from standard toxicity studies). For larva cohorts, a dose-response function is applied at the end of the larval stage. The dose-response functions define the proportion of bees or larvae dying due to the consumed pesticide, and cohort numbers are reduced accordingly.

2.2. Implementation of the pollen exposure-effects module in BEEHAVE

The new version of BEEHAVE including the pollen exposure-effects module (PEEM) is called 'BEEHAVE_BeeMapp2015_PEEM'. In the following, we describe changes and additions to the model code in detail. In the model code, additions and changes are marked as follows:

NEW FOR BEEHAVE_BEEMAPP2015_PEEM

2.2.1. New model variables

<u>Global variables ('global'):</u>

NewForagerExposureHistory

PollenPesticideConcentrationList

PollenStoreByAgeList

Pollen stored in the hive is represented in the list 'PollenStoreByAgeList.' The list contains 9 items ('days'), representing pollen ages as follows:

Day 0 – pollen collection, i.e. newly collected pollen is added to this item each day; no consumption

Day 1 – stored pollen, consumption by bees

Day 2 - stored pollen, consumption by bees

⋮

Day 8 – 'old' pollen: all pollen 8 days and older is combined in this item, and consumed with lowest priority, i.e. after all other pollen is empty (days 1-7; corresponds to 'class 6' of beebread in EFSA (2016) conceptual model)

In parallel, the residue concentration in the pollen is stored in a separate list, 'PollenPesticideConcentrationList.' Each item (day) in the list represents the residue concentration in the corresponding stored pollen. Note that the residue level does not change after day 0, i.e. each item is propagated to the next item/day after each BEEHAVE time step (for details, see section 'Pollen storage').

<u>Bee cohort variables ('turtles-own' in NetLogo code; applies to all 'cohorts' and 'foragerSquadrons'):</u>

exposureHistory – list that stores the daily exposure to the pesticide (clothianidin) in ng per bee.

<u>Forager variables ('foragerSquadrons-own'):</u>

pesticideInCollectedPollen – stores the residue level in pollen collected by the forager squadron in ng a.i./g pollen.

<u>Resource patch variables ('flowerPatches-own'):</u>

pollenPesticideConcentration_ng_per_g – stores the pesticide concentration in pollen of a resource patch in ng a.i./g pollen. This value is read in from the landscape resource INPUT file.

2.2.2. New model parameters (on interface)

The following parameters were included on the interface, i.e. values defined at setup of each model run. The parameters defining pollen consumption were set in BEEHAVE_BeeMapp2015 in 'PollenConsumptionProc' (i.e. the variables were present in the BEEHAVE_BeeMapp2015 version, but were not interface parameters).

1. <u>Parameters determining pollen consumption</u> (parameter values used in BEEHAVE_BeeMapp2015 are given):
    a. *DAILY_POLLEN_NEED_FORAGER* (1.5 mg/day)
    b. *DAILY_POLLEN_NEED_IHBEE* (1.5 mg/day)

Note: in BEEHAVE_BeeMapp, only one variable was used for the two above, ‘DAILY_POLLEN_NEED_ADULT.’

c. *DAILY_POLLEN_NEED_ADULT_DRONE* (2 mg/day)
d. *DAILY_POLLEN_NEED_LARVA* ([142 / (PUPATION_AGE - HATCHING_AGE)] mg/day; or 23.6 mg/day)
e. *DAILY_POLLEN_NEED_DRONE_LARVA* (50 mg/day)

2. Parameter determining pollen consumption preference: *PollenConsumptionPreference*: “FreshToOld” sets consumption order starting with pollen 1 day old, and proceeding to older pollen; “Random” sets consumption to randomly chosen from all pollen age classes.
3. Parameters determining clothianidin dose responses: adult dose responses (acute and chronic) are represented by a Weibull function, larval dose responses by a log-logistic function (see details in section ‘Pesticide effects’):
    a. *AdultAcuteSlope*
    b. *AdultAcutePower*
    c. *AdultChronicSlope*
    d. *AdultChronicPower*
    e. *LarvaAcuteIntercept*
    f. *LarvaAcuteSlope*
    g. *LarvaChronicIntercept*
    h. *LarvaChronicSlope*
4. The switch on the interface 'PollenIdeal' was removed for version BEEHAVE_BeeMapp2015_PEEM, and no constant ideal pollen storage in the hive can be set.

2.2.3. New and changed model procedures

CreateFlowerPatchesProc – changed procedure: ‘pollenPesticideConcentration_ng_per_g’ is set to value given in INPUT file (item 15, or last row). Note that no pesticide concentrations can be applied if default flower patches are used, i.e. pesticide concentration is currently only implemented with the use of a landscape resource INPUT_FILE.

DailyUpdateProc – changed procedure: ‘aging’ of pollen added in this procedure (see details in section 2, ‘Pollen storage’); pesticide concentration in pollen is read in from input file:
set pollenPesticideConcentration_ng_per_g item 15 TodaysSinglePatchList

DoseResponseAdultsProc - new procedure: implements Weibull function, and returns number of dead bees.

DoseResponseLarvaeProc - new procedure: implements log-logistic function, and returns number of dead larvae.

DroneEggLayingProc - changed procedure: includes set up of empty list ‘exposureHistory.’

DroneLarvaeDevProc - changed procedure: procedures ‘PesticideAcuteEffectsDroneLarvaeProc’ and ‘PesticideChronicEffectsDroneLarvaeProc’ are called, and the returned number of larvae that died are added to ‘numberDied’ (see section 4. ‘Effects’ for details).

EatPollen – new procedure: stored pollen is consumed either in order of pollen age (fresh pollen is consumed first) or in random order. The procedure includes the exposure to the pesticide by adding to ‘exposureHistory.’ For details, see section 3. ‘Consumption.’

Foraging_collectNectarPollenProc – changed procedure: added pesticide concentration in collected pollen.

Foraging_unloadingProc – changed procedure: new pollen collected by foragers is added to item 0 of 'PollenStoreByAgeList;' pesticide concentration is averaged across all pollen collected during the same day (see section 1. 'Pollen collection' for details).

NewDroneLarvaeProc - changed procedure: includes set up of empty list 'exposureHistory.'

NewDronesProc - changed procedure: includes set up of empty list 'exposureHistory.'

NewDronePupaeProc - changed procedure: includes set up of empty list 'exposureHistory.'

NewForagersProc – changed procedure: initialization of exposure variables included; exposure history from in-hive bees is transferred to new foragers using the variable 'NewForagerExposureHistory.'

NewIHbeesProc - changed procedure: includes set up of empty list 'exposureHistory.'

NewWorkerLarvaeProc - changed procedure: includes set up of empty list 'exposureHistory.'

NewWorkerPupaeProc - changed procedure: includes set up of empty list 'exposureHistory.'

PesticideAcuteEffectsAdultsProc – new procedure: applies acute pesticide effects to adult bees.

PesticideAcuteEffectsDroneLarvaeProc – new procedure: applies acute pesticide effects to drone larvae.

PesticideAcuteEffectsLarvaeProc – new procedure: applies acute pesticide effects to larvae.

PesticideChronicEffectsAdultsProc – new procedure: applies chronic pesticide effects to adult bees.

PesticideChronicEffectsDroneLarvaeProc – new procedure: applies chronic pesticide effects to drone larvae.

PesticideChronicEffectsLarvaeProc – new procedure: applies chronic pesticide effects to larvae.

PollenConsumptionProc – changed procedure: adult bees consume pollen first, then larvae. Each bee cohort calls 'EatBeebread.'

ReadFileProc – changed procedure: 16 columns (instead of 15 columns in BEEHAVE_BeeMapp2015) are read from the input file ('INPUT_FILE') as given on the interface.

WorkerEggLayingProc - changed procedure: includes set up of empty list 'exposureHistory.'

WorkerIHbeesDevProc – changed procedure: 'exposureHistory' of new foragers is stored as 'NewForagerExposureHistory' to assure continuous exposure memory when IH bees become foragers.

WorkerLarvaeDevProc – changed procedure: procedures 'PesticideAcuteEffectsLarvaeProc' and 'PesticideChronicEffectsLarvaeProc' are called, and the returned number of larvae that died are added to 'numberDied' (see section 4. 'Pesticide effects' for details).

2.2.4. Implementation of pollen collection

The column "pollenPesticideConcentration" was added to the input file ('INPUT_FILE'): column 16 now contains the daily pesticide (clothianidin) concentration in pollen for each flower patch in the landscape. Residue concentrations are given in ng a.i./g pollen. Input files to be used with BEEHAVE_BeeMapp2015_PEEM have to be prepared accordingly. The additional column is read in with the other columns in the procedure 'ReadFileProc.'

The ‘INPUT_FILE’ is read to a list called ‘AllDaysAllPatchesList.’ This list is then called in ‘DailyUpdateProc,’ and the values are assigned daily to the variables of each flower patch. The pesticide concentration from the input file is stored in the variable ‘pollenPesticideConcentration’ for each flower patch.

When foragers collect pollen, the pesticide concentration is “carried” with the pollen they collect (procedure ‘Foraging_collectNectarPollenProc’). In BEEHAVE, pollen foragers are either simulated to be successful, bringing back a full ‘POLLENLOAD’ of 15 µg, or unsuccessful bringing no pollen (and no residue) back to the colony. Each new pollen load brought to the hive by a forager squadron is added to the pollen store of day 0. The pesticide concentration of each new pollen load brought into the hive changes the pesticide concentration of the pollen store of day 0 considering the mass of both. The addition of pollen loads to the pollen store of age 0 days, and the calculation of the average pesticide concentration, are implemented in the procedure ‘Foraging_unloadingProc’.

2.2.5. Implementation of pollen storage (in daily cohorts)

At the end of each day, pollen stores get reassigned according to their age (in days), i.e. in the list ‘PollenStoreByAgeList,’ pollen from day 0 is reassigned to day 1, etc. The pesticide concentration is propagated through the list ‘PollenPesticideConcentrationList’ in the same way. The aging of pollen stores is implemented in the procedure ‘DailyUpdateProc’.

2.2.6. Implementation of pollen consumption

Pollen consumption is implemented in the new procedure ‘PollenConsumptionProc.’ Cohorts are called in random order to consume pollen. Each cohort/squadron calls procedure ‘EatPollen’ to consume pollen starting with pollen 1 day old and proceeding to the oldest stored pollen as applicable. Consumption occurs at the rate defined by the developmental stage of the bee (larva, in-hive bee or forager).

In-hive bees (nurses) can keep/restore their protein stores if stored pollen is still available in the hive after consumption. Otherwise, pollen shortage is assumed which may lead to larval starvation if it continues, i.e. if protein stores in in-hive bees cannot be replenished. The protein stores of in-hive bees are simulated in ‘PollenConsumptionProc,’ and are represented by the variable ‘ProteinFactorNurses.’ Note that the protein stores of in-hive bees can only be replenished from stored pollen (pollen one day and older). Protein stores of in-hive bees define the protein status of the colony, but consumption is implemented as directly occurring from the pollen stores for both larvae and adult bees. If the protein stores of nurse bees are depleted, egg and larva deaths are simulated.

2.2.7. Implementation of pesticide effects

Pesticide effects (acute and chronic) are applied to adult bees (workers and drones) in the procedure ‘DailyUpdateProc’ by calling the effects procedures (see below). The same cohort of bees experiences both acute and chronic effects if parameters for both are set on the interface. Acute effects are applied first. Effects can be turned off by setting both variables applying to each dose-response function to zero on the interface (e.g., setting AdultAcuteSlope = 0 *and* AdultAcutePower = 0 results in no adult acute response to pesticide exposure).

Effects on larvae are applied in ‘WorkerLarvaeDevProc’ and ‘DroneLarvaeDevProc’ for worker and drone larvae, respectively. Larval chronic toxicity is applied at time of pupation.

The effects procedures are implemented according to bee developmental stage (larva/adult) and considered exposure duration (acute/chronic): ‘PesticideAcuteEffectsAdultsProc,’ ‘PesticideChronicEffectsAdultsProc,’ ‘PesticideAcuteEffectsLarvaeProc,’ ‘PesticideChronicEffectsLarvaeProc.’ For acute effects, the last two entries in the list ‘exposureHistory’

are added, and used as the exposure value. Note that data for acute toxicity in larvae are currently not available. The procedure was included to allow the inclusion of acute effects on larvae once data from such studies are available. For chronic effects, the last 10 entries in the list 'exposureHistory' are added, and used as the exposure value for adults. In larvae, all entries in the list are added. Effects are applied to bee cohorts by calculating the proportion of bees dying due to the exposure according to the dose-response function.

For adult acute and chronic toxicity, a Weibull function is implemented for the dose-response functions because it provided the best fit for the data from the acute and chronic toxicity studies conducted with clothianidin (see Section 1.2.). Note that no background mortality was assumed for dose-response functions as background mortality (in unexposed bees) is occurring in BEEHAVE outside of the pesticide module. Adult dose-responses are applied to workers and drones because only data on worker bees are available.

For larval chronic toxicity, a log-logistic function is implemented as the dose-response function (see Section 1.2.). Data are currently only available from larval chronic toxicity studies. Note that no background mortality was assumed for dose-response functions as background mortality (in unexposed bees) is occurring in BEEHAVE outside of the pesticide module. Dose-responses are applied to worker and drone larvae because only data for worker larvae were available.

3. DATA EVALUATION FOR MODEL CONCEPTUALIZATION AND PARAMETERIZATION

In this section, the data sets used in model parameterization and as inputs to the model are described. The description is limited to the data used for the pesticide exposure-effects module and the site-specific simulations applied in the current paper. Data sources used for the development and parameterization of the BEEHAVE model were described by Becher et al. (2014).

3.1. Dose-response relationships from laboratory toxicity studies

Data from three laboratory toxicity studies were available to estimate lethal effects of clothianidin consumption on larvae and adult honey bees. The studies were conducted according to standard test guidelines for assessment of pesticide risks to bees (USEPA 2014). In the larval chronic study, honey bee worker larvae (24-48 hours after hatching) were transferred to artificial cells in the laboratory and provided with larval diet containing either no pesticide (control) or a given concentration of clothianidin. Diet with the same concentration of clothianidin was provided until pupation (5-8 days after hatching). The actual mass of compound (clothianidin) consumed by larvae was not measured, but the concentration in the feeding solution was measured.

From the study authors, we received the estimate of 320 µl feeding solution consumed per larva over the exposure period (which corresponds to the whole larval stage). This estimate is in line with larval consumption rates reported in Sabatini et al. (2009), and was used to estimate the amount of compound consumed by the larvae in the study.

The feeding solution is a sugar solution, and thus, has a higher density than water[1]. We account for the solution density by only considering the sugar content (not the content of other components like protein)

[1] http://www.lclane.net/text/sucrose.html

to estimate the density. The sugar content of the feeding solution falls between 15.5 and 21%. Using the average, we estimate the density of the solution of 1.07 g/ml.

The amount of clothianidin consumed by each larva can then be estimated using the following equation:

$$\frac{x\ \mu g\ a.i.}{g\ solution} \times \frac{1.07\ g\ solution}{ml\ solution} \times \frac{320\ \mu l}{larva} \times \frac{1\ ml}{10^3 \mu l} = m\ \frac{\mu g}{larva} \qquad \text{(S1)}$$

where

x: concentration of clothianidin in the feeding solution (µg/g)

m: mass of clothianidin consumed by the larva (µg)

The mass m of clothianidin consumed per bee larva is then used as measure of dose for the function fit with Benchmark Dose Software (BMDS; USEPA 2016). Proportion of adult bees emerging from pupal stage was measured as the survival endpoint and compared to controls whose larval diet did not contain the pesticide.

For adult honey bees, data from two studies were available: a 48-hour acute study and a 10-day chronic study. In both designs, honey bee workers were collected from a hive and transferred to the laboratory. Bees were fed *ad libitum* with sucrose solution (500 g/l) for the duration of the study. Sucrose solutions with a range of pesticide concentrations were fed to the treatment groups in the studies. Actual volume of sucrose solution consumed by bees in the study, and thus, amount of clothianidin consumed, was reported in the adult studies. Proportion of surviving bees in each treatment group was compared to controls for the assessment of effects.

We used the software BMDS (USEPA 2016) to fit dose-response functions to the data available for the three study designs conducted to assess toxicity of clothianidin to honey bees. Dose was defined as the total amount of clothianidin consumed across the exposure period of the study. Effects were defined as fraction of bees dead at the end of the exposure period for the adult studies and at time of emergence from pupal stage for the larval chronic study. In BMDS, a range of functions are fit to a given data set and the goodness of fit is compared. Background mortalities in control groups are accounted during function fitting. The log-logistic function provided the best fit to the larval chronic study data:

$$\mathrm{f(m)} = 1 \,/\, \left(1 + \mathrm{e}^{(-\mathrm{h} - \mathrm{s}\ln(\mathrm{m}))}\right) \qquad \text{(S2)}$$

where
m: amount compound consumed per larva (µg a.i./larva)
h: intercept
s: slope

A Weibull function provided the best fit to the data from the adult acute and chronic studies:

$$\mathrm{f(m)} = 1 - \mathrm{e}^{(-\mathrm{s}\,\mathrm{m}^{\mathrm{p}})} \qquad \text{(S3)}$$

where
m: amount compound consumed per larva (µg a.i./larva)
s: slope
P: power

Parameters fitted to study data and applied to dose-response functions implemented in BEEHAVE are listed in Table S1.

Table S1. Fitted parameters of dose-response functions for larval chronic, adult acute and adult chronic toxicity of clothianidin as applied in BEEHAVE.

	Larval chronic		Adult acute	Adult chronic
**Function**	Log-logistic (Equation S2)	**Function**	Weibull (Equation S3)	
**Intercept**	0.073	**Slope**	2481.06	207.17
**Slope**	1.042	**Power**	1.510	1.514

In BEEHAVE, we implemented the dose-responses accordingly, using the log-logistic function defined by the intercept and slope as model input parameters for effects on larvae and the Weibull function defined by slope and power as model input parameters for effects on adults. Background effects on controls, present in the larval chronic and adult chronic studies, were not accounted for since the curves are applied to a model with no effects on controls, and background mortality of unexposed larvae and adult bees is simulated in the model. The resulting dose-response relationships for clothianidin are shown in Fig. S1.

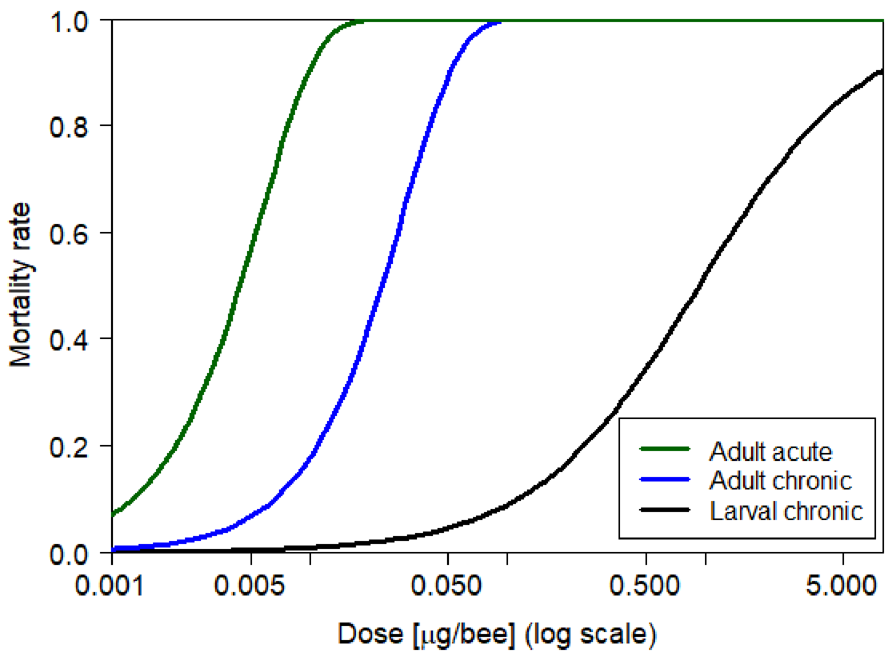

Fig. S1. Dose-response relationships derived from data from larval chronic, adult acute and adult chronic toxicity studies for clothianidin. Dose is defined as total amount of clothianidin consumed per larva or adult bee during the exposure period. Effects are expressed as fraction bees alive at the end of the exposure period for the adult studies and emergence from pupal stage for the larval chronic study. Data from the larval chronic study are fit using the log-logistic function, and data from the adult acute and chronic studies are fit using the Weibull function.

3.2. Landscapes represented in the simulations

In Fig. S2, the land cover maps from Cropland Data Layer (CDL) for the 13 sites included in the study are presented. The simulated colony location is shown in the center of the maps, and corresponds to the centroid of the central test corn field. A radius of 1.5 km around the central location is represented. Landscape resource input files to BEEHAVE were derived from these maps. Adjacent pixels in CDL maps with identical land covers were combined to patches. Distance between patches and the simulated central colony location was calculated from the centroid of each patch.

Fig. S2 (following pages). Landcover maps for the sites simulated in the current study. The central red point marks the simulated location of the colony surrounded by the central test corn field (delimited by green dots and red line. Small blue dots show the centroids of homogeneous patches in the landscape.

MN-01

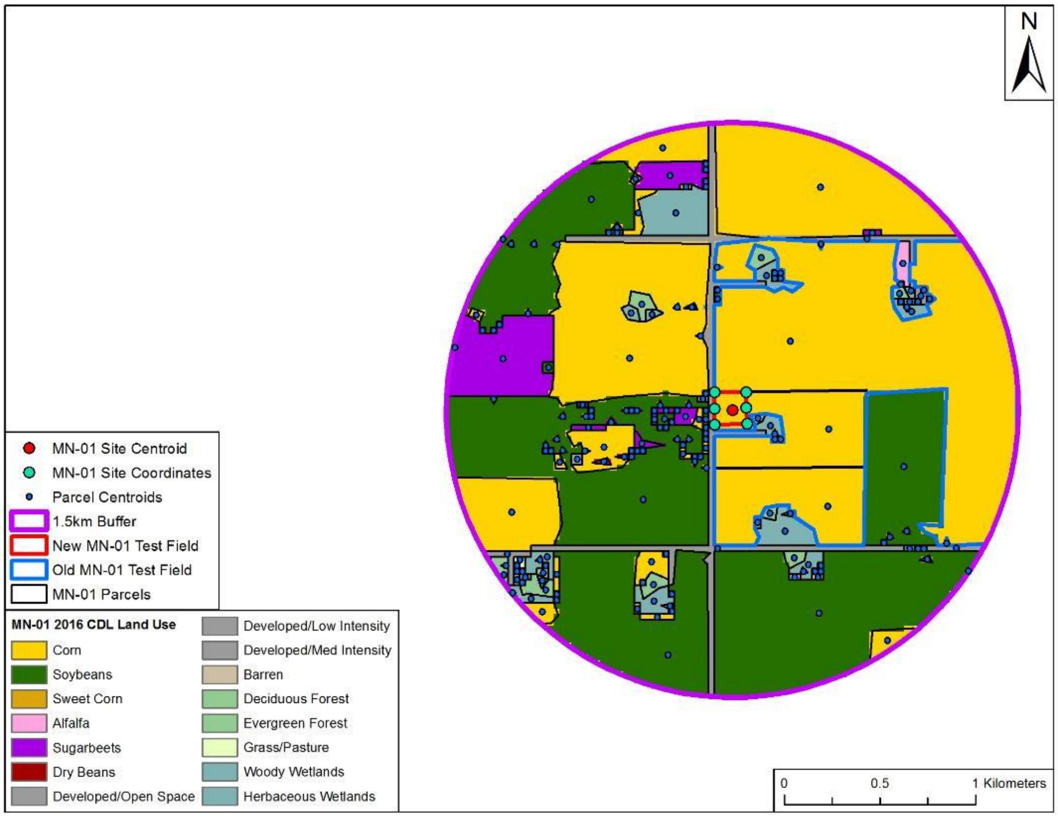

MN-02

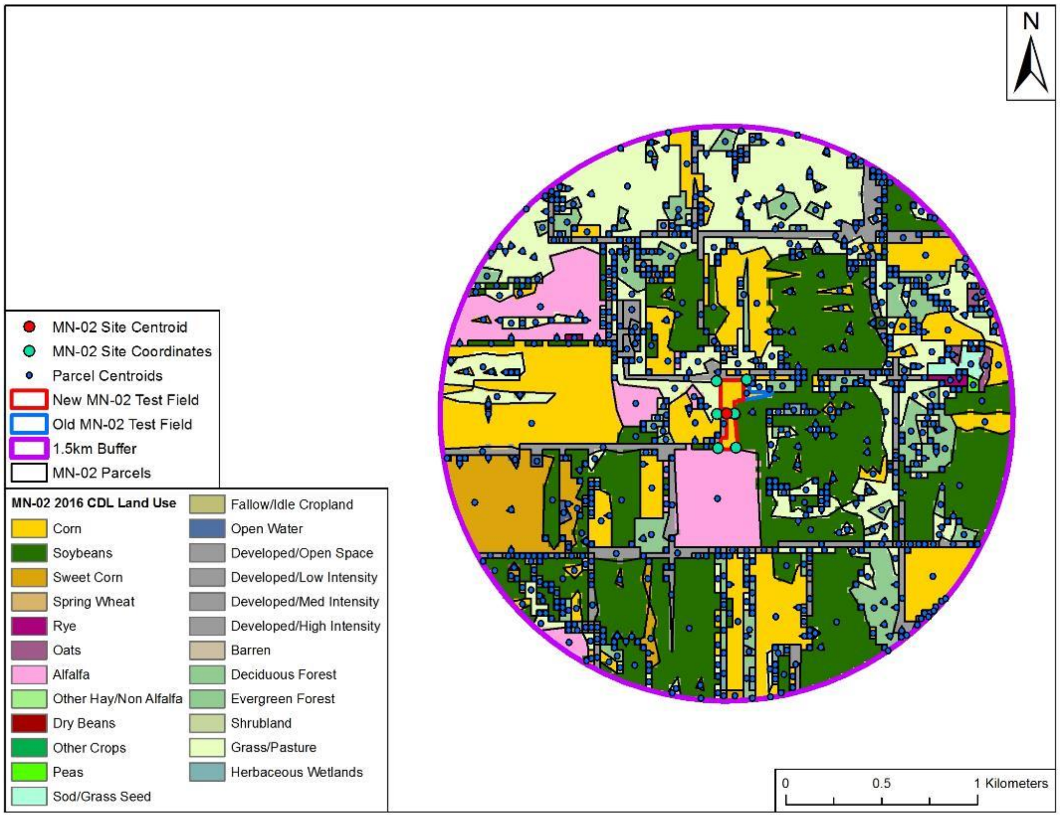
N
MN-02 Site Centroid
MN-02 Site Coordinates
Parcel Centroids
New MN-02 Test Field
Old MN-02 Test Field
1.5km Buffer
MN-02 Parcels
MN-02 2016 CDL Land Use
Corn
Soybeans
Sweet Corn
Spring Wheat
Rye
Oats
Alfalfa
Other Hay/Non Alfalfa
Dry Beans
Other Crops
Peas
Sod/Grass Seed
Fallow/Idle Cropland
Open Water
Developed/Open Space
Developed/Low Intensity
Developed/Med Intensity
Developed/High Intensity
Barren
Deciduous Forest
Evergreen Forest
Shrubland
Grass/Pasture
Herbaceous Wetlands
0
0.5
1 Kilometers

SD-01

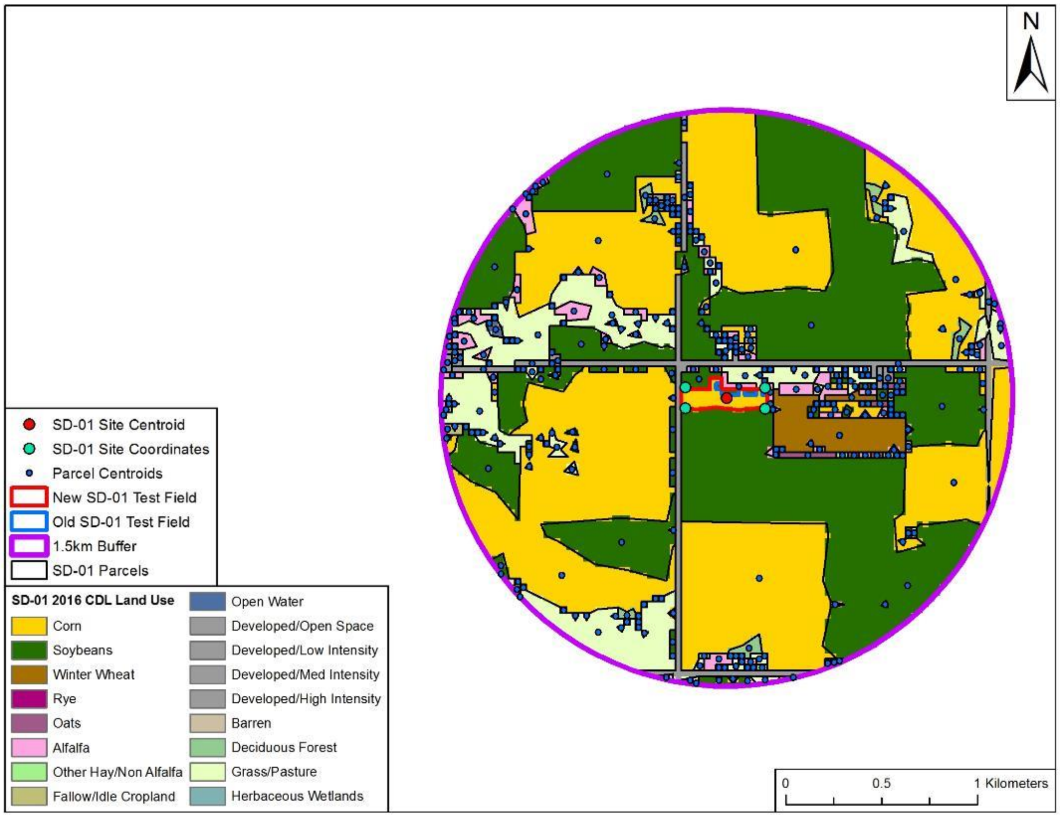
N
SD-01 Site Centroid
SD-01 Site Coordinates
Parcel Centroids
New SD-01 Test Field
Old SD-01 Test Field
1.5km Buffer
SD-01 Parcels
SD-01 2016 CDL Land Use
Corn
Soybeans
Winter Wheat
Rye
Oats
Alfalfa
Other Hay/Non Alfalfa
Fallow/Idle Cropland
Open Water
Developed/Open Space
Developed/Low Intensity
Developed/Med Intensity
Developed/High Intensity
Barren
Deciduous Forest
Grass/Pasture
Herbaceous Wetlands
0
0.5
1 Kilometers

SD-02

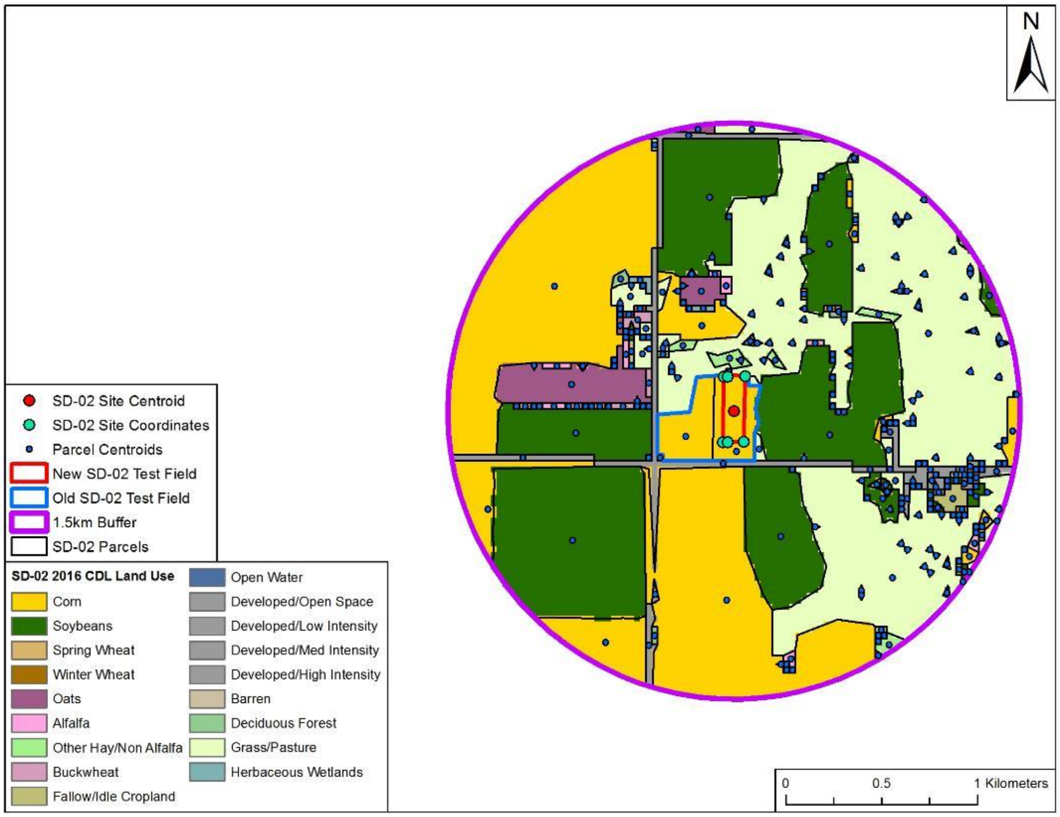
N
SD-02 Site Centroid
SD-02 Site Coordinates
Parcel Centroids
New SD-02 Test Field
Old SD-02 Test Field
1.5km Buffer
SD-02 Parcels
SD-02 2016 CDL Land Use
Corn
Soybeans
Spring Wheat
Winter Wheat
Oats
Alfalfa
Other Hay/Non Alfalfa
Buckwheat
Fallow/Idle Cropland
Open Water
Developed/Open Space
Developed/Low Intensity
Developed/Med Intensity
Developed/High Intensity
Barren
Deciduous Forest
Grass/Pasture
Herbaceous Wetlands
0
0.5
1 Kilometers

SD-03

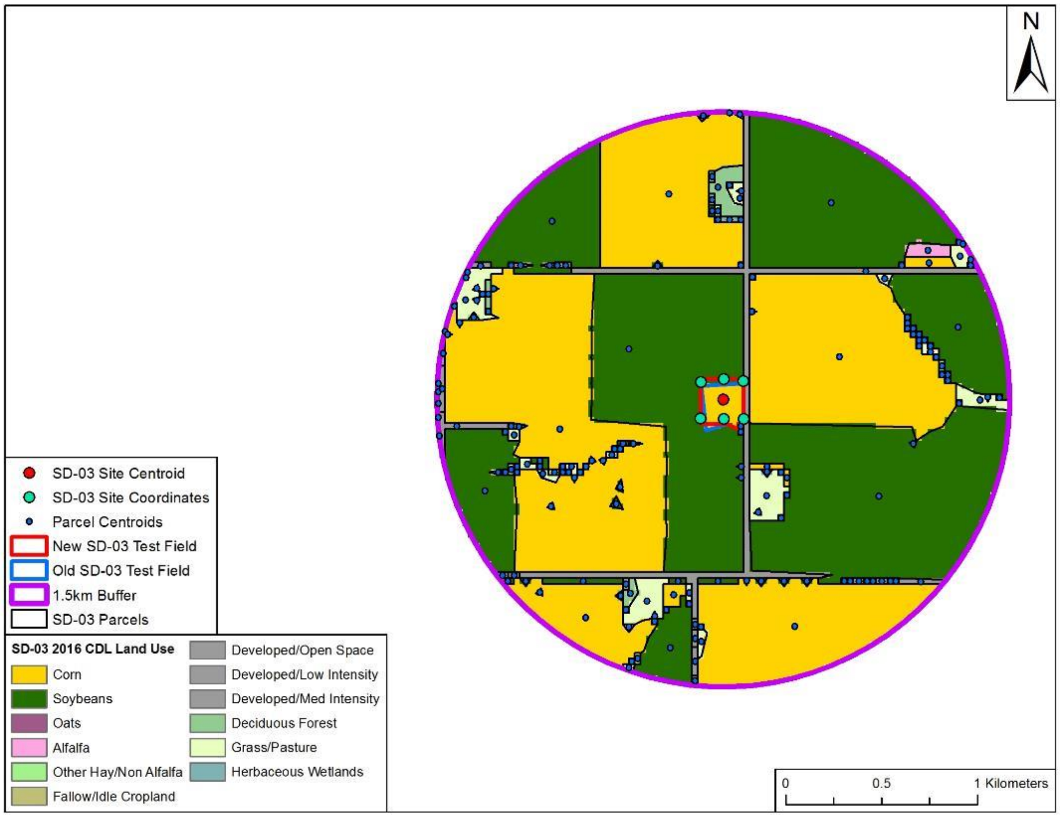
N
SD-03 Site Centroid
SD-03 Site Coordinates
Parcel Centroids
New SD-03 Test Field
Old SD-03 Test Field
1.5km Buffer
SD-03 Parcels
SD-03 2016 CDL Land Use
Corn
Soybeans
Oats
Alfalfa
Other Hay/Non Alfalfa
Fallow/Idle Cropland
Developed/Open Space
Developed/Low Intensity
Developed/Med Intensity
Deciduous Forest
Grass/Pasture
Herbaceous Wetlands
0
0.5
1 Kilometers

WI-01

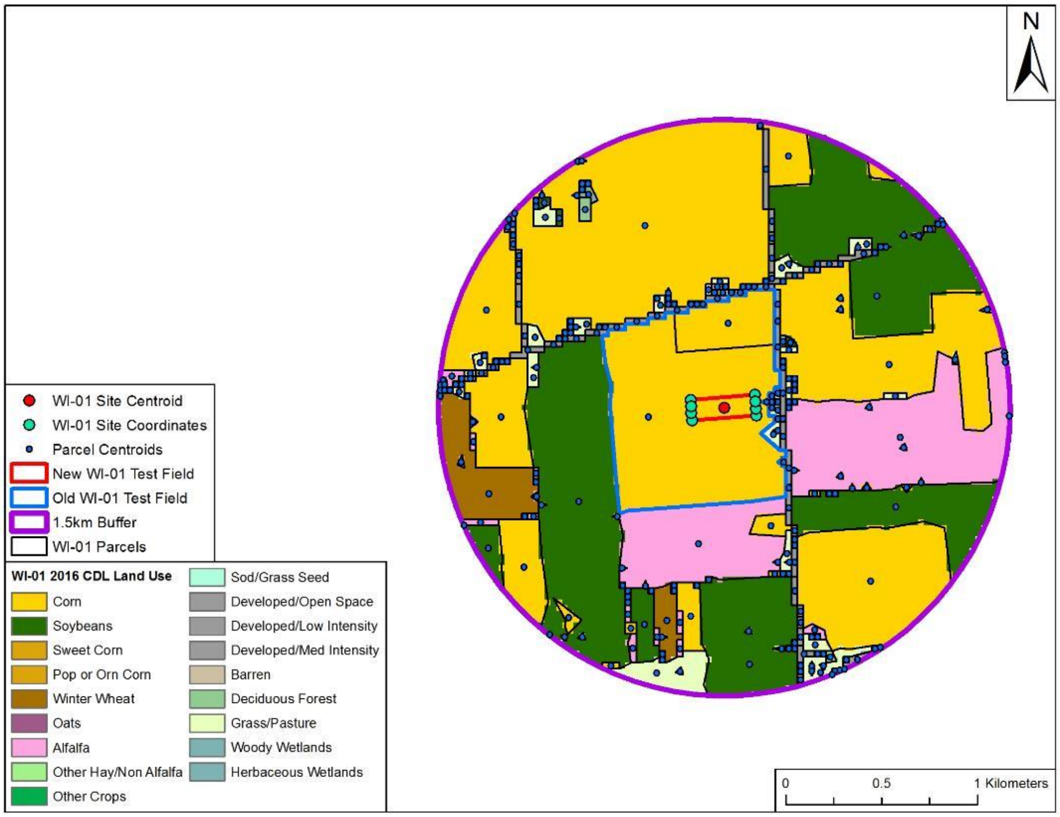
N
WI-01 Site Centroid
WI-01 Site Coordinates
Parcel Centroids
New WI-01 Test Field
Old WI-01 Test Field
1.5km Buffer
WI-01 Parcels
WI-01 2016 CDL Land Use
Corn
Soybeans
Sweet Corn
Pop or Orn Corn
Winter Wheat
Oats
Alfalfa
Other Hay/Non Alfalfa
Other Crops
Sod/Grass Seed
Developed/Open Space
Developed/Low Intensity
Developed/Med Intensity
Barren
Deciduous Forest
Grass/Pasture
Woody Wetlands
Herbaceous Wetlands
0
0.5
1 Kilometers

WI-02

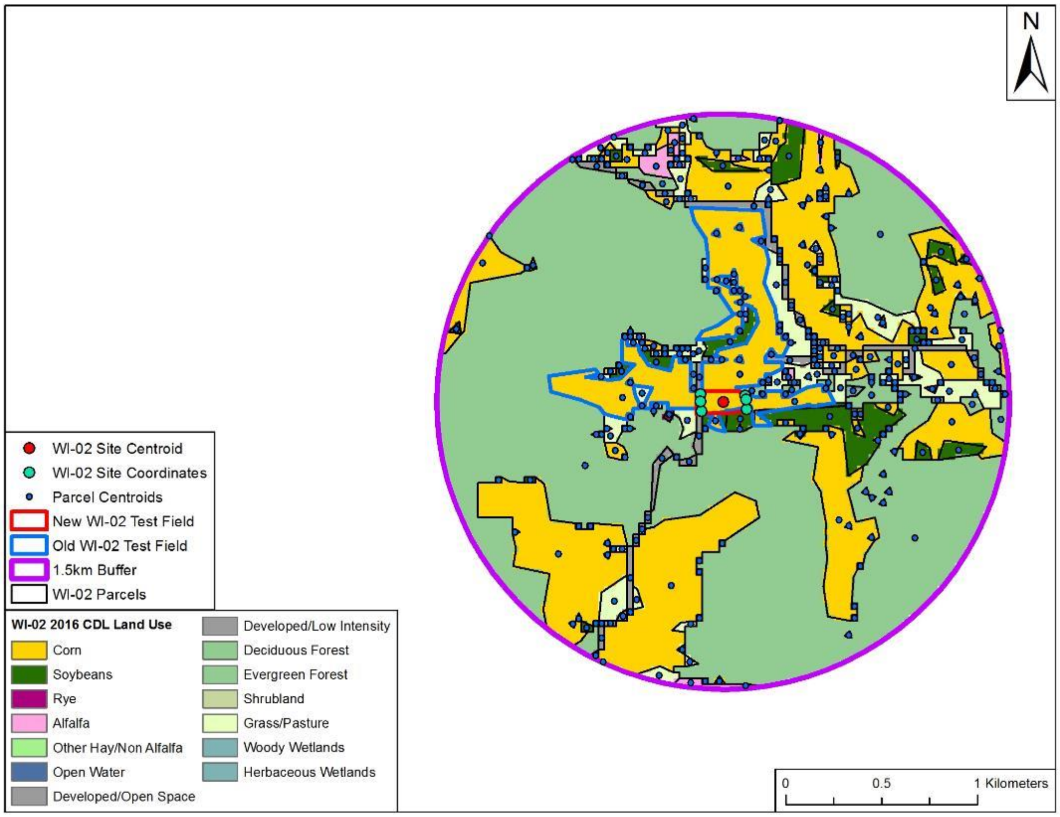
N
WI-02 Site Centroid
WI-02 Site Coordinates
Parcel Centroids
New WI-02 Test Field
Old WI-02 Test Field
1.5km Buffer
WI-02 Parcels
WI-02 2016 CDL Land Use
Corn
Soybeans
Rye
Alfalfa
Other Hay/Non Alfalfa
Open Water
Developed/Open Space
Developed/Low Intensity
Deciduous Forest
Evergreen Forest
Shrubland
Grass/Pasture
Woody Wetlands
Herbaceous Wetlands
0
0.5
1 Kilometers

WI-03

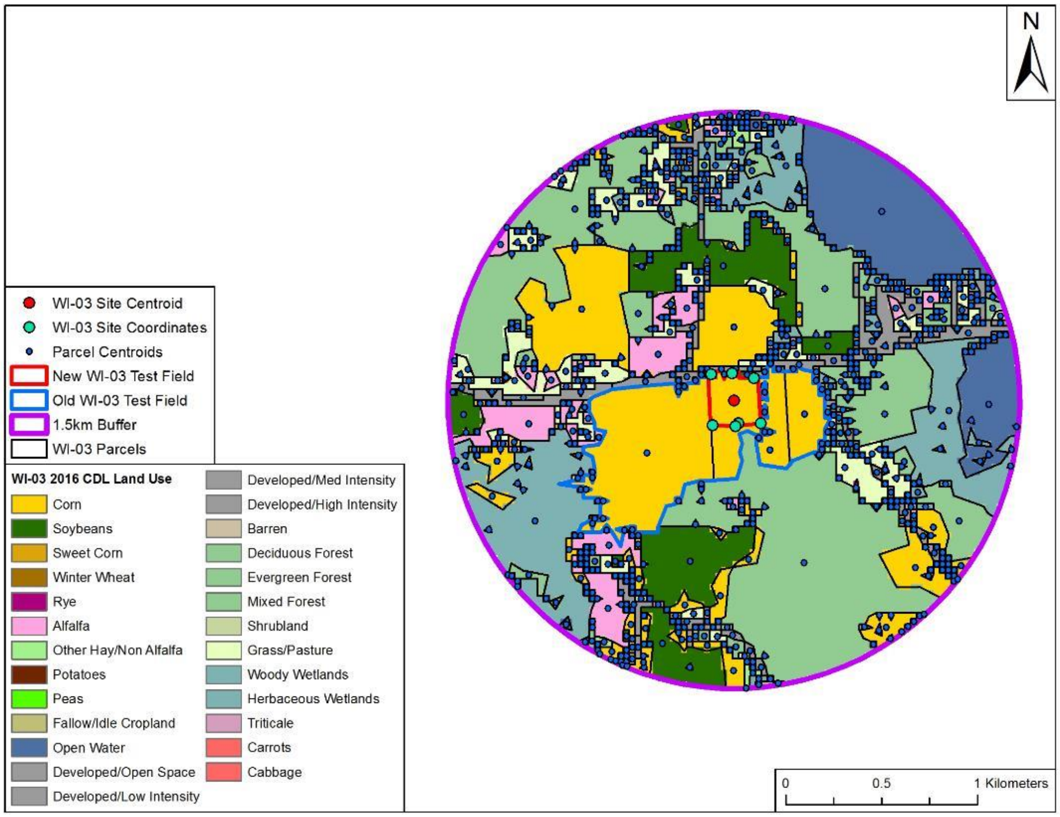
N
WI-03 Site Centroid
WI-03 Site Coordinates
Parcel Centroids
New WI-03 Test Field
Old WI-03 Test Field
1.5km Buffer
WI-03 Parcels
WI-03 2016 CDL Land Use
Corn
Soybeans
Sweet Corn
Winter Wheat
Rye
Alfalfa
Other Hay/Non Alfalfa
Potatoes
Peas
Fallow/Idle Cropland
Open Water
Developed/Open Space
Developed/Low Intensity
Developed/Med Intensity
Developed/High Intensity
Barren
Deciduous Forest
Evergreen Forest
Mixed Forest
Shrubland
Grass/Pasture
Woody Wetlands
Herbaceous Wetlands
Triticale
Carrots
Cabbage
0
0.5
1 Kilometers

WI-04

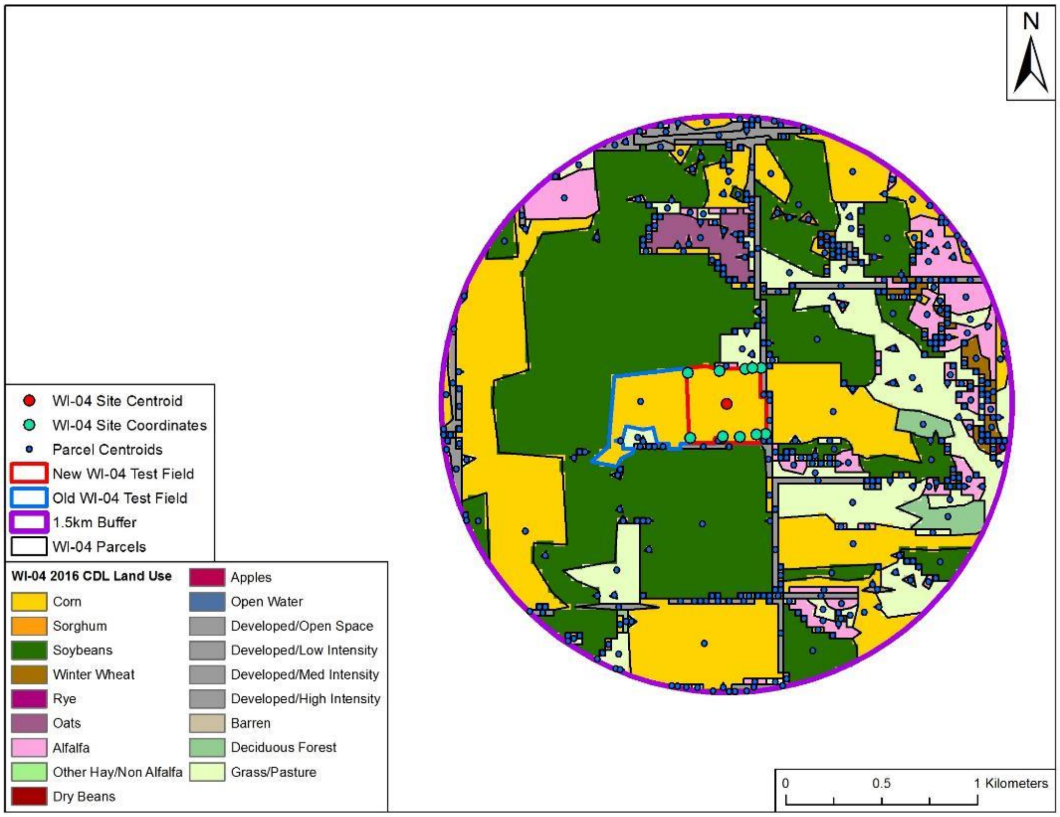
N
WI-04 Site Centroid
WI-04 Site Coordinates
Parcel Centroids
New WI-04 Test Field
Old WI-04 Test Field
1.5km Buffer
WI-04 Parcels
WI-04 2016 CDL Land Use
Corn
Sorghum
Soybeans
Winter Wheat
Rye
Oats
Alfalfa
Other Hay/Non Alfalfa
Dry Beans
Apples
Open Water
Developed/Open Space
Developed/Low Intensity
Developed/Med Intensity
Developed/High Intensity
Barren
Deciduous Forest
Grass/Pasture
0
0.5
1 Kilometers

WI-05

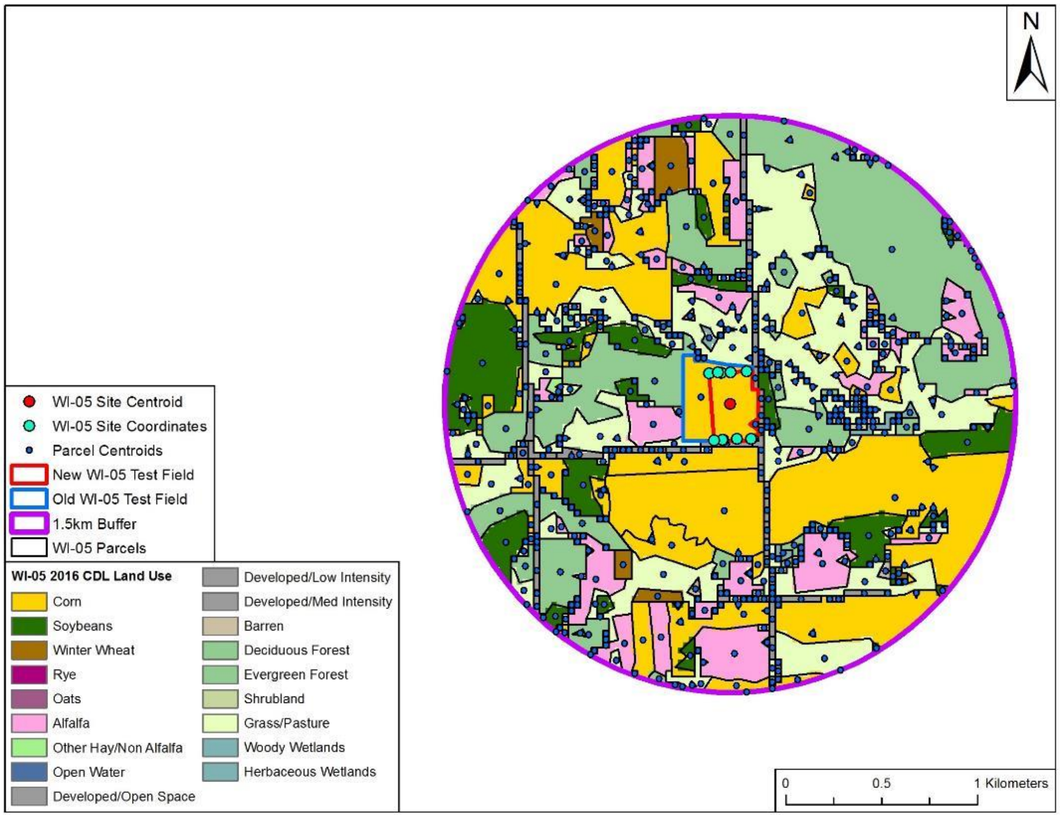
N
WI-05 Site Centroid
WI-05 Site Coordinates
Parcel Centroids
New WI-05 Test Field
Old WI-05 Test Field
1.5km Buffer
WI-05 Parcels
WI-05 2016 CDL Land Use
Corn
Soybeans
Winter Wheat
Rye
Oats
Alfalfa
Other Hay/Non Alfalfa
Open Water
Developed/Open Space
Developed/Low Intensity
Developed/Med Intensity
Barren
Deciduous Forest
Evergreen Forest
Shrubland
Grass/Pasture
Woody Wetlands
Herbaceous Wetlands
0
0.5
1 Kilometers

WI-06

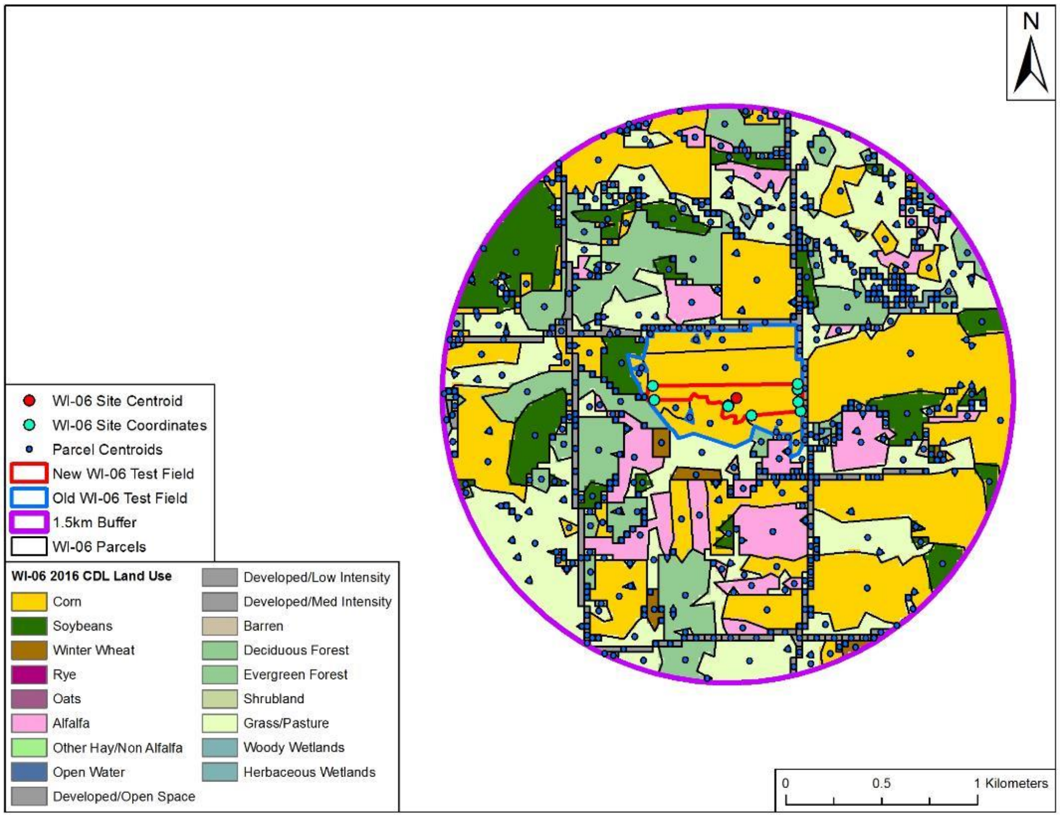
N
WI-06 Site Centroid
WI-06 Site Coordinates
Parcel Centroids
New WI-06 Test Field
Old WI-06 Test Field
1.5km Buffer
WI-06 Parcels
WI-06 2016 CDL Land Use
Corn
Soybeans
Winter Wheat
Rye
Oats
Alfalfa
Other Hay/Non Alfalfa
Open Water
Developed/Open Space
Developed/Low Intensity
Developed/Med Intensity
Barren
Deciduous Forest
Evergreen Forest
Shrubland
Grass/Pasture
Woody Wetlands
Herbaceous Wetlands
0
0.5
1 Kilometers

WI-07

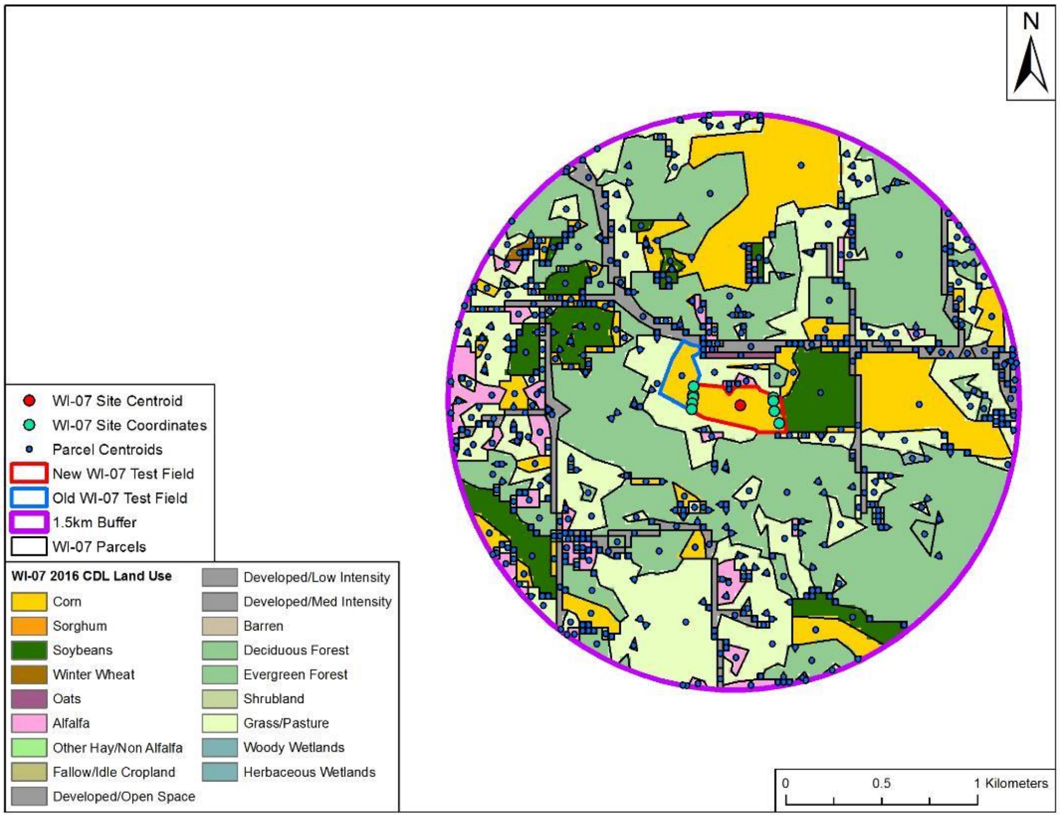
N
WI-07 Site Centroid
WI-07 Site Coordinates
Parcel Centroids
New WI-07 Test Field
Old WI-07 Test Field
1.5km Buffer
WI-07 Parcels
WI-07 2016 CDL Land Use
Corn
Sorghum
Soybeans
Winter Wheat
Oats
Alfalfa
Other Hay/Non Alfalfa
Fallow/Idle Cropland
Developed/Open Space
Developed/Low Intensity
Developed/Med Intensity
Barren
Deciduous Forest
Evergreen Forest
Shrubland
Grass/Pasture
Woody Wetlands
Herbaceous Wetlands
0
0.5
1 Kilometers

WI-08

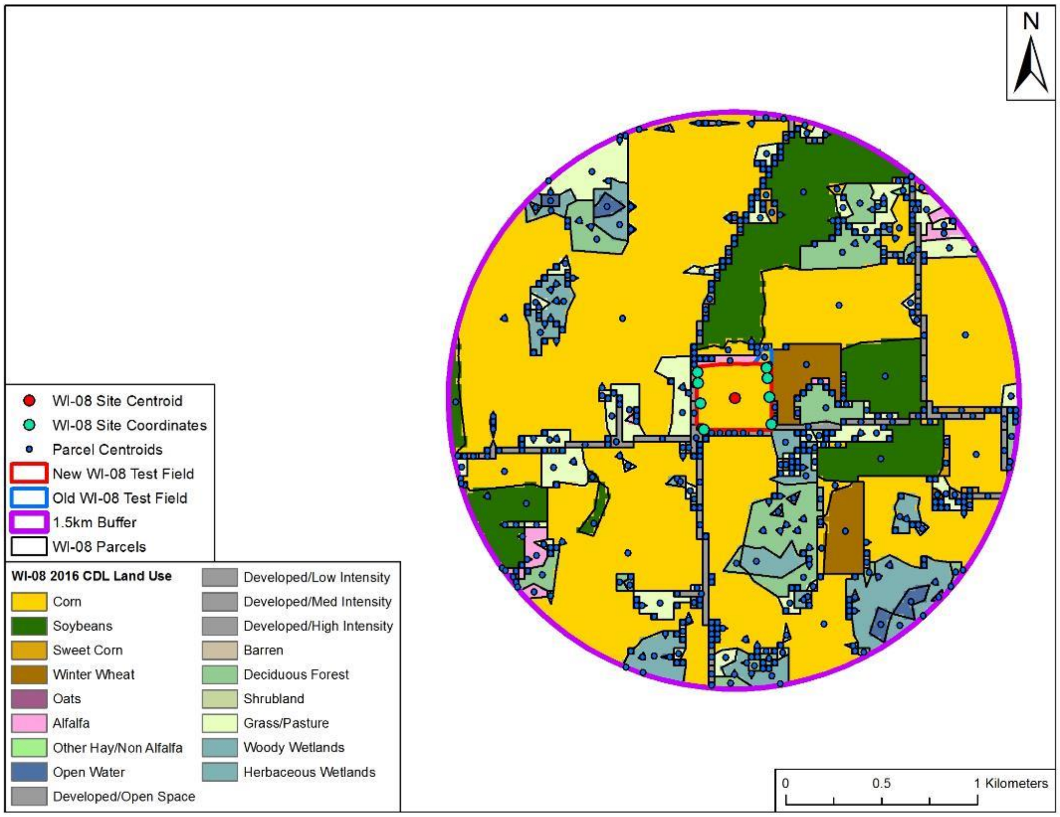
N
WI-08 Site Centroid
WI-08 Site Coordinates
Parcel Centroids
New WI-08 Test Field
Old WI-08 Test Field
1.5km Buffer
WI-08 Parcels
WI-08 2016 CDL Land Use
Corn
Soybeans
Sweet Corn
Winter Wheat
Oats
Alfalfa
Other Hay/Non Alfalfa
Open Water
Developed/Open Space
Developed/Low Intensity
Developed/Med Intensity
Developed/High Intensity
Barren
Deciduous Forest
Shrubland
Grass/Pasture
Woody Wetlands
Herbaceous Wetlands
0
0.5
1 Kilometers

3.3. Methods for estimating bee resources from the landscape

A honey bee colony will experience variable resource levels in the landscape around the bee hive dependent on time of year, species and densities of flowers present in the landscape. To set a simulated bee colony in a specific and realistic landscape context, the daily resources in terms of pollen and nectar availabilities have to be estimated.

In BEEHAVE, resources around the simulated bee colony are defined by several parameters. The landscape is assumed to be subdivided into distinct “flower patches.” Within each patch, pollen and nectar availability is assumed to be uniform (though variable in time). Flower patches are defined by their distance to the hive and their area. These parameters are constant for each patch. In addition, the total volume of nectar in each flower patch is defined for each day (in liters/m^2), the sugar concentration of the nectar (in mol/l) and the amount of pollen (in kg/m^2). The time it takes bees to gather a full crop of nectar or a full load of pollen (both pollen baskets filled) is also defined for each patch as nectar and pollen gathering times, respectively. Gathering times do not include the travel time from the colony to the flower patch and back, but only the time a bee is assumed to spent at the patch. In a real forager bee, gathering times for pollen and nectar are dependent on several factors that may include flower density, flower morphology, pollen or nectar amount available from a single flower and other factors. For BEEHAVE, the gathering times have to be determined as input for each flower patch. As default value, 1200s are assumed as gathering time for a full nectar crop, and 600s for a full load of pollen. These estimates are based on average observed gathering times as reported by Winston (1987). Observed gathering times range widely, and may exceed the estimates as used in BEEHAVE considerably (Winston 1987, Thompson et al. 2015).

For the landscape resource input to BEEHAVE, we are assuming that identical land cover types across all sites in the current study will provide the same resource levels per area. In Table S2, we list all land covers present around the study sites in 2016 according to the Crop Data Layer (CDL). For the crops, we list the classification provided by USDA (2015) that determines whether a crop is a bee resource or not. Crops classified to provide no resources for honey bees are not represented in BEEHAVE input. Crops with very low area around the sites and low classification accuracy by CDL were also removed from representation as BEEHAVE landscape resource input. Crops with an area smaller than 1 ha around any of the thirteen sites used in the project, and that are not present at around all sites (within a 3 km radius) were not represented as bee resources in the BEEHAVE input (Table S2). The following crops were represented as bee resources: alfalfa, beans and peas (representing all bean and pea crops except for soybean), buckwheat, corn (representing all corn varieties including sweet, pop and ornamental corn), sorghum (also used to represent millet) and soybean. For each crop, the nectar and pollen availabilities per area were estimated from nectar amount and concentration as well as pollen amount available per flower (Tables S4-S9). The estimation of bee resources from semi-natural land covers is described in section 4.3.

Table S2. Land covers found around the study sites from 2016 (3 km radius). The accuracy of land cover classification for each land cover according to CDL by state. The accuracy reports the % of plots that were correctly classified according to ground checks by producers. For crops, the attractiveness to honey bees is stated according to USDA (2015): "-" = not attractive, "+" = attractive under certain conditions, and "++" = high attractiveness. Crops were not represented in BEEHAVE input if a) they provide no bee resource according to USDA (2015), b) the area of the crop was very low and/or the classification accuracy was low. Semi-natural land covers were either not represented as BEEHAVE resource or classified as one of the seven land cover categories assessed for vegetation composition around the sites.

**Land cover (crop or land cover type)**	**Present at sites**	**Area by site**	**Classification accuracy**	**Attractiveness according to USDA 2015 (pollen/nectar)**	**Represented as BEEHAVE input**
Alfalfa	All	>1 ha at all sites	>70% in SD, MN, WI	+/++	Yes: alfalfa (crop)
Apples	WI-04	7387 m^2	29% (n=34)	++/+	No: low area
Barley	MN-02	16010 m^2		-/-	No: no bee resource
Barren	WI-01, WI-03, WI-04, WI-05, WI-06, WI-07, WI-08, MN-01, MN-02, SD-01, SD-02, SD-03	>1 ha, 2115 m^2, 3885 m^2, >1 ha, >1 ha, 900 m^2, 2700 m^2, >1 ha, >1 ha, 1800 m^2, >1 ha, 6300 m^2	NA	NA	No: assume no resource due to low vegetation
Buckwheat	SD-02	24079 m^2	50% (n=2)	+/++	Yes: buckwheat (crop)
Cabbage	WI-01, WI-03	900 m^2; 1800 m^2	66.7% (n=433)	++/++ (Requires pollination only when grown for seed. Small % of acreage)	No: low area
Carrots	WI-03	900 m^2	54.1% (n=399)	+/+	No: low area
Christmas Trees	WI-03	3600 m^2	19% (n=137)	NA	No: low area (only potential source of honeydew)
Corn	All	>1 ha at all sites	97.2% (n=272115)	+/-	Yes: corn (crop)

Land cover (crop or land cover type)	Present at sites	Area by site	Classification accuracy	Attractiveness according to USDA 2015 (pollen/nectar)	Represented as BEEHAVE input
Dbl Crop WinWht/Corn	WI-02	900 m^2	11.3% (n=737)	See winter wheat/ corn	Yes: as corn (crop)
Dbl Crop WinWht/Soybeans	WI-01, WI-04	900 m^2; 900 m^2	26.4% (n=106)	See winter wheat/ soybeans	Yes: as soybeans (crop)
Deciduous Forest	All	>1 ha	WI: 88.8% (n=168426); MN: 80.3% (n=19.158); SD: 71.1% (n=3871)	NA	Yes: deciduous forest (semi-natural land cover)
Developed/ High intensity	WI-01; WI-02; WI-03; WI-04; WI-05; WI-06; WI-07; WI-08; MN-02; SD-01; SD-02; SD-03	5400 m^2; 900 m^2 3600 m^2; 8100 m^2; 900 m^2; 900 m^2; 900 m^2; 1800 m^2; >1 ha; 4500 m^2; 2700 m^2; >1 ha	WI: 88.7% (n=1314); MN: 85.3% (n=1227); SD: 79.7% (n=227)	NA	No: no information on vegetation cover on developed lands
Developed/ Low intensity	All	>1 ha	WI: 84.2% (n=11695); MN: 76.5% (n=7677); SD: 75.7% (n=2015)	NA	No: no information on vegetation cover on developed lands
Developed/Medium intensity	WI-01; WI-03; WI-04; WI-05; WI-06; WI-07; WI-08; MN-01; MN-02; SD-01; SD-02; SD-03	>1 ha; 5756 m^2; >1 ha; 3600 m^2; 4500 m^2; 7200 m^2; 9900 m^2; >1 ha; >1 ha; >1 ha; >1 ha; >1 ha	WI: 82.7% (n=4230); MN: 76.8% (n=3850); SD: 75.9% (n=856)	NA	No: no information on vegetation cover on developed lands

**Land cover (crop or land cover type)**	**Present at sites**	**Area by site**	**Classification accuracy**	**Attractiveness according to USDA 2015 (pollen/nectar)**	**Represented as BEEHAVE input**
Developed/Open space	All	>1 ha	WI: 93.5% (n=24169); MN: 94.2% (n=25603); SD: 92.5% (n=14824)	NA	No: no information on vegetation cover on developed lands
Dry beans	WI-03; WI-04; WI-08; MN-01; MN-02	>1 ha; 1800 m2; 1800 m2; >1 ha; >1 ha	WI: 74.9% (n=6058); MN: 75.8% (n=5076)	+/+	Yes: as beans and peas (crop)
Evergreen forest	WI-02; WI-03; WI-04; WI-05; WI-06; WI-07; WI-08; MN-01; MN-02	7200 m^2; >1 ha; 1800 m^2; 3015 m^2; 3015 m^2; >1 ha; >1 ha; 900 m^2; >1 ha	WI: 37.9% (n=12960); MN: 44.6% (n=19158)	NA	Yes: evergreen forest (semi-natural land cover)
Fallow/Idle cropland	WI-01, WI-03, WI-07, WI-08, MN-02, SD-02, SD-03	>1 ha, 1800 m^2, 900 m^2, 900 m^2, 3956 m^2, >1 ha, 5546 m^2	WI: 18.1% (n=978); MN: 19.4% (n=1043); SD: 74.5% (n=14757)	NA	Yes: as Pasture (semi-natural land cover; assumed most similar semi-natural land cover assessed)
Grass/Pasture	All	>1 ha	WI: 69% (n=11783); MN: 70.4% (n=15174); SD: 97% (n=280125)	NA	Yes: pasture (semi-natural land cover)
Herbaceous Wetlands	All	>1 ha; WI-04: 9000 m^2	WI: 54.6% (n=15779); MN: 62.2% (n=41929); SD: 37.6% (n=5963)	NA	Yes: herbaceous wetland (semi-natural land cover)

**Land cover (crop or land cover type)**	**Present at sites**	**Area by site**	**Classification accuracy**	**Attractiveness according to USDA 2015 (pollen/nectar)**	**Represented as BEEHAVE input**
Millet	SD-02	>1 ha	50% (n=4762)	+/-	Yes: as sorghum (crop; sorghum used as generic wind-pollinated pollen source)
Misc vegs and fruit	MN-02	2700 m^2	0% (n=5)	NA	No: low area, low classification accuracy, classification too broad
Mixed forest	WI-01	1800 m^2	17% (n=19951)	NA	Yes: as deciduous forest (semi-natural land cover)
Oats	WI-01; WI-04; WI-05; WI-06; WI-07; WI-08; MN-01; MN-02; SD-01; SD-02; SD-03	>1 ha for all sites listed; exception: 900 m^2 (MN-01)	WI: 47.7% (n=5697); MN: 19.5% (n=1163); SD: 50.2 (n=8044)	-/-	No: no bee resource
Open water	All	>1 ha for all sites listed; exceptions: WI-01; WI-06; WI-07	WI: 94.9% (n=20102); MN: 94.8% (n=42232); SD: 87.2% (n=19043)	NA	No: no bee resource
Other crops	WI-01; MN-02	1800 m^2; >1 ha	WI: 64.7% (n=34); MN: 77% (n=553)	NA	No: classification too broad

**Land cover (crop or land cover type)**	**Present at sites**	**Area by site**	**Classification accuracy**	**Attractiveness according to USDA 2015 (pollen/nectar)**	**Represented as BEEHAVE input**
Other hay/non alfalfa	WI-01; WI-02; WI-03; WI-04; WI-05; WI-06; WI-07; WI-08; MN-01; MN-02; SD-01; SD-02; SD-03	>1 ha; 2700 m^2; >1 ha; >1 ha; >1 ha; >1 ha; >1 ha; >1 ha; 3600 m^2; >1 ha; >1 ha; >1 ha; >1 ha	WI: 40.2% (n=12279); MN: 24.2% (n=4592); SD: 73.9% (n=58317)	NA	Yes: other hay (semi-natural land cover)
Peas	WI-01; WI-03; WI-05; MN-01; MN-02	>1 ha; 900 m^2; 5715 m^2; >1 ha; >1 ha	WI: 64.6% (n=1198); MN: 63.4% (n=895)	+/+	Yes: as beans and peas (crop)
Pop or Orn Corn	WI-01	8677 m^2	12.3% (n=81)	+/- (for corn)	Yes: as corn (crop)
Potatoes	WI-03	>1 ha	86% (n=6533)	-/-	No: not classified as resource for honey bees (but for other bee species); requires pollination only for breeding; small % of acreage
Pumpkins	WI-01; MN-02	6656 m^2; 5400 m^2	WI: 6% (n=53); MN: 0% (n=9)	+/+	No: low acreage, low classification accuracy

**Land cover (crop or land cover type)**	**Present at sites**	**Area by site**	**Classification accuracy**	**Attractiveness according to USDA 2015 (pollen/nectar)**	**Represented as BEEHAVE input**
Rye	WI-01; WI-02; WI-03; WI-04; WI-05; WI-06; MN-01; MN-02; SD-01; SD-03	1800 m^2; 5427 m^2; 4896 m^2; 2700 m^2; 3600 m^2; 2700 m^2; 3600 m^2; >1 ha; 5400 m^2; 900 m^2	WI: 57.9% (n=1766); MN: 56.4% (n=720); SD: 54.5% (n=732)	-/-	No: no bee resource
Shrubland	All WI and MN sites	>1 ha for all WI sites, MN-01: 900 m^2; MN-02: >1 ha	WI: 9.1% (n=8640); MN: 16.1% (n=13710)	NA	Yes: shrubland (semi-natural land cover)
Sod/Grass Seed	WI-01; MN-02	3600 m^2; >1 ha	WI: 61.8% (n=249); MN: 86.3% (n=2779)	+/- (for grasses)	No: wind-pollinated grass assumed to provide no relevant resource for bees
Sorghum	WI-04; WI-07; SD-03	>1 ha; 3600 m^2; 1800 m^2	WI: 12% (n=380); SD: 66% (n=10536)	+/-[1]	Yes: sorghum (crop)
Soybeans	All	>1 ha	WI: 95% (n= 141651); MN: 98% (n=238449); SD: 97.1% (n=172483)	+/+	Yes: soybeans (crop)
Spring Wheat	MN-01; MN-02; SD-01; SD-02; SD-03	>1 ha; >1 ha; 900; 1800; >1 ha	MN: 97.3% (n=41557); SD: 93.4% (n=41341)	-/-	No: no bee resource
Sugarbeets	MN-01	901469 m^2	95% (n=13041)	-/+	No: requires pollination only for breeding; small % of acreage

**Land cover (crop or land cover type)**	**Present at sites**	**Area by site**	**Classification accuracy**	**Attractiveness according to USDA 2015 (pollen/nectar)**	**Represented as BEEHAVE input**
Sunflower	SD-03	900	90.2% (n=23205)	++/++	No: very low area
Sweet Corn	WI-01;<br>WI-03;<br>WI-07;<br>WI-08<br>MN-01;<br>MN-02	>1 ha;<br>>1 ha;<br>900 m^2;<br>>1 ha;<br>>1 ha;<br>>1 ha	WI: 77.3% (n=4618);<br>MN: 84.1% (n=3134)	+/- (for corn)	Yes: as corn (crop)
Tobacco	WI-08	6300 m^2	0% (n=3)	+/-	No: low acreage, low classification accuracy
Triticale	WI-03;<br>SD-02	2156 m^2;<br>>1 ha	WI: 33% (n=394);<br>SD: 9.9% (n=151)	-/-	No: no bee resource
Winter Wheat	WI-01;<br>WI-03;<br>WI-04;<br>WI-05;<br>WI-06;<br>WI-07;<br>WI-08;<br>MN-02;<br>SD-01;<br>SD-02;<br>SD-03	>1 ha;<br>8100 m^2;<br>>1 ha;<br>>1 ha;<br>>1 ha;<br>>1 ha;<br>>1 ha;<br>900 m^2;<br>>1 ha;<br>>1 ha;<br>1800 m^2	WI: 93.4% (17689);<br>MN: 34.1% (n=276);<br>SD: 94.9% (n=47437)	-/-	No: no bee resource
Woody wetlands	WI-01;<br>WI-02;<br>WI-03;<br>WI-04;<br>WI-05;<br>WI-06;<br>WI-07;<br>WI-08;<br>MN-01;<br>MN-02;<br>SD-01;<br>SD-02;<br>SD-03	>1 ha;<br>>1 ha;<br>>1 ha;<br>900 m^2;<br>7200 m^2;<br>7200 m^2;<br>>1 ha;<br>>1 ha;<br>>1 ha;<br>5400 m^2;<br>6300 m^2;<br>8100 m^2;<br>1800 m^2	WI: 60% (n=61386);<br>MN: 72.4% (n=86311);<br>SD: 63.2% (n=3190)	NA	Yes: woody wetland (semi-natural land cover)

[1] Classified as nectar-providing crop by USDA (2015). Sorghum is a wind-pollinated graminoid grass, and is unlikely to produce nectar (see Schmidt and Bothma 2005, Odoux et al. 2012)

3.3.1. Estimation of bee resources from crops

The following values are required to create BEEHAVE flower patch specifications for each crop:

- Daily nectar production (µl/flower)
- Nectar sugar concentration (mol/l)
- Daily pollen production (mg/flower)
- Flowering Period
- Daily flowering density (flower/m^2)

This information was gathered from multiple sources, as are listed in Tables S4-S9 for each crop. Nectar production by flowers may be reported using different units and appropriate measures have to be calculated (volume per flower, and sugar concentration in mol/l).

Nectar calculations

As measures of nectar production, nectar volume (our target measure), nectar mass, sugar concentration in % (weight sugar/weight solution) or g/l) or sugar mass may be reported. Measures of sugar concentration, and their combination with sugar mass was used to calculate sugar concentration in mol/l and nectar volume by applying the following equation:

$$V = \frac{m_B}{\left(\frac{s}{100}\right)\rho} \qquad \text{(S4)}$$

where
V: volume of solution [l]
ρ : density of solution [g/l]
$m_B = n_B * M_B$, or mass of sugar [g]
s: sugar content (% w/w)

Table S3. Densities of sucrose solutions. Density values were selected based on a stated percentage of sugar in solution. If the percentage value did not exactly match, the nearest reference value was chosen. Values are compiled and verified from online sources[2].

Sucrose content (w/w) (in %)	Density ρ (g/ml)
25	1.104
30	1.127
35	1.151
40	1.176
45	1.203
50	1.230
55	1.258
60	1.286

[2] Online sources of sucrose solution density measures:
http://www.lclane.net/text/sucrose.html
http://homepages.gac.edu/~cellab/chpts/chpt3/table3-2.html
http://wiki.houptlab.org/wiki/Density_of_Sugar_Solutions

For the calculations of sugar concentrations (and nectar volume), we assumed that all sugar occurs in the form of sucrose and used the densities of sucrose solutions as stated in Table 2.

Molar mass of sucrose: 342.3 g/mol

3.3.2. Bee resource specifications for crops

Table S4. Alfalfa (*Medicago sativa*).

**Parameter**	**Value used for BEEHAVE input**	**Uncertainty range**	**References**
Nectar production per flower and day [µl]	0.145 µl	0.29 +/- 0.24 µl per flower reported [1]; nectar production assumed to occur over 2 days as peak nectar production reported to occur over 1-2 days [2]	[1] Cane et al. 2011; [2] Southwick 1984
Nectar sugar concentration (mol/l)	1.25 mol/l	Value uncertain: [1] report 1070 +/- 986 µg/ µl total sugar in nectar which would correspond to 3.13 mol/l; we use the reported sucrose concentration instead (427 +/- 452 µg/µl); [2] estimate of 40% sugar in nectar (corresponds to 1.17 mol/l)	[1] Cane et al. 2011; [2] Southwick 1984
Pollen production per flower and day [µg]	38 µg	[1] 3326 +/- 845 pollen grains per flower; 90mg bee provision contain 33 +/- 5% pollen and 1.3 million pollen grains; [2] note that 2 days of pollen production per flower are assumed.	[1] Cane et al. 2011; [2] Southwick 1984
Flowering Period	1 June – 27 September	Period reported for ND and WY applied; exact dates added (flowering period given as June – September)	[3] Dittberner and Olson 1983[1]
Flower density during flowering period (flowers/m^2)	4984 flowers/m^2	[4] Average 203 plants/m2 (for spring planting, range: 103 - 319); 275 racemes per plant and year (range: 31-782); 5 flowers per raceme (estimate, no quantitative data found); [3] flower period of 112 days; [2] single flower is open for 2 days; flower density is assumed to be uniform across flowering period.	[4] Bagavathiannen 1999

[1]Reference given by:
https://www.fs.fed.us/database/feis/plants/forb/medsat/all.html#BOTANICAL%20AND%20ECOLOGICAL%20CHARACTERISTICS

Table S5. Beans and peas (*Pisum sativum, Vicia faba, Phaseolus* spp.)[1]

**Parameter**	**Value used for BEEHAVE input**	**Uncertainty range**	**References**
Nectar production per flower and day [µl]	0.228 µl	Measured for Vicia faba; nectar production dependent on flower age	[1] Osborne et al. 1997
Nectar sugar concentration (mol/l)	0.768	Measured for Vicia faba; sugar concentration dependent on flower age	[1] Osborne et al. 1997
Pollen production per flower and day [µg]	195 µg	Measured for Vicia Americana; calculated from pollen count and pollen diameter	[1] Vonhof and Harder 1995
Flowering Period	15 June – 15 July	[1] Flowering of single plant given as ~30 days; [2] For Phaseolus spp. In Wisconsin; highly dependent on planting time, temperature and variety; dates	[1] Osborne et al. 1997; [2] Corn Agronomy[2]
Flower density during flowering period (flowers/m^2)	519 flowers/m^2	For 'cultivated bean' across year in Great Britain	[1] Baude et al. 2016

[1] For beans and peas, quantitative measures were not available for each species. The values applied reflect information from a combination of species as indicated.

[2]http://corn.agronomy.wisc.edu/Crops/FieldBean.aspx

Table S6. Buckwheat (*Fagopyrum esculentum*)

**Parameter**	**Value used for BEEHAVE input**	**Uncertainty range**	**References**
Nectar production per flower and day [µl]	0.07 µl	0.05 – 0.09 µl (variance between varieties, see Table 1)	[1] Cawoy et al. 2008
Nectar sugar concentration (mol/l)	2.02 mol/l	Sugar concentration in control treatments stable over 40 days of experiment (see Fig. 3C)	[1] Cawoy et al. 2008
Pollen production per flower and day [µg]	56 µg	[1] number of pollen grains per anther and pollen grain size dependent on buckwheat variety; pollen weight estimated assuming density of water; [2] number of anthers per flower	[1] Cawoy et al. 2006; [2] Björkman and Pearson 1995
Flowering Period	June – September	Duration of flowering corresponds with Cawoy et al. 2008	[1]
Flower density during flowering period (flowers/m^2)	4900 flowers/ m^2	[1] Flowers in anthesis per day and plant: 28.5 (range: 26 – 32); [2] plant density derived from sowing rate: value is a maximum	[1] Cawoy et al. 2006; [2] Pavek 2016

[1] http://wisflora.herbarium.wisc.edu/taxa/index.php?taxon=3615

Table S7. Corn (*Zea mays*)

**Parameter**	**Value used for BEEHAVE input**	**Uncertainty range**	**References**
Nectar production per flower and day [µl]	--	Corn does not produce nectar.	[1] USDA 2015
Nectar sugar concentration (mol/l)	--		[1] USDA 2015
Pollen production per flower and day [µg]	4.3 × 10^5 µg	Uncertainties stem from calculation steps: [2], [3] range of pollen grains per tassel reported; [4] weight of single pollen grain; [2] flowering period, as it is assumed that a tassel produces pollen uniformly across flowering period of 13 days.	[2] Uribellarea et al. 2002; [3] Jarosz et al. 2005; [4] Babendreier et al. 2004
Flowering Period	22 July – 3 August	[5] Ranges given: 19 - 31 July to 26 July- 7 August	[5] Oberhauser 2001
Flower density during flowering period (flowers/m^2)	8 flowers (tassels)/m^2	From planting density of corn; one tassel per plant assumed; variation is low.	

Table S8. Sorghum (*Sorghum spp.*)

**Parameter**	**Value used for BEEHAVE input**	**Uncertainty range**	**References**
Nectar production per flower and day [µl]	--		Odoux et al. 2012
Nectar sugar concentration (mol/l)	--		
Pollen production per flower and day [µg]	116845 µg	Using information from different sources: [1] no. of pollen grains per inflorescence; [2] pollen size (given in µm with a range, pollen weight calculated assuming density of water); [3] flowering duration (given as range of days)	[1] Prieto-Baena et al. 2003; [2] Reddi and Reddi 1986; [3] Gerik et al. 2003
Flowering Period	1 – 8 August	Using information from different sources: [1] flowering duration (given as range of days); [2] flowering timing (highly dependent on planting time; by early August in WI)	[1] Gerik et al. 2003; [2] Carter et al. 1989
Flower density during flowering period (flowers/m^2)	27 inflorescences/m^2	Range of 100000-120000 plants per acre given (singe inflorescence per plant)	Carter et al. 1989

Table S9. Soybean (*Glycine max*)

**Parameter**	**Value used for BEEHAVE input**	**Uncertainty range**	**References**
Nectar production per flower and day [µl]	0.034 µl	[1] Calculated from measured sugar mass produced per flower and sugar concentration, assumption of 2 days anthesis period of single flower	[1] Chiari et al. 2005
Nectar sugar concentration (mol/l)	0.67 mol/l	[1] +/- 10%; measured from honey crops of bees (rather than from nectar collected directly from flowers)	[1] Chiari et al. 2005
Pollen production per flower and day [µg]	19 µg	Pollen grain per anther: average from [2] and [3]; estimate of number of anthers per flower (10); weight of single pollen grain estimated from pollen diameter (as presented in [2])	[2] Koti et al. 2004; [3] Palmer et al. 1978
Flowering Period	5 July – 2 August	May vary by soybean strain planted; time of planting; climate/weather	[4] USDA NASS
Flower density during flowering period (flowers/m^2)	370 flowers/m^2	Uncertain: derived from planting density; estimate of number of pods per plant; % flowers producing pods.	[1]

[1] http://corn.agronomy.wisc.edu/Crops/Soybean/L001.aspx

3.3.3. Estimation of bee resources from semi-natural land covers

Land covers other than crops (semi-natural land covers) provide resources for bees varying over the season dependent on the land cover type and species composition. Specific information on floral resources from the range of flowering plants occurring in the region is not available. Surveys of a subset of semi-natural land covers were conducted around the study fields, and plant species in the survey plots were identified. Using these survey data, we applied categories of bee resource availability to the land covers included in the survey. Bee resources from non-floral sources (e.g., honeydew) were not considered.

Hines and Hendrix (2005) assigned resource categories to different land cover types to estimate resource availability for bumble bees across a landscape. Their resource categories (ranging from 0 to 5) were based on diversity and density of flowering plants in different land covers during surveys. We developed similar categories of honey bee resource availability from semi-natural land covers present around the study fields using qualitative survey data of vegetation conducted around a subset of sites. Plants providing bee resources were assessed in the surveys, and whether they were dominant or not in a surveyed patch. From the five most commonly observed flowering plant species in each land cover type, the flowering period was determined. The estimated vegetative cover of the plant species and the number of species flowering in a given month determined the resource category (Fig. S3, Table S10).

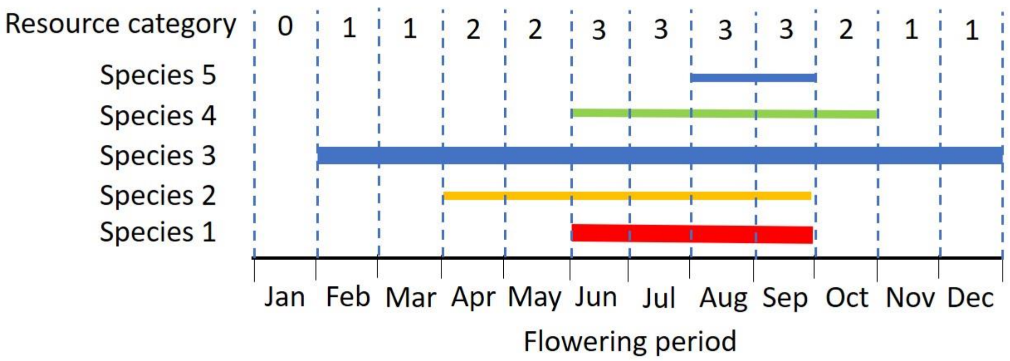

Fig. S3. Example assignment of bee resource categories based on qualitative vegetative cover surveys conducted around a subset of study sites. The category is dependent on number of species flowering in a given month and their vegetative cover (presence/absence and dominant/non-dominant across survey plots in the same land cover).

Table S10. Assignment of bee resource categories is dependent on an estimate of vegetative cover by flowering plant species and how many of the five most commonly observed flowering plants (that provide bee resources) are flowering at the same time.

**Cover\# species flowering**	**0**	**1**	**2**	**3**	**4**	**5**
0%	0	0	0	0	0	0
1-20%	0	1	1	1	1	1
20-40%	0	1	1	1	2	2
40-60%	0	1	2	2	3	3
60-80%	0	2	3	3	4	4
80-100%	0	3	4	4	5	5

Resource categories were applied to each semi-natural land cover and month, i.e. resources were assumed constant throughout each month in a given semi-natural land cover. Resource levels per area in each category are listed in Table S11. Category 0 corresponds to no resource availability, category 5 corresponds to the high bee resource availability, and approximates resource availability from a flowering field of a bee attractive crop such as oilseed rape (Becher et al. 2016).

Table S11. Bee resources assumed to be available from a land cover assigned with a category for BEEHAVE input.

**Category**	**Pollen amount**	**Nectar amount**	**Sugar concentration**	**Pollen gathering time**	**Nectar gathering time**
0	0	0	0	0	0
1	0.2 g/m^2	0.2 ml/m^2	1.5 mol/l	600 s	1200 s
2	0.4 g/m^2	0.4 ml/m^2	1.5 mol/l	600 s	1200 s
3	0.6 g/m^2	0.6 ml/m^2	1.5 mol/l	600 s	1200 s
4	0.8 g/m^2	0.8 ml/m^2	1.5 mol/l	600 s	1200 s
5	1 g/m^2	1 ml/m^2	1.5 mol/l	600 s	1200 s

The land cover types with survey data available were herbaceous wetland, other hay/non-alfalfa, deciduous forest, woody wetland, shrubland, grassy pasture and evergreen forest. From the vegetation survey, resource categories as listed in Table S12 were assigned to these land covers for each month.

Table S12. Bee resource categories assigned to the semi-natural land covers around the study sites.

Time period	Resource category						
	**Herbaceous wetland**	**Other Hay/ Non-alfalfa**	**Deciduous forest**	**Woody wetland**	**Shrubland**	**Grassy pasture**	**Evergreen forest**
Jan	0	0	0	0	0	0	0
Feb	0	1	0	0	0	0	0
Mar	0	1	0	0	0	0	0
Apr	0	2	1	0	0	0	1
May	1	2	1	1	1	0	1
Jun	1	3	1	1	1	1	1
Jul	1	3	2	1	1	1	1
Aug	1	3	1	1	0	1	1
Sep	1	3	1	1	0	1	1
Oct	1	2	1	1	0	1	0
Nov	0	1	0	0	0	0	0
Dec	0	1	0	0	0	0	0

4. CONCEPTUAL MODEL EVALUATION

This document describes the extension of BEEHAVE (Becher et al. 2014) with a pollen exposure-effects module. The conceptual model for the representation of exposures to pesticides in the landscape, the storage of pollen within the colony, and the effects on adult bees and larvae due to consumption of pollen in the hive are derived from the conceptual model presented by EFSA (European Food Safety Authority 2016). Simplifications to the conceptual model were applied, and are described in the main manuscript.

5. IMPLEMENTATION VERIFICATION

5.1. Correspondence of model outputs with BEEHAVE_BeeMapp2015

If no residues are defined in the landscape resource input file (INPUT_FILE: pollenPesticide_ng_per_g = 0 for all patches and days), the extended BEEHAVE model produces the same dynamics as the BEEHAVE version it was based on (BEEHAVE_BeeMapp2015).

5.2. Transfer of residues in landscape patches to stores and cohort exposures

A simple 4-patch landscape input file was used for testing. A generic exposure level was applied to all four patches in the input file during the defined period of corn flowering. Accordingly, all pollen collected during this time period has the same pesticide residue level reflected in the pollen store cohorts in the colony whereby no residues are present in the pollen stores prior to the defined exposure period. The exposure histories due to pollen consumption in larva and adult bee cohorts were also followed, and produced the expected exposure levels.

5.3. Dose-response implementations

The implementation of the dose-response functions was verified by using an external script (implemented in R) of the functions. The proportion of bee deaths calculated from exposure histories in BEEHAVE correspond to the externally implemented dose-responses.

6. MODEL OUTPUT VERIFICATION

The model analysis (section 7) includes the comparison of model outputs with and without simulated residues in corn pollen. No additional explicit model output verifications were conducted with the pollen exposure-effects module for the purpose of the current paper.

7. MODEL ANALYSIS

7.1. Simulation scenarios

7.1.1. Parameter settings

In Table S13, the settings applied to BEEHAVE_BeeMapp2015_PEEM are listed. Parameters defining the dose-response functions are listed in Table S14 (applied to both scenarios), and settings that were equally applied to both scenarios and correspond to BEEHAVE default settings are listed in Table S15. The 20 repetitions of each setting (scenario × site × pollen residue level) were conducted applying the same set of 20 random number seeds to each set. Simulations were run for 480 days (starting with day-of-year 1, or January 1).

Table S13. Settings applied to BEEHAVE_BeeMapp2015_PEEM for the simulation results presented in the current paper. The two scenarios, baseline and stress, are listed separately.

BEEHAVE submodel	Parameter name	Baseline scenario	Stress scenario	Comments
VARROA	N_INITIAL_MITES_INFECTED	0	2000	
BEEKEEPING	HarvestingPeriod	80	100	
	HarvestingTH	20	15	
	HoneyHarvesting	Off	On	
	FeedBees	Off	On	
	MAX_BROODCELLS	72000	72000	corresponds to # cells in a 10 frame Langstroth hive
	MAX_HONEY_STORE_kg	50	50	BEEHAVE default
	Swarming	No swarming	No swarming	
FILES – IN	ReadInfile	On	On	
	INPUT_FILE	Site-specific input files with default gathering times (1200 for nectar, 600 for pollen)	Site-specific input files with nectar gathering time = 2400 s; pollen gathering time = 1200 s	
	Weather	Site-specific weather input file	Site-specific weather input file	Foraging hours not reduced
	ReadFeedingSchedule	Off	Off	

BEEHAVE submodel	Parameter name	Baseline scenario	Stress scenario	Comments
Pollen consumption	DAILY_POLLEN_NEED_IHBEE	6.5	6.5	Use EPA values in both scenarios
	DAILY_POLLEN_NEED_FORAGER	0.041	0.041	
	DAILY_POLLEN_NEED_LARVA	6.53	6.53	
	DAILY_POLLEN_NEED_DRONE_LARVA	5.7	5.7	
	DAILY_POLLEN_NEED_ADULT_DRONE	0.0002	0.0002	

Table S14. Parameters to dose-response functions (applied to both scenarios).

Parameter name	Value
AdultAcuteSlope	2481.06
AdultAcutePower	1.510
AdultChronicSlope	207.17
AdultChronicPower	1.514
LarvaAcuteIntercept	0[1]
LarvaAcuteIntercept	0[1]
LarvaChronicIntercept	0.073
LarvaChronicSlope	1.042

[1] Setting both parameters to zero results in unused dose-response function; study data for larval acute toxicity currently not available and not applied in simulations presented here.

Table S15. Default BEEHAVE settings (from BEEHAVE_BeeMapp2015) applied (unchanged) in the runs with BEEHAVE_BeeMapp2015_PEEM. Only the parameters that are actually in use according to settings are listed.

Parameter name	Value
N_INITIAL_BEES	10000
N_INITIAL_MITES_HEALTHY	0
AllowReinfestation	Off
Virus	DWV
MiteReproductionModel	Martin
VarroaTreatment	Off
HarvestingDay	135
RemainingHoney_kg	5
MergeWeakColonies	Off
AddPollen	Off
HoneyIdeal	Off
ReadBeeMappFile	Off
SeasonalFoodFlow	On
ConstantHandlingTime	Off
AlwaysDance	Off
DANCE_SLOPE	1.16

Parameter name	Value
DANCE_INTERCEPT	0
EggLaying_IH	On
QueenAgeing	Off
MAX_km_PER_DAY	7299
ProbLazinessWinterbees	0
Experiment	none
SQUADRON_SIZE	100
Details	On
WriteFile	Off
modelledInsteadCalcDetectProb	Off
ShowAllPlots	On
stopDead	On

7.1.2. Production of scenario-, site- and pollen residue-level specific input files

For the combinations of scenario, site and pollen residue level, the following landscape resource input files (INPUT_FILE in BEEHAVE) have to be generated:

Input file radius: 1500m (i.e. include all patches with ≤1500m centroid distance from central colony location).
pollenPesticide_ng_per_g for central corn field (distance_m = 0): 0; 12.2; 19; 39.9; 200; 2800

Baseline Scenario:
nectarGathering_s: 1200 for all land covers
pollenGathering_s: 600 for all land covers

Stress Scenario:
nectarGathering_s: 2400 for semi-natural (semi-natural) land covers; 1200 for crops
pollenGathering_s: 1200 for semi-natural land covers; 600 for crops

Weather:
Site-specific weather files (applied to all scenarios).
Note that weather and landscape input files are applied to the first and second simulated year using data from 2016.

7.2. Results

7.2.1. Colony dynamics

Fig. S4 (following pages). All 13 sites' colony dynamics are shown in addition to Fig. 3 in the main manuscript (showing colony dynamics for site WI-08). Each figure is labeled by site and consists of eight plots: numbers of adult worker bees (first column), total number of brood (second column), honey stores (third column), and pollen stores (fourth column), each for the Baseline scenario (row A) and the Stress scenario (row B). Each line represents the average of 20 simulations, colored by corn residue level, and the shaded area shows the range around the averages. Dates marked by solid vertical lines denote the beginning and end of corn tasseling. Dotted vertical lines correspond to the dates used for detailed analysis of adult bee numbers, 21 October of year 1 and 1 April of year 2.

MN-01:

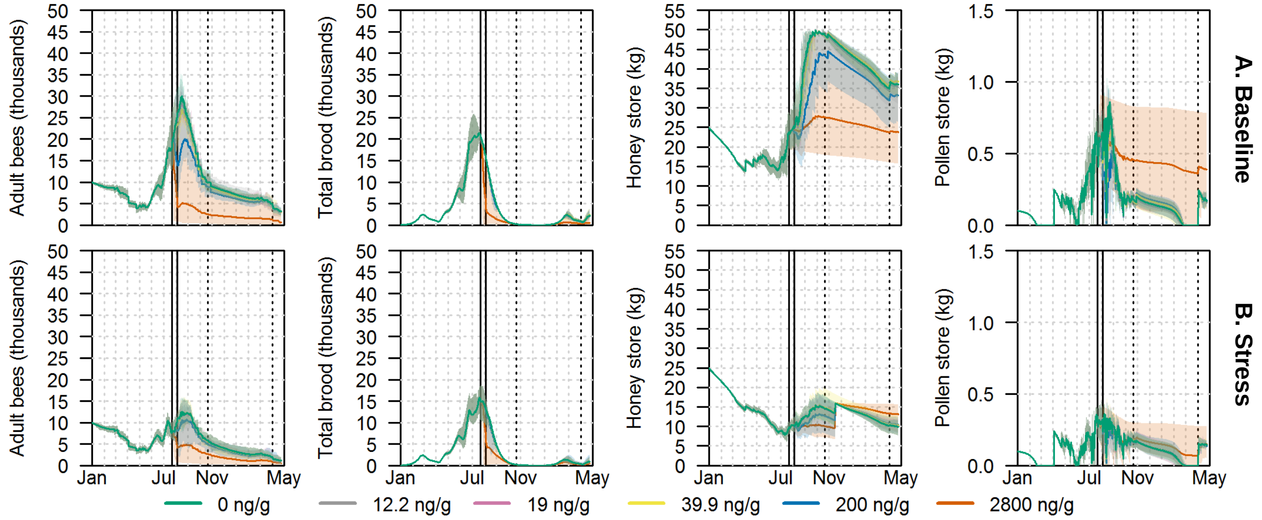

MN-02:

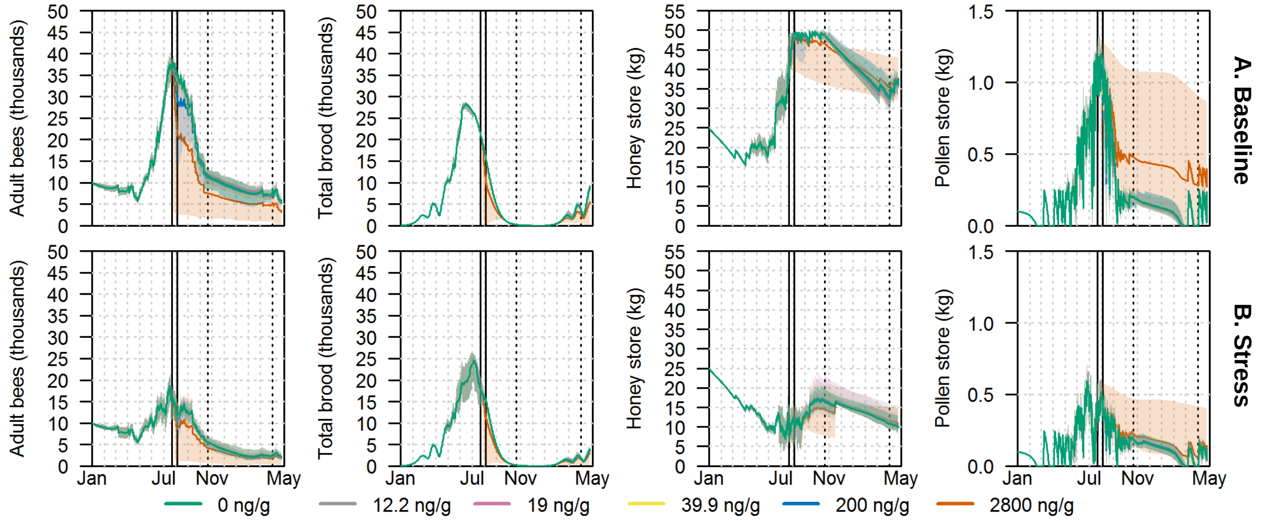

SD-01:

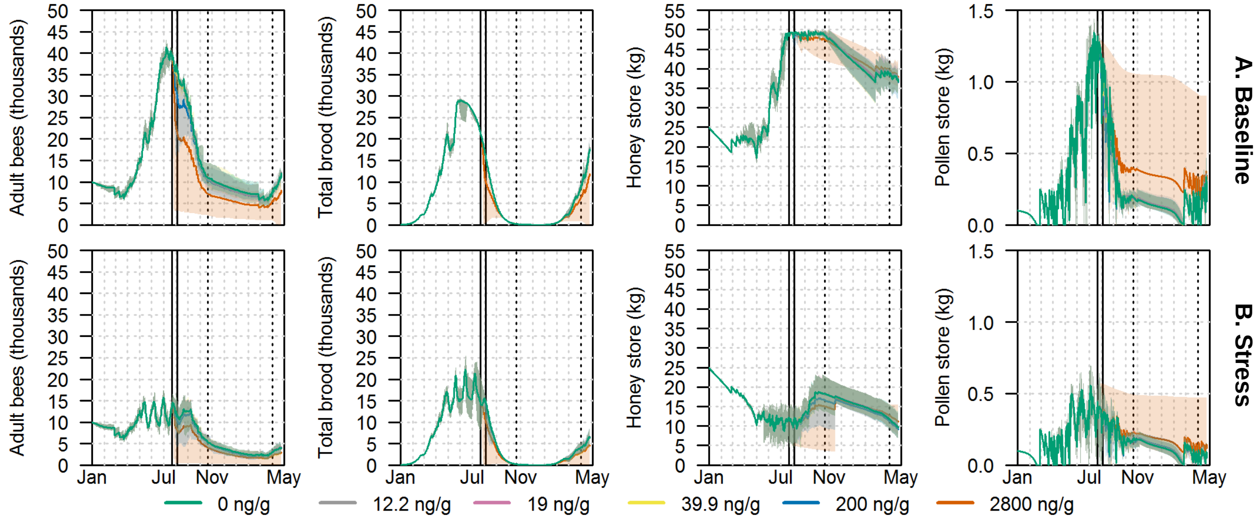

SD-02:

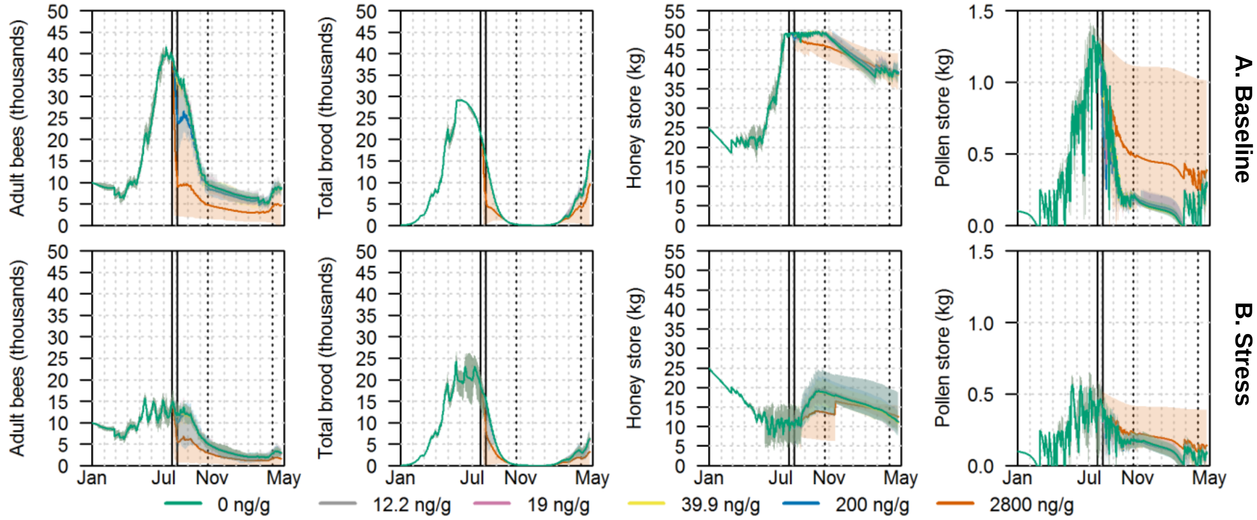

SD-03:

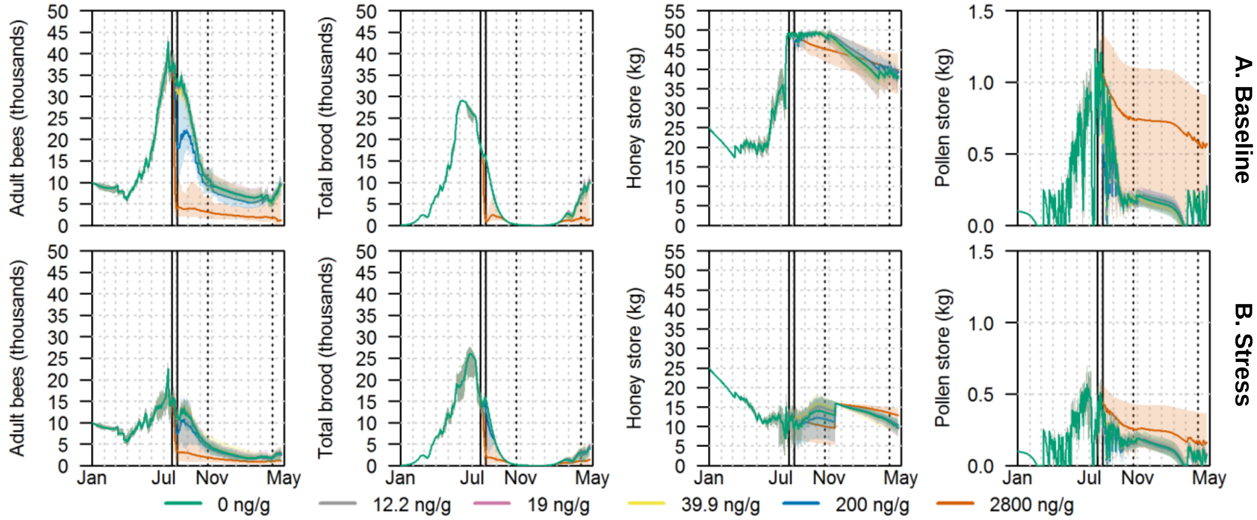

WI-01:

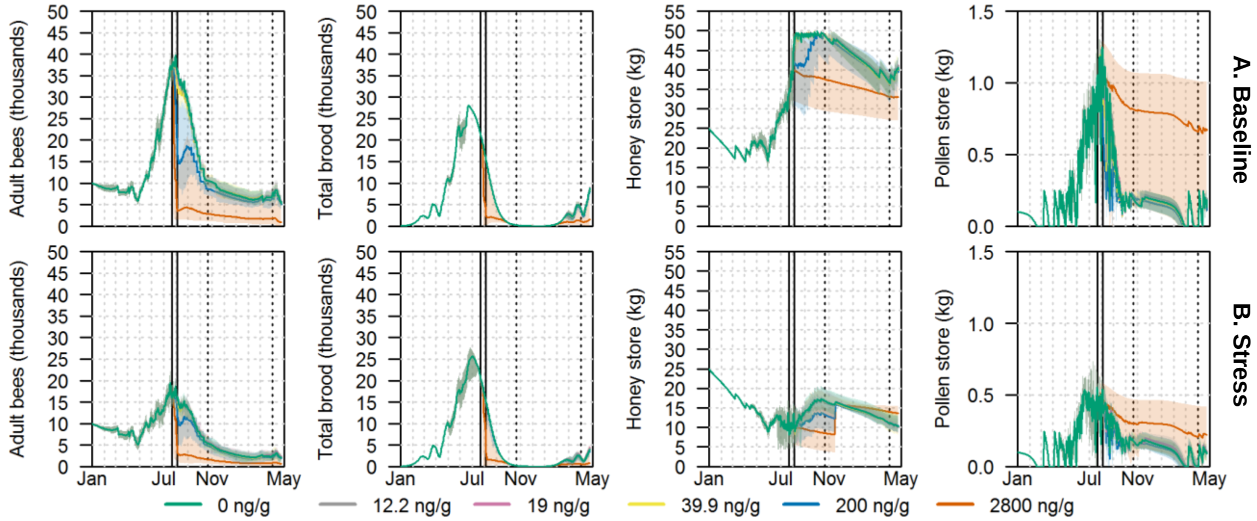

WI-02:

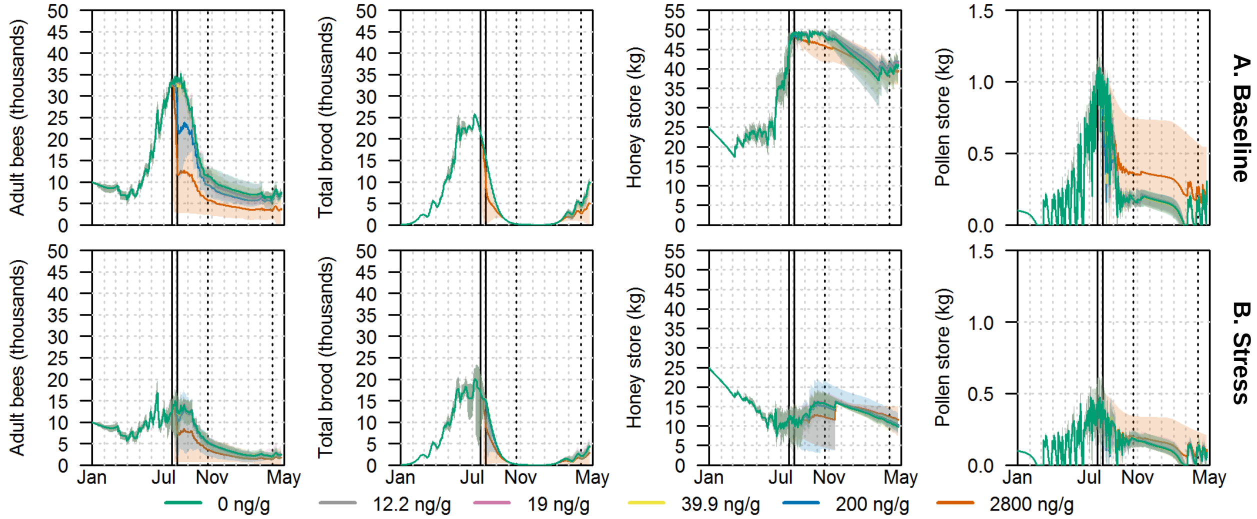

WI-03:

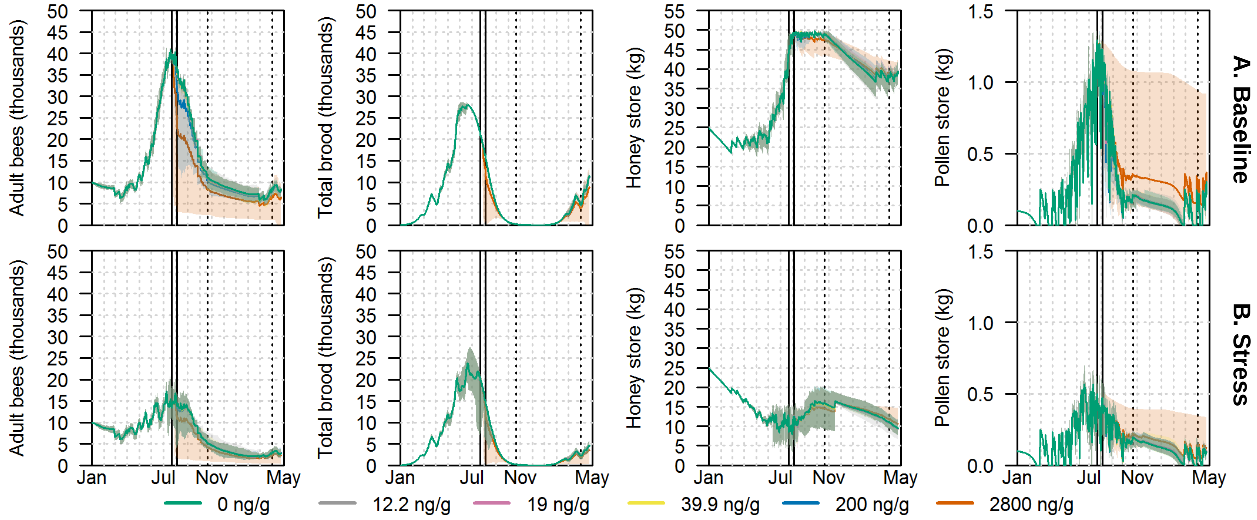

WI-04:

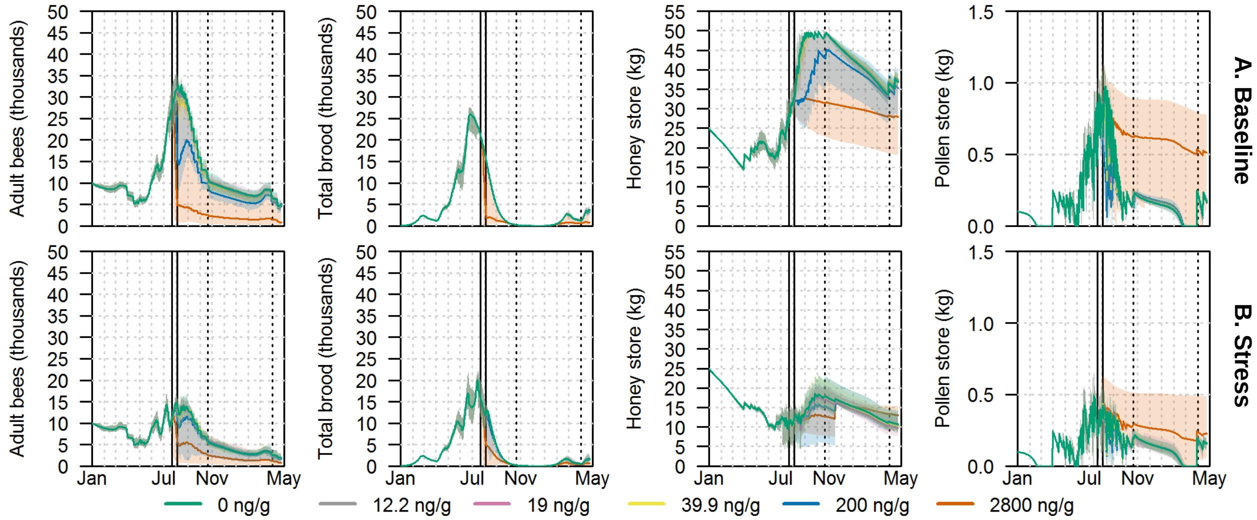

WI-05:

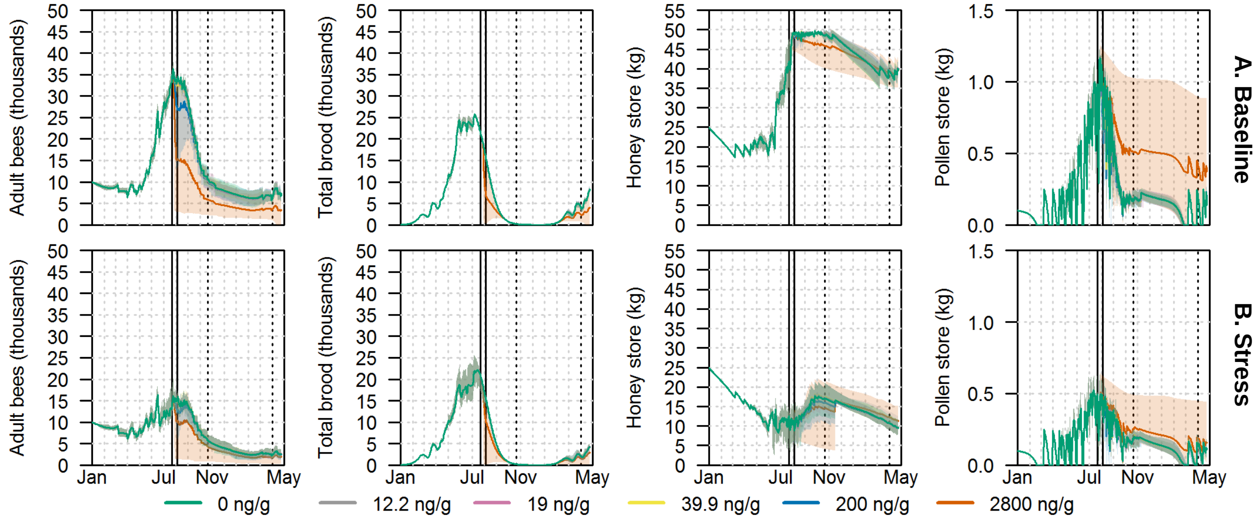

WI-06:

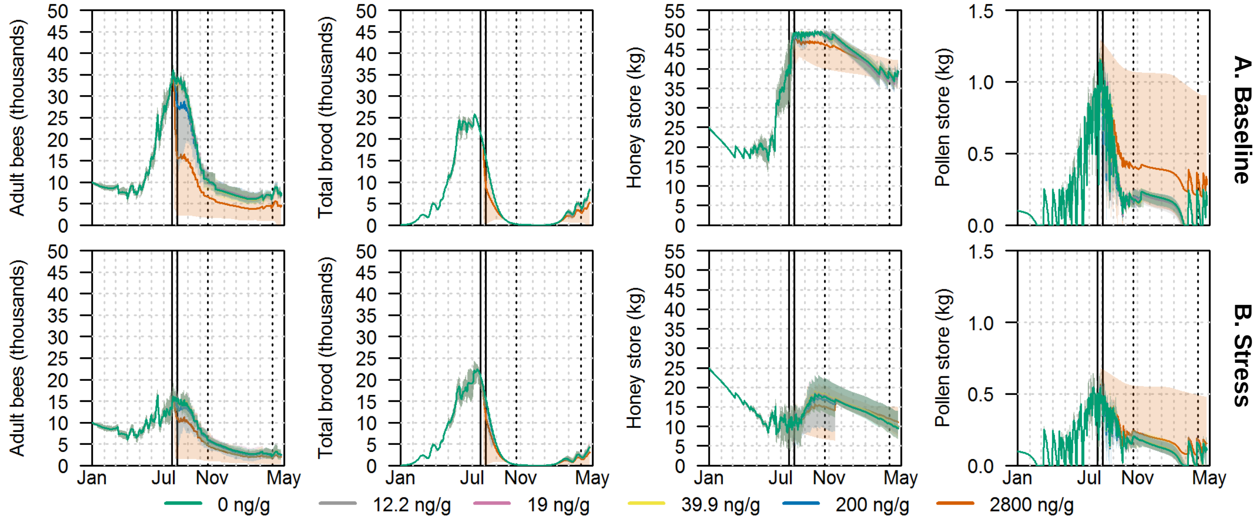

WI-07:

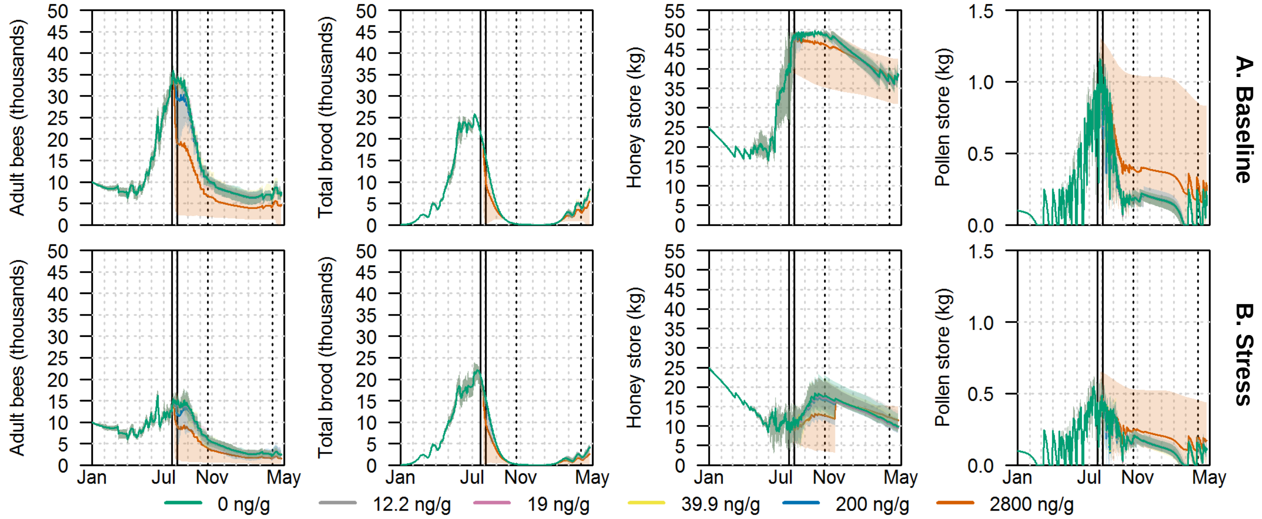
Adult bees (thousands)
Total brood (thousands)
Honey store (kg)
Pollen store (kg)
A. Baseline
B. Stress
Jan Jul Nov May
0 ng/g
12.2 ng/g
19 ng/g
39.9 ng/g
200 ng/g
2800 ng/g

WI-08:

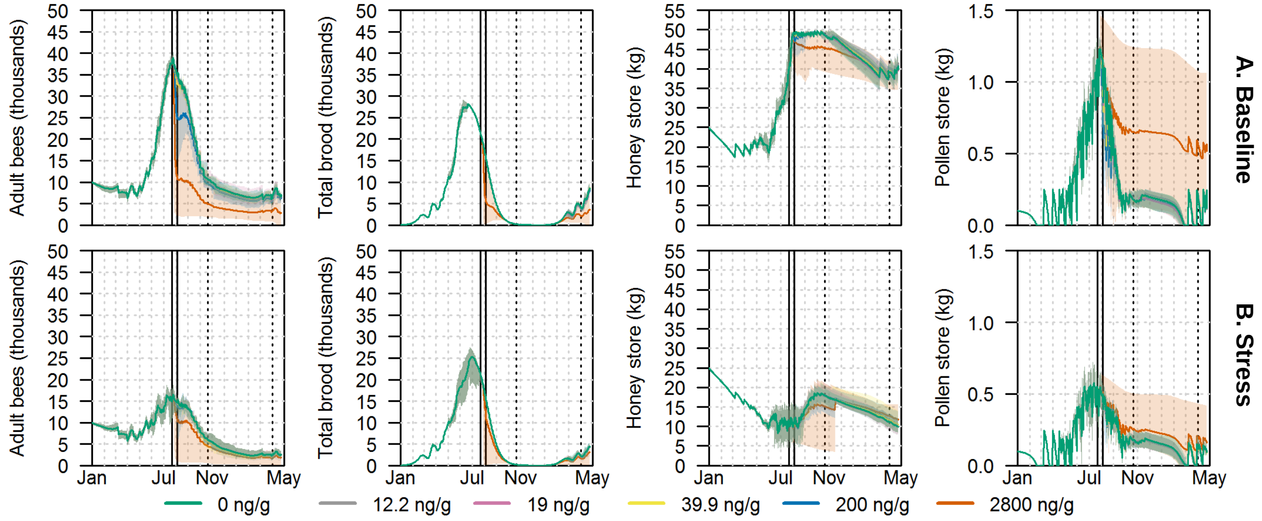
Adult bees (thousands)
Total brood (thousands)
Honey store (kg)
Pollen store (kg)
A. Baseline
B. Stress
Jan Jul Nov May
0 ng/g
12.2 ng/g
19 ng/g
39.9 ng/g
200 ng/g
2800 ng/g

7.2.2. Colony-level effects

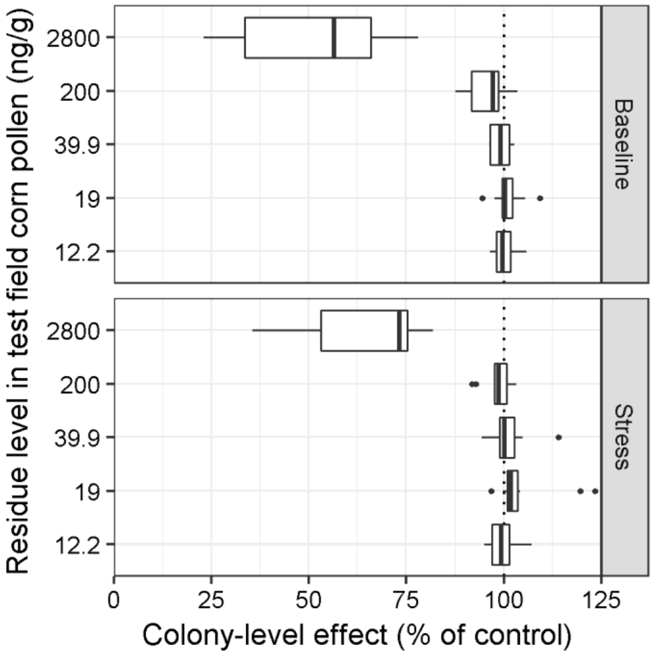

Fig. S5. Colony-level effects of simulated clothianidin residue levels in pollen from the test corn field. Percentage of adult bees in exposed compared to control hives on 1 April of year 2 are shown; compare to Fig. 4 in main manuscript showing results from 21 October of year 1. **Top**: Baseline scenario. **Bottom**: Stress scenario. Medians as percentage of control are shown, and boxes delineate the interquartile range (IQR), showing the 25th and 75th percentiles. Whiskers extend up to 1.5 times the IQR, and values beyond the whiskers are depicted as points.

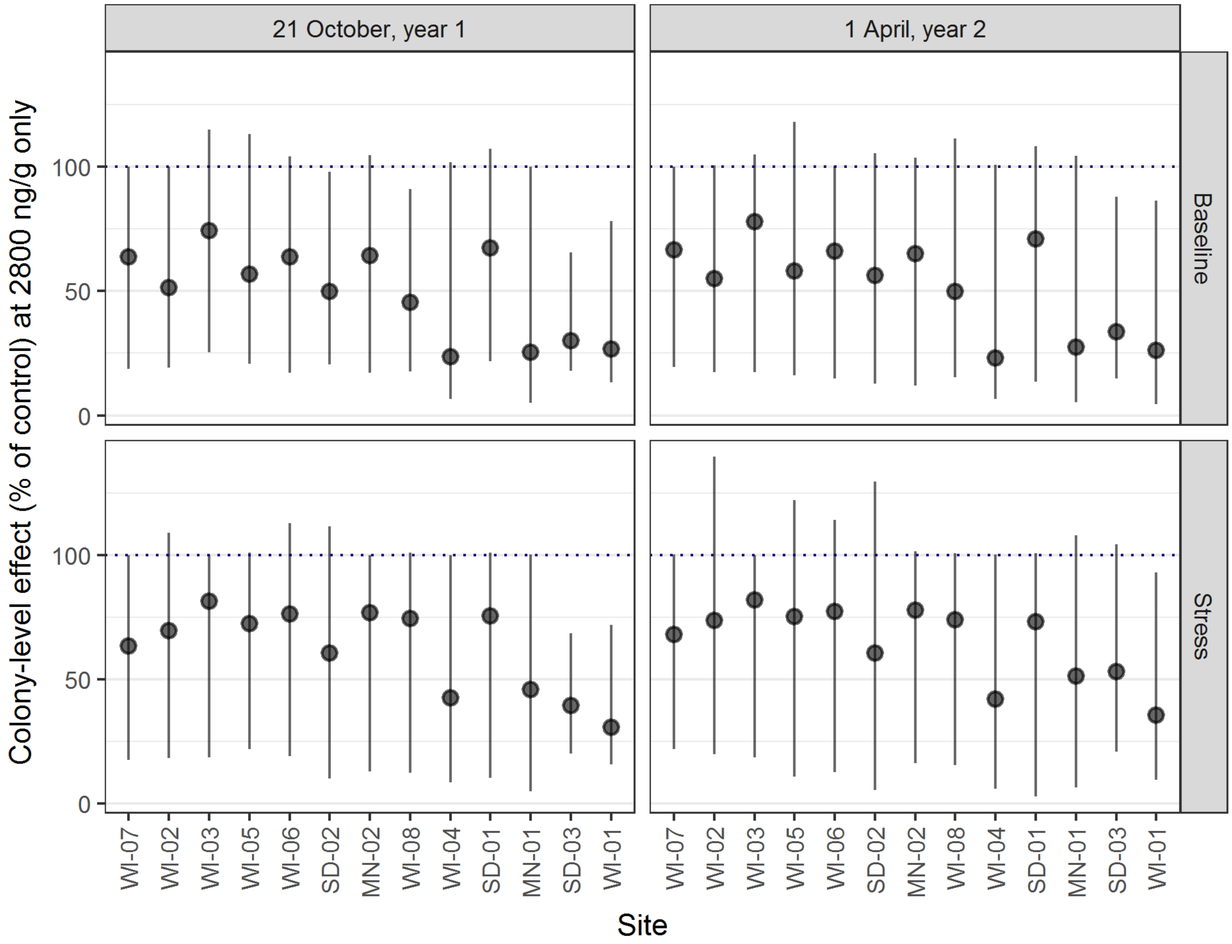

Fig. S6. At only the 2800 ng/g residue level in test field corn pollen, colony-level effects by site on two dates and in the two scenarios as labeled. Sites are ordered by most to least semi-natural land, matching Figure 2 in the main text. Points show the mean of 20 simulations, and whiskers show the 90th percentile interval (5th to 95th percentiles). The dotted line marks 100%, where a 2800 ng/g run and a control run had the same the number of adults on a given date.

7.2.3. Pollen foraging and colony-level effects

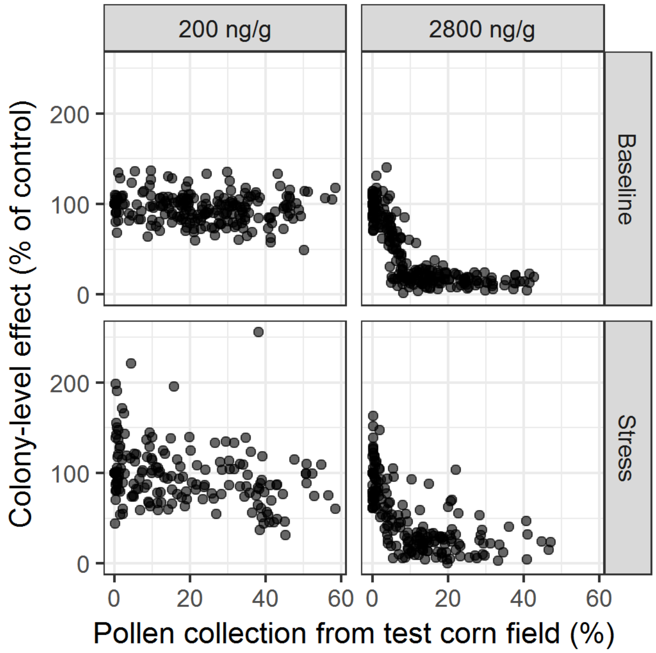

Fig. S7. Relationship between percentage of pollen collected from the test corn field compared to all pollen collected by each colony during corn tasseling (22 July-4 August) and colony-level effects (percentage of adult bees compared to controls on 1 April of year 2); compare to Fig. 5 in main manuscript showing results from 21 October of year 1. Each point represents the output from a single simulation run. **Left**: Relationship shown for simulations with 200 ng clothianidin/g pollen from test corn field. **Right**: 2800 ng clothianidin/g pollen from test corn field. **Top**: Baseline scenario. **Bottom**: Stress scenario.

8. MODEL OUTPUT CORROBORATION

The corroboration of the model outputs with independent field data (validation) was not part of the scope of the current paper. For the corroboration of the outputs, comprehensive data sets from field studies with honey bee colonies would need to be available. Such data sets should include detailed measurements on honey bee colonies over extended time periods, including bee and brood numbers and honey and pollen stores in the hives. Ideally, sources of pollen and nectar should be determined (e.g. from analysis of pollen brought back to the hive by foragers or bee dance directions) along with surveys of resource availabilities in the landscape. For the corroboration of exposure and effects, exposures across the landscape would have to be measured or estimated from land use practices, and residue levels in hive-stored pollen (beebread) and nectar/honey would need to be measured. Data sets allowing the comparisons of untreated controls and treated colonies (or colonies exposed to treated fields) would need to be available.

References

Babendreier, D., Kalberer, N., Romeis, J., Fluri, P. and Bigler, F. 2004. Pollen consumption in honey bee larvae: a step forward in the risk assessment of transgenic plants. Apidologie 35: 293-300.

Bagavathiannen, M.V. 1999. Feral nature of alfalfa (*Medicago sativa* L.): implications for novel trait confinement. PhD Thesis, Department of Plant Science, University of Manitoba, Winnipeg, MB, Canada.

Baude, M., Kunin, W. E., Boatman, N. D., Conyers, S., Davies, N., Gillespie, M. A. K., Morton, R. D., Smart, S. M., Memmott, J. 2016. Historical nectar assessment reveals the fall and rise of floral resources in Britain. Nature, 530(7588), 85-88.

Becher, M.A., Grimm, V., Knapp, J., Horn, J., Twiston-Davies, G., Osborne, J.L. 2016. BEESCOUT: A model of bee scouting behaviour and a software tool forcharacterizing nectar/pollen landscapes for BEEHAVE. Ecol. Model. 340, 126–133.

Becher MA, Grimm V, Thorbek P, Horn J, Kennedy PJ, Osborne JL. 2014. BEEHAVE: a systems model of honeybee colony dynamics and foraging to explore multifactorial causes of colony failure. J Appl Ecol 51:470–482.

Björkman, T., Pearson, K. 1995. The inefficiency of honeybees in the pollination of buckwheat. Current Advances in Buckwheat Research 1995: 453-462

Cane, J., Gardner, D., Harrison, P. 2011. Nectar and pollen sugars constituting larval provisions of the alfalfa leaf-cutting bee (*Megachile rotundata*) (Hymenoptera: Apiformes: Megachilidae). Apidologie, 42: 401-408.

Carter, P.R., Hicks, D.R., Oplinger, E.S., Doll, J.D., Bundy, L.G., Schuler, R.T., Holmes, B.J. 1989. Grain Sorghum (Milo). Alternative Field Crops Manual. https://www.hort.purdue.edu/newcrop/afcm/sorghum.html

Cawoy, V., Deblauwe, V., Halbrecq, B., Ledent, J.-F., Kinet, J.-M., Jacquemart, A.-L. 2006. Morph differences and honeybee morph preference in the distylous species *Fagopyrum esulentum* Moench. Int. J. Plant Sci. 167(4): 853-861

Cawoy, V., Kinet, J.-M., Jacquemart, A.-L. 2008. Morphology of Nectaries and Biology of Nectar Production in the Distylous Species *Fagopyrum esculentum*. Annals of Botany 102 (5): 675–84.

Chiari, W. C., de Toledo, V. A. A., Ruvolo-Takasusuki, M. C. C., Attencia, V. M., Martins Costa, F., Kotaka, C. S., Sakaguti, E. S., Magalhães, H. R. 2005. Floral biology and behavior of Africanized honeybees *Apis mellifera* in soybean (*Glycine max* L. Merril). Braz. Arch. biol. Technol. 48(3)[3]

Dittberner, P. L., Olson, M. R. 1983. The plant information network (PIN) data base: Colorado, Montana, North Dakota, Utah, and Wyoming. FWS/OBS-83/86. Washington, DC: U.S. Department of the Interior, Fish and Wildlife Service. 786 p.

Dively, G.P., Embrey, M.S., Kamel, A., Hawthorne, D.J. and Pettis J.S. 2015. Assessment of chronic sublethal effects of imidacloprid on honey bee colony health. PLoS ONE 10(3): e0118748

EFSA (European Food Safety Authority). 2016. A mechanistic model to assess risks to honeybee colonies from exposure to pesticides under different scenarios of combined stressors and factors. EFSA supporting publication 2016:EN-1069. 116 pp.

[3] Available online: http://www.scielo.br/scielo.php?pid=S1516-89132005000300006&script=sci_arttext

Gerik, T., Bean, B., Vanderlip, R. 2003. Sorghum growth and development. Texas Cooperative Extension. The Texas A&M University System. B-6137. amarillo.tamu.edu/files/2010/11/sorghum_growth_development.pdf

Grimm, V., Berger, U., Bastiansen, F., Eliassen, S., Ginot, V., Giske, J., Goss-Custard, J., Grand, T., Heinz, S. K., Huse, G., Huth, A. and et al. 2006. A standard protocol for describing individual-based and agent-based models. Ecological Modelling, 198, 115–126.

Grimm, V., Berger, U., DeAngelis, D. L., Polhill, J. G., Giske, J. and Railsback, S. F. 2010. The ODD protocol: A review and first update. Ecological Modelling, 221, 2760–2768.

Grimm, V., Augusiak, J., Focks, A., Frank, B. M., Gabsi, F., Johnston, A. S. A., Liu, C., Martin, B. T., Meli, M., Radchuk, V., Thorbek, P. and Railsback, S. F. 2014. Towards better modelling and decision support: Documenting model development, testing, and analysis using TRACE. Ecological Modelling, 280, 129–139

Herbert, E.W., Shimanuki, H. 1978. Chemical composition and nutritive value of bee-collected and bee-stored pollen. Apidologie, 9: 33-40.

Hines, H. M., Hendrix, S. D. 2005. Bumble bee (Hymenoptera: Apidae) diversity and abundance in tallgrass prairie patches: effects of local and landscape floral resources. Environ. Entomol. 34(6): 1477-1484.

Jarosz, N., Loubet, B., Durand, B., Foueillassar, X., Huber, L. 2005. Variations in maize pollen emission and deposition in relation to microclimate. Environmental Science & Technology 39: 4377-4384.

Koti S., Reddy K. R., Reddy V. R., Kakani V. G. and Zhao D. 2004. Interactive effects of carbon dioxide, temperature, and ultraviolet-B radiation on soybean (Glycine max L.) flower and pollen morphology, pollen production, germination, and tube lengths. J Exp Bot 56: 725-736.

Nicolson, S.W. 2011. Bee food: the chemistry and nutritional value of nectar, pollen and mixtures of the two. African Zoology, 46: 197-204.

Oberhauser, K.S., Prysby, M.D., Mattila, H.R., Stanley-Horn, D.E., Sears, M.K., Dively, G., Olson, E., Pleasants, J.M., Lam, W.-K.F., Hellmich, R.L. 2001. Temporal and spatial overlap between monarch larvae and corn pollen. PNAS 98, 11913-11918.

Odoux, J.-F., Feuillet D., Aupinel, P., Loublier, Y., Tasei, J.-N., Mateescu, C. 2012. Territorial biodiversity and consequences on physico-chemical characteristics of pollen collected by honey bee colonies. Apidologie 43, 561-575

Osborne, J. L., Awmack, C. S., Clark, S. J., Williams, I. H., Mills, V. C. (1997). Nectar and flower production in Vicia faba L (field bean) at ambient and elevated carbon dioxide. Apidologie, 28, 43-55.

Palmer R. G., Albertsen M. C., Heer H. 1978. Pollen production in soybeans with respect to genotype, environment, and stamen position. Euphytica 27, 427-433.

Pavek, P.L.S. 2016. Plant Guide for buckwheat (*Fagopyrum esculentum*). USDA-Natural Resources Conservation Service, Pullman Plant Materials Center. Pullman, WA.

Prieto-Baena, J.C., Hidalgo, P.J., Dominguez, E., Galan, C. 2003. Pollen production in the Poaceae family. Grana 42: 153-159.

Reddi, C.S., Reddi, N.S. 1986. Pollen production in some anemophilous angiosperms. Grana 25:55-61.

Sabatini, A.G., Marcazzan, G.L., Caboni, M.F., Bogdanov, S., Bicudo de Almeida-Muradian, L. 2009. Quality and standardisation of Royal Jelly. J. ApiProduct ApiMedical Sci. 1, 1–6.

Schmidt, M.R., Bothma, G. 2005. Indications of Bee Pollination in Sorghum and its implications in transgenetic biosafety. ISMN 46: 72-75.

Schmolke, A., Thorbek, P., DeAngelis, D. L., Grimm, V. 2010. Ecological models supporting environmental decision making: a strategy for the future. Trends in Ecology and Evolution, 25, 479-486

Southwick, E.E. 1984. Photosynthate allocation to floral nectar: a neglected energy investment. Ecology, 65: 1775-1779.

Thompson, H., Coulson, M., Ruddle, N., Wilkins, S., Harkin, S. 2016. Thiamethoxam: Assessing flight activity of honeybees foraging on treated oilseed rape using radio frequency identification technology. Environ Toxicol Chem, 35: 385–393.

Uribellarea, M., Carcova, J., Otegui, M.E. and Westgate, M.E. 2002. Crop physiology & metabolism. Pollen production, pollination dynamics, and kernel set in maize. Crop Science 42: 1910-1918.

USDA (US Department of Agriculture). 2015. Attractiveness of agricultural crops to pollinating bees for the collection of nectar and/or pollen.

USEPA (US Environmental Protection Agency). 2012. White paper in support of the proposed risk assessment process for bees. Submitted to the FIFRA Scientific Advisory Panel for Review and Comments, September 11-14, 2012.

USEPA (US Environmental Protection Agency). 2016. Benchmark Dose Software (BMDS).

USEPA (US Environmental Protection Agency). 2014. Guidance for Assessing Pesticide Risks to Bees.

Vonhof, M.J., Harder, L.D. 1995. Size-number trade-offs and pollen production by Papilionaceous legumes. American Journal of Botany 82(2): 230-238.

Winston, M.L. (1987) The biology of the honey bee. Harvard University Press, Cambridge, MA.
