# Supplementary material for: Honey bee colony‐level exposure and effects in realistic landscapes: An application of BEEHAVE simulating clothianidin residues in corn pollen
Source: Environ Toxicol Chem. 2019 Jan 7;38(2):423–35. doi: 10.1002/etc.4314 (PMC6850421; doi:10.1002/etc.4314)
Supplement: Supplementary file 2 — Supporting Data S2. [file ETC-38-423-s002.zip › README_BEEHAVE_PEEM.rtf]

Schmolke et al.: Honey bee colony-level exposure and effects in realistic landscapes: an application of BEEHAVE simulating clothianidin residues in corn pollen 

Supplemental Data: Model code 

NetLogo model: BEEHAVE_BeeMapp2015_PEEM.nlogo
·	Extended BEEHAVE model based on the BEEHAVE version 'BEEHAVE_BeeMapp2015' including the pollen exposure-effects module
·	Requires NetLogo 5 to be opened and run (https://ccl.northwestern.edu/netlogo/); BEEHAVE is currently not compatible with later versions of NetLogo (version 6); simulations for the manuscript were run with version 5.3.1
·	Simulations conducted for the manuscript are included in 'BehaviorSpace'. A set of input files to run the example for WI-08 are included in the supplemental information. The input files necessary to run the remaining experiments are available from the corresponding author upon request (Amelie Schmolke, email: schmolkea@waterborne-env.com), and are specified below.
Landscape resource input files: 'INPUT_FILE' on BEEHAVE interface
·	156 text files
·	File names are set up as follows:
<site ID>_<Year>_<Radius>_<Scenario>_<Exposure>_<Date generated>.txt
Explanations and values used:
site ID: identification of the 13 locations applied in the simulations; MN-01, MN-02, SD-01, SD-02, SD-03, WI-01, WI-02, WI-03, WI-04, WI-05, WI-06, WI-07, WI-08
Year: year of CDL data used to generate the files; 2016 for all
Radius: radius around simulated colony location, i.e. patches within this radius are represented in the files; 1500m for all
Scenario: identification of scenario applied and described in the manuscript; 'GatSbaseline' for the baseline scenario, 'GatSrealistic' for the stress scenario
Date generated: date the files were generated in format YYYYMMDD; 20170927 for all
Weather input files: 'WeatherFile' on BEEHAVE interface
·	13 text files
·	File names are set up as follows:
Weather_<site ID>_<Year>_input.txt
Explanations and values used:
site ID: identification of the 13 locations applied in the simulations; MN-01, MN-02, SD-01, SD-02, SD-03, WI-01, WI-02, WI-03, WI-04, WI-05, WI-06, WI-07, WI-08
Year: year of weather data used to generate the files; 2016 for all
